# Analysis of multi-drug cancer nanomedicine

**DOI:** 10.1038/s41565-025-01932-1

**Published:** 2025-05-15

**Authors:** Karina Benderski, Twan Lammers, Alexandros Marios Sofias

**Affiliations:** 1 Department of Nanomedicine and Theranostics, Institute for Experimental Molecular Imaging (ExMI), RWTH Aachen University Hospital, Aachen, Germany; 2 Center for Integrated Oncology Aachen (CIOA), RWTH Aachen University Hospital, Aachen, Germany

**Keywords:** Nanoparticles, Drug delivery

## Abstract

Multi-drug nanomedicine is gaining momentum for co-delivering more than one drug to the same site at the same time. Our analysis of 273 pre-clinical tumour growth inhibition studies shows that multi-drug nanotherapy outperforms single-drug therapy, multi-drug combination therapy, and single-drug nanotherapy by 43, 29 and 30%, respectively. Combination nanotherapy also results in the best overall survival rates, with 56% of studies demonstrating complete or partial survival, versus 20–37% for control regimens. Within the multi-drug nanomedicine groups, we analysed the effect of (co-)administration schedule and strategy, passive versus active targeting, nanocarrier material and the type of therapeutic agent. Most importantly, it was found that co-encapsulating two different drugs in the same nanoformulation reduces tumour growth by a further 19% compared with the combination of two individually encapsulated nanomedicines. We finally show that the benefit of multi-drug nanotherapy is consistently observed across different cancer types, in sensitive and resistant tumours, and in xenograft and allograft models. Altogether, this meta-analysis substantiates the value of multi-drug nanomedicine as a potent strategy to improve cancer therapy.

## Abstract (web summary)

Multi-drug nanotherapy consistently outperforms single-drug therapy, multi-drug combination therapy, and single-drug nanotherapy across different experimental settings, therapy strategies and cancer types.

## Main

### Drug combinations in cancer therapy and nanomedicine

The multifaceted nature of cancer necessitates the exploration and use of different treatment modalities. In routine clinical practice, patients typically undergo a combination of interventions, aiming for an optimal therapeutic outcome^1^. Such scenarios not only entail the combination of two completely different therapeutic modalities (for example, surgery and chemotherapy), but also combine multiple therapeutic sub-options within the same treatment category. For example, neoadjuvant chemotherapy often encompasses a cocktail of chemotherapeutic drugs, aiming to achieve maximal tumour shrinkage before surgery^2^. Furthermore, drug combination therapies can complementarily address pathways of cancer progression at the level of cancer cells, immune cells and/or the tumour microenvironment^3,4^.

Despite the generally positive effects of combining different chemotherapeutics, combination therapy is challenging, as differences in physicochemical and pharmacokinetic properties between different drugs lead to uncoordinated targeting, efficacy and toxicity. Hence, dosing and scheduling an optimal drug combination regimen is difficult. Multiple different drug delivery systems have been designed and evaluated over the years to help correct this disconnect^5^. The success of nanomedicines relies on characteristics that include improved drug stability and solubility, targeted delivery to the pathological site, controlled drug release, and low off-target localization^6^. The versatile properties of nanocarriers make them suitable for delivering a plethora of different drugs to diseased tissues. Nanoformulations can furthermore be readily engineered to co-encapsulate two or more drugs to enable co-delivery to the same target cell, and/or tissue compartment, at the same point in time—ideally achieving synergistic therapeutic efficacy^7^. A clinically relevant example showcasing the ability of nanomedicines to co-encapsulate multiple drugs is Vyxeos. Vyxeos is a liposomal formulation that combines cytarabine and daunorubicin in a fixed 5:1 ratio; it is approved as a first-line treatment for patients suffering from acute myeloid leukaemia (AML) with myelodysplasia-related changes, as well as for therapy-related AML. The co-encapsulation of the two drugs at this fixed synergistic ratio produces clinically substantial improvements in overall survival in patients with a very poor prognosis and very high medical need^8^.

Combination strategies have not only been successful for the co-delivery of chemotherapy agents, but also for the combination of other drug classes. For example, immunomodulatory drug combinations have been applied in relapsing-remitting multiple sclerosis. In this context, Copaxone is a heterogenous mixture of millions of different polypeptides, with each polypeptide consisting of four amino acids co-synthesized in fixed molar ratios but at random sequences. The immunomodulatory effect of Copaxone is attributed to the similarities of its chemical composition to the myelin basic protein—one of the autoantigens implicated in multiple sclerosis. Copaxone acts as an immunosuppressor and helps to attenuate pathological inflammatory processes in multiple sclerosis lesions, thereby reducing the frequency of relapses in patients with relapsing-remitting multiple sclerosis^9,10^.

The positive effect of co-encapsulating two compounds in one (nano)formulation has also been explored in mRNA delivery. Specifically, two mRNAs encoding for different fluorescent proteins were encapsulated in lipid nanoparticles either together or separately, in fixed ratios. Co-encapsulation enabled the delivery of both mRNAs into the same cells at the desired ratio, whereas separate encapsulation led to dissimilar cellular uptake and variable protein expression^11^. Similarly, a bottlebrush polymer prodrug was recently reported in which the three multiple myeloma drugs bortezomib, pomalidomide and dexamethasone are co-formulated. The therapeutic efficacy of administering a statistical mixture of three-drug polymer nanotherapy with synergistic drug ratios was compared with that of co-administering three single-drug polymer prodrugs at identical amounts. The three-drug co-formulation potently outperformed single-drug co-administration, even when lower total doses of the former were applied^12,13^.

Despite the widely anticipated advantages of nanomedicine-based combination therapy, its benefits have not yet been analysed in a comprehensive and quantifyable manner. Here we therefore set out to systematically study the value of multi-drug cancer nanomedicine combination therapy compared with free drugs, free drug combinations and single nanodrug therapy in pre-clinical mouse models. To this end, we screened the pre-clinical literature and identified 742 unique manuscripts, of which 273 studies enabled us to comprehensively compare therapeutic efficacy results (tumour growth inhibition and survival) of multi-drug nanotherapy to three relevant control groups: single free drug therapy, free drug combination therapy and single-drug nanotherapy. We also compared the efficacy of dual-drug nanomedicine co-formulation versus two single-drug nanomedicines. For comprehensive understanding, we finally also analysed the impact of treatment schedule, tumour type, drug resistance, immunological status and targeting strategy on the added value of multi-drug cancer nanomedicine combination therapy.

### Systematic analysis of ‘combination cancer nanotherapy’ in literature

**Fig. 1:**
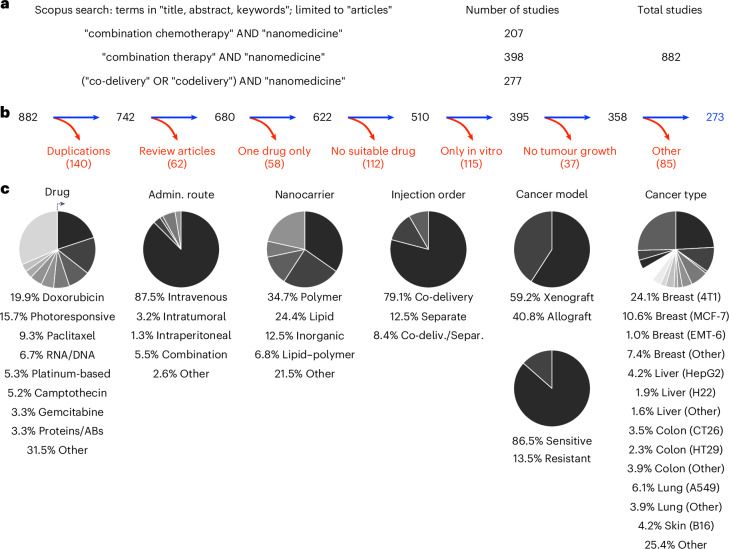
Meta-analysis methodology. **a**, Search terms used on scopus.com to identify research articles focusing on multi-drug nanomedicine combination therapy. **b**, Inclusion versus exclusion of studies focusing on cancer nanomedicine combination therapy. ‘Other’ exclusion criteria include reasons such as use of non-murine animal models, and investigation of diseases other than cancer. **c**, Pie charts based on data collected from the 273 included research articles illustrate details regarding the type of drug used, route of administration, nanocarrier formulation, injection protocol, cancer model and cancer type. The allocated groups (listed below the charts) are represented in the clockwise direction, starting from the top. Admin., administration; AB, antibody.

All available research papers on combination nanotherapy were collected via a thorough literature search in the scopus.com database. This search, based on three groups of relevant keywords (Fig. 1a and Supplementary Fig. 1), yielded 882 results addressing multi-drug cancer nanomedicine combination therapy. After applying the relevant exclusion criteria, a total of 273 suitable manuscripts (see Supplementary Table 1) were selected for in-depth analysis (Fig. 1b).

By digging into experimental details, we identified several interesting study characteristics (Fig. 1c). Doxorubicin was by far most used drug, often serving as a prototype drug to validate the effect of the combination therapy upon combining it with a second and/or third drug. This finding is in line with current clinical practice, as doxorubicin is very widely used and was historically the first chemotherapy drug that was approved in liposomal formulations (that is, Doxil/Myocet)^14,15^. Other drugs that have been very extensively used are paclitaxel and platinum-based drugs, in line with their widespread use as first- or second-line chemotherapy for various cancers in the clinic^16^. Regarding route of administration, the evaluated nanochemotherapeutics are largely injected intravenously, which is indeed the most common administration route in clinical practice^17^. Considering the composition material of nanocarriers, lipids and polymers were the most abundantly used. Somewhat surprisingly, there were slightly more papers on polymeric combination nanotherapy than on liposomal multi-drug therapy, in spite of the fact that most clinical products are based on liposomes (for example, Doxil, DaunoXome, DepoCyt and Onivyde)^5^. With respect to drug loading and injection regimen, formulations that simultaneously contained two drugs were more frequent in comparison to a mix of single-loaded nanomedicines.

In terms of the cancer models employed to evaluate combination nanotherapy, most pre-clinical models were xenografts, in which human cancer cell lines and patient-derived cancer cells are inoculated in immunodeficient mice (Fig. 1c). These models have the limitation that they do not allow for full consideration immunological (nano/chemo)therapy effects^18^. Syngeneic allograft tumour models can be grown in immunocompetent mice, but do not allow for use of human cancer cells^19^. With regard to intrinsic drug sensitivity, the majority of studies were conducted in chemotherapy-sensitive models, and only 14% of experiments focused on combination nanotherapy as a means to counteract multi-drug drug resistance.

Finally, in line with the long tradition in nanomedicine to target breast cancer (probably due to ease of model development, as well as wide occurrence in the global population)^20^, the 4T1 triple-negative breast cancer model was found to be the by far most frequently used tumour model (Fig. 1c). Its prevalence was more than twice as high as that of the second-highest-used model. The 4T1 model has several characteristics that make it an attractive experimental mouse model: it is a robust, rapidly growing, syngeneic and orthotopic model that can metastasize spontaneously, and closely resembles human triple-negative breast cancer^21^.

### Multi-drug cancer nanotherapy boosts treatment outcome

**Fig. 2:**
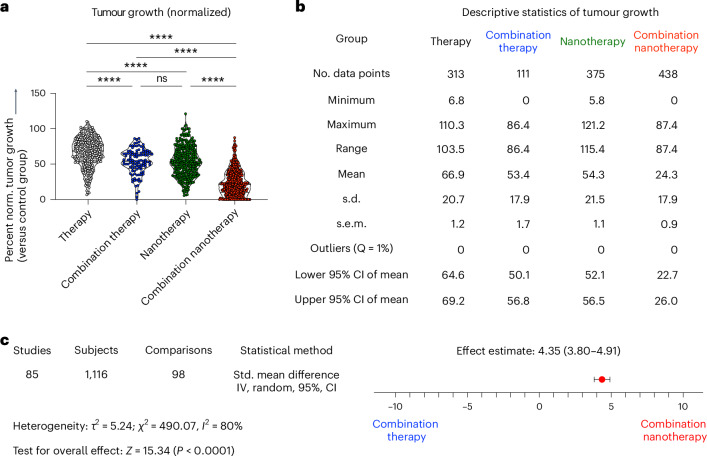
Efficacy analysis of multi-drug cancer nanotherapy. **a**, Direct comparison of the antitumor efficacy of (1) free drugs alone, (2) free drug combination therapy, (3) nanodrugs alone, and (4) nanodrug combination therapy. Our analysis demonstrates that multi-drug nanomedicines outperform the other treatment groups. Statistical significance was assessed via a two-sided Kruskal–Wallis test with Dunn’s correction for multiple comparisons (****P < 0.0001, ***P < 0.001, **P < 0.01, *P < 0.05, ns, not significant). **b**, Overview of descriptive statistics among the four groups demonstrated a 42.6%, 29.1% and 30.0% additional tumour inhibition enhancement for combination nanotherapy versus single free-drug, free drug combination therapy, and single-drug nanotherapy, respectively. **c**, Meta-analysis comparing combination therapy to combination nanotherapy. Pooled results from 98 comparisons with 1,116 mice, indicated an effect in favour of combination nanotherapy. Heterogeneity tests showed high heterogeneity, with *l*^2^ = 80%. This probably results from the intrinsic variety between the included studies regarding tumour models, therapies, treatment schedules and nanoparticle designs. Meta-analysis was performed by measuring the standardized (Std.) mean difference, using an inverse variance (IV) and random effects model. Heterogeneity between the groups was investigated using *τ*^2^ and *χ*^2^ tests and the *I*^2^ index.

To evaluate the efficacy of multi-drug combination nanotherapy, we quantified the therapeutic efficacies of: (1) single free drugs, (2) free drug combinations, (3) single nanodrugs and (4) combination nanodrugs, and compared them against tumour growth observed in the PBS/vehicle control group (Fig. 2a,b). From this analysis, it can be robustly concluded that: (i) drug combinations are almost always better than single therapies—both for free drug and for nanomedicine-based therapies. Different drugs can target different pathways, resulting in improved therapy outcomes^22^; (ii) the combination of two free drugs is more efficient than single free drugs, reducing tumour growth to 53.4% versus 66.9% of controls, respectively; (iii) the combination of drugs delivered by nanomedicines very efficiently suppresses tumour growth, to only 24.3% on control tumours growth. For single-drug nanotherapy, tumour growth is reduced to 54.3% of controls.

Upon studying the results in Fig. 2a,b more comprehensively, we find that: (iv) compared with free drug therapy, single-drug formulation in nanomedicines enhances the therapy outcome by 12.6%. Carriers can be used to deliver a plethora of therapeutics, such as hydrophilic or hydrophobic drugs, small drug molecules, peptides and proteins. Nanomedicine encapsulation is known to enhance drug circulation half-life and target site accumulation, thereby promoting therapeutic outcomes in mouse models^23^. (v) Combining two drugs in free form is as effective as single-drug nanotherapy. The growth inhibition induced by both regimens was found to be almost similar, that is, 53.4% and 54.3% of controls. Finally, and most importantly, (vi) combination nanotherapy outperformed all other regimens, with a further 42.6%, 30% and 29.1% stronger inhibition of tumour growth compared with single free-drug therapy, single-drug nanotherapy and free drug combination therapy, respectively. This probably comes from nanocarriers’ ability to contain drugs in defined ratios and allow for controlled and sustained drug release^24^. One of the major reasons behind combination nanotherapy's success lies in its improved circulation half-life and biodistribution profile. Specifically, nanomedicines have been shown to prolong the circulation half-life of drug molecules by ninefold, and they enable fivefold-higher drug accumulation in tumours compared with their free drug counterparts (Supplementary Fig. 2). Furthermore, nanomedicine co-encapsulation aligns temporal and spatial targeting to the same cell and/or same tumour compartment, thereby promoting synergy^7^. Further analysis was performed to evaluate whether there are differences in outcomes between studies that investigated potential synergy between the used drugs and those that did not. Out of the 273 studies, 86 actively investigated synergistic interactions of drug combinations. However, no discernible difference could be observed between the two groups (Supplementary Fig. 3a,b). Nevertheless, it should be noted that in all studies except one, the total drug dose was the same for monotherapy and combination therapy, which means that a higher overall drug dose was used in the combination therapy group. Potential synergies in drug combinations could eventually be exploited to help reduce overall drug doses, and they could assist in improving drug compliance and tolerability, making combination therapy a more effective and safer option overall.

With respect to the combination set-up, most studies selected a combination of two drugs, whereas some of them even attempted to combine three drugs. It was found that the added value of combinatorial nanotherapy was transferable to both two- and three-drug combination set-ups. As compared with two-drug combination nanotherapy, three-drug combination nanotherapy was found to provide an additional 6.5% reduction of tumour growth (Supplementary Fig. 4a,b).

The therapy studies analysed in this work displayed a high degree of heterogeneity with regard to planning and executing of the animal experiments. To address this, the studies were further divided according to (1) overall therapy duration, (2) the number of total therapy administrations, (3) the number of days between the final therapy administration and the assessment of the tumour's growth, and (4) time elapsed from the inoculation of the cancer cells until death of the control group (Supplementary Fig. 5). In all of these subgroup analyses, combination nanotherapy clearly prevailed, showing superior performance in terms of tumour growth inhibition than all other therapy groups (Supplementary Figs. 6–9).

Combination nanotherapy also resulted in the best overall survival of individual mice (Supplementary Fig. 10a). Following the same trends as for tumour growth inhibition, single free drug therapy results in the shortest median overall survival, followed by single-drug nanotherapy and free drug combination therapy. Combination nanotherapy clearly presented with the most favourable survival outcomes. To specify, in 87 cases of using combination nanotherapy, 16% achieved complete survival of the whole cohort by the end of the experiment, compared with only 2% of studies for the single-drug nanotherapy group. For single free drug treatment and free drug combination therapy, long-term survival was 0% in the studies included in this analysis (Supplementary Fig. 10b).

Based on these outcomes, it can be concluded that nanomedicines potentiate the efficacy of drug combinations. To substantiate this conclusion, we conducted an actual meta-analysis comparing the therapy efficacy of combination nanotherapy versus the combination therapy. To this end, tumour sizes, standard deviations and animal group sizes from mice treated with free drug cocktails (group 2) versus combination nanotherapy (group 4) were extracted from the tumour growth curves and inserted into the meta-analysis software RevMan^25^. This meta-analysis outcome provides the standardized mean difference, which is a statistical parameter to measure effect sizes, comparing free drug combination therapy and combination nanotherapy; it is expressed as both numbers and a forest plot (Fig. 2c). Each comparison presents an independent strictly standardized mean difference, which results in an overall average of 4.35 in favour of combination nanotherapy—clearly demonstrating benefits over non-nanomedicine-based combination therapy. By exploring this analysis in more detail, 22 comparisons gave no estimable effect size due to tumour volumes or standard deviations being zero, and so were therefore excluded. Out of the 98 comparisons included, eleven comparisons showed no effect in either direction, whereas one comparison showed an effect size in favour of free drug combination therapy. The remaining 86 comparisons showed a clear and highly significant benefit of combination nanotherapy versus free drug combination therapy (Fig. 2c, Supplementary Table 2 and Supplementary Fig. 11).

So far the concept of combination nanotherapy has led to the clinical approval of Vyxeos. Initially, the standard treatment regimen for patients with AML involved daily infusion of daunorubicin combined with a continuous infusion of cytarabine (3 + 7). This standard treatment led to up to 80% remission in younger patients, and to 60% in adults; however, only 30% of all patients achieved long-term disease-free survival. Several strategies have been explored to enhance treatment outcomes, including dose and administration schedule modifications, or adding a third drug, both of which did not result in increased overall survival. Pharmacokinetic and dynamic investigations of cytarabine and daunorubicin showed that synergistic ratios could not be maintained because these drugs have very different physicochemical properties, resulting in very different biodistributions, target site accumulations and target cell uptakes^26^. A liposomal formulation co-encapsulating both drugs at synergistic ratios was eventually developed, ensuring efficient cytarabine and daunorubicin co-delivery to the same body compartments and target cells. In a crucial phase clinical III study, a fixed 5:1 combination of the two drugs—compared with the standard 3 + 7 therapy in patients with AML—led to an increase in remission rate, as well as to a considerable prolongation of both event-free and overall survival^8,27^. Importantly, this trial also illustrated that combination nanotherapy provided improved efficacy at a lower cumulative drug dose compared with free drug combination therapy^5^.

The above clinical observations are in line with our outcomes of pre-clinical data analysis, compellingly indicating that future cancer therapy strategies should embrace multi-drug combination nanotherapy. To corroborate this notion, we expanded our analysis in specific subgroups of the initially analysed cohort, aiming to answer the following questions: is co-encapsulation of two drugs in one nanomedicine formulation more beneficial than two drugs administered in two separate nanocarriers? Which drug type combination works best for multi-drug nanomedicine application? Is there a superior nanocarrier material? Is active nanomedicine tumour targeting better than passive tumour targeting? Does PEGylation enhance combination nanotherapy outcomes? Do all tumour types benefit equally from combination nanotherapy?

### Nanomedicine co-delivery improves combination therapy outcome

**Fig. 3:**
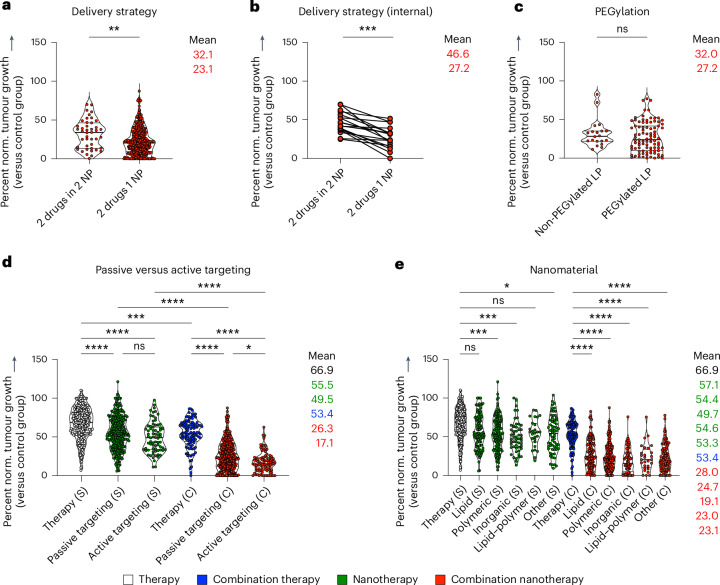
Multifactorial analysis of multi-drug cancer nanomedicine. **a**, Comparison of two common administration regimens in combination nanotherapy, showing that co-delivery (that is, two drugs in the same nanoformulation) outperforms separate delivery (that is, two drugs in two separate nanoformulations). **b**, Direct comparison of studies investigating co-encapsulation versus separate encapsulation in the same set-up, showing that in all cases except one, co-encapsulation achieves significantly better tumour growth inhibition. **c**, PEGylation did not affect the outcome of multi-drug cancer nanomedicine. **d**, Passive versus active targeting of single-drug and multi-drug nanotherapy indicates a trend towards improved tumour growth reduction for active targeting. **e**, Comparison of commonly used drug delivery formulations revealed that nearly all nanomaterial platforms can improve single- and multi-drug antitumor therapies. All mean values, from top to bottom, correspond with the violin plots from left to right. Statistical significance was assessed by two-tailed Mann–Whitney test (**a**–**c**), and a two-sided Kruskal–Wallis test with Dunn’s correction for multiple comparisons (**d**,**e**) (****P < 0.0001, ***P < 0.001, **P < 0.01, *P < 0.05). NP, nanoparticle; LP, lipid-based nanoparticle.

Combination therapy can be applied in two different ways: co-delivery (that is, simultaneous administration of two drugs that are co-encapsulated in the same formulation) and separate delivery (that is, two drugs are separately encapsulated, and two formulations are co-injected or sequentially administrated). After subdividing studies on the basis of the combination therapy strategy used, we found that most studies used co-delivery rather than separate delivery, and—importantly—that co-delivery outperformed separate delivery (**P = 0.0016) (Fig. 3a). A direct comparison of studies that performed both co-delivery and separate delivery in the same experiment revealed that in all cases except one, co-encapsulation significantly outperformed separate encapsulation in terms of tumour growth inhibition (***P = 0.0002) (Fig. 3b).

Co-encapsulation increases the possibility of two drugs entering the same cell at the same time in an optimized drug-to-drug ratio^12^. It thus creates an ideal framework for achieving synergistic anti-tumour effects, thereby boosting antitumor activity and explaining the outcomes of the analysis^24^. Although two single-loaded nanoparticles can reach tumour compartments simultaneously, it is quite unlikely that they are able to achieve ratiometric dosing at the single-cell level, as already confirmed for small molecules and nucleic acids^11,13^. Aside from cancer cells, other types of cells also need to be targeted simultaneously to promote synergistically improved anti-tumour efficacy. Several studies have already shown that antigens and immunostimulatory adjuvants, such as oligodeoxynucleotides, must be delivered in the same vehicle to ensure co-localization in antigen-presenting cells to induce strong antigen-specific immune responses^28^. When the aim is to prime the tumour microenvironment as well as deliver cytotoxic drugs, co-encapsulation may not be essential for improved outcomes^29–31^. This is because reducing physical barriers, such as the dense extracellular matrix, may require different timing and multiple treatment cycles for optimal efficacy^32,33^. Nonetheless, from the perspectives of intellectual property, pharmaceutical production and straightforward clinical translation, one could argue that dual-drug co-encapsulation remains preferable even in such cases.

We next compared lipid-based nanomedicines with and without PEG to examine whether stealth coatings have a positive influence on combination nanotherapy outcome. PEGylated formulations showed a slight but insignificant increase in tumour growth suppression (^ns^*P* = 0.2808) (Fig. 3c). PEGylation is extensively performed preclinically and clinically to shield nanoparticles against aggregation, opsonization and phagocytosis^34^. PEGylation, however, also has downsides, including induction of anti-PEG antibodies, non-biodegradability, a prolonged whole-body clearance time of the polymer, undesired side-product formation, and reduction of drug uptake by target cells^35,36^. Moreover, for certain cancer types—and particularly for non-solid tumours—PEGylation may not necessarily add much value, as exemplified by the fact that the dual-drug anti-leukaemia nanomedicine Vyxeos is not PEGylated. The pros and cons of nanoparticle PEG coating are generally referred to as PEG dilemmas, and various strategies have been implemented to overcome them^37,38^.

Another classical nanomedicine dilemma relates to the use of active targeting ligands. To address this, we directly compared the therapeutic efficacy of free drug single and combination therapy with that of nanotherapies employing either passive or active targeting, including both single-drug and nanomedicine combination therapies (Fig. 3d). Passive targeting of single nanodrugs resulted in statistically significant enhancement of tumour growth inhibition compared with single free drug therapy (*P* < 0.0001) (Fig. 3d). Actively targeted single-drug nanotherapy also outperformed single free drug therapy, but it was not statistically superior to passive targeting. With respect to combination therapy, passively targeted dual-drug nanomedicines produced a highly significant improvement in tumour growth inhibition compared with both free drug combination therapy and single-drug passive nanotherapy (*P* < 0.0001) (Fig. 3d). Actively targeted dual-drug nanomedicine therapy was found to be even more efficient, outperforming all of the other groups, including passively targeted multi-drug nanotherapy (*P = 0.0401) (Fig. 3d). In principle, active targeting is a very appealing concept; however, due to ligand decoration procedures, nanoparticles may lose some of their stealthy character. This may reduce plasma circulation times, as the modified nanoparticles are more likely to be recognized by phagocytes before reaching the target site and cell^39^. If ligand-modified nanomedicines do manage to efficiently reach tumour compartments, active targeting is expected to be favourable for dual-drug therapy, as the two drugs can then indeed be delivered into cancer cells at the intended synergistic ratio.

We next compared different nanocarrier materials as platforms for tumour-targeted drug delivery. Drug encapsulation in lipidic, polymeric, inorganic and lipid–polymer-hybrid materials in all cases resulted in improved therapeutic outcomes compared with free drug administration; however statistically significant differences were only observed for polymeric (***P = 0.0003), inorganic (***P = 0.0006) and ‘other’ materials (*P = 0.0124) (Fig. 3e). In dual-drug combination set-ups, all nanomaterial platforms reduced tumour growth significantly compared with free drug combinations (Fig. 3e). No significant differences were observed among the different carrier materials evaluated. We also explored the impact of the drug classes (co-)administrated in nanocarriers. For that purpose, we fractionated the studies in our analysis based on all common primary drug used, that is, anthracyclines, taxanes, platinum-based drugs, camptothecin (and derivatives), antimetabolites and tyrosine kinase inhibitors. We found that in all of these cases, combination nanotherapy significantly increased the efficacy of tumour growth inhibition by a further 41.6% compared with set-ups in which these major drugs were administered alone (Supplementary Fig. 12). Altogether, these detailed sub-analyses clearly show that multi-drug nanomedicine is favourable over all other regimens, including individually administered nanotherapy combinations, and that these beneficial effects are corroborated in studies looking at passive versus active targeting, at delivery via different nanocarrier material, and encapsulation of different therapeutic cargos.

### Multi-drug cancer nanomedicine efficacy in various tumour models

**Fig. 4:**
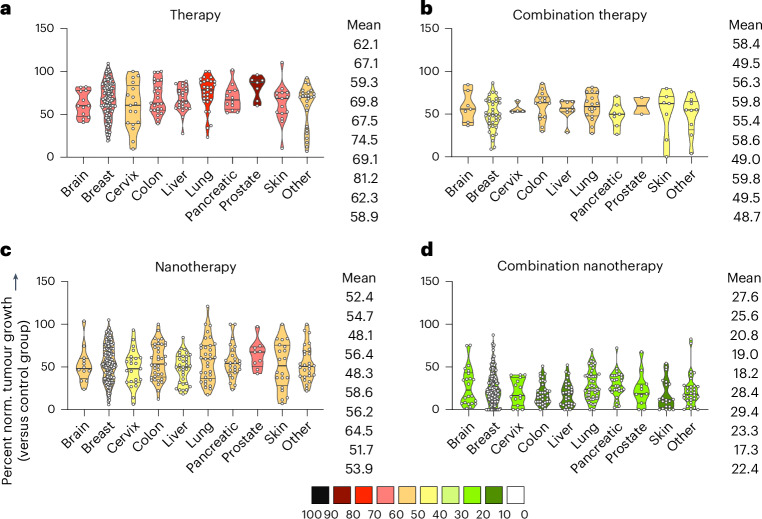
Analysis of multi-drug nanomedicine treatment efficacy in different cancer types. Different tumour models were compared for each group. Treatments were divided into single free drug therapy, free drug combination therapy, single-drug nanotherapy and combination nanotherapy. In each panel, mean normalized tumour growth reduction values are colour-coded. From top to bottom, the mean values for the different groups correspond to the violin plots from left to right. **a**, Single free drug therapy showed the lowest tumour reduction efficacy throughout all models. **b**, Free drug combination therapy improved the therapeutic response compared with single free drug therapy in all cancer types. **c**, Single-drug nanotherapy enhanced single free drug therapy to a similar extent as free drug combination therapy. **d**, Combination nanotherapy achieves maximal tumour reduction across all tumour types, clearly outperforming the other three regimens. Data are presented as violin plots depicting the mean, range, and 25%, 50% and 75% percentiles.

To evaluate whether the benefit of nanomedicine combination therapy is preserved among different tumour models, studies were systematically sorted according to the tumour model used. The treatment cohorts were again subdivided in single free drug therapy, single-drug nanotherapy, free drug combination therapy, and combination nanotherapy (Fig. 4). In comparison to untreated control, single-drug therapy showed the lowest tumour growth reduction efficacy throughout all tumour models (58.9–81.2%; Fig. 4a), with skin, cervix, brain and liver cancer exhibiting the highest (albeit statistically insignificant) response rates, followed by free drug combination therapy (48.7–59.8%; Fig. 4b) and single-drug nanotherapy (48.1–64.5%; Fig. 4c). Of note, single-drug nanotherapy was found to be superior to combination therapy in five out of the ten cancer models (Fig. 4b,c). Most importantly, combination nanotherapy showed the by far biggest reduction in tumour growth for all tumour types included, with a large proportion of cases even achieving full remission by the end of the experiment (17.3–29.4%; Fig. 4d).

**Fig. 5:**
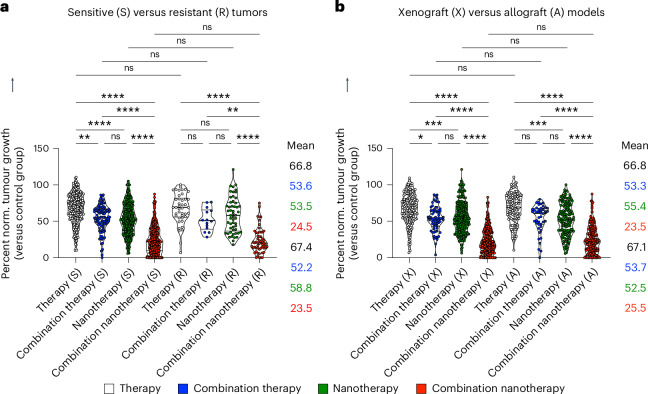
Analysis of (nano)drug combination therapy in sensitive versus resistant and xenograft versus allograft tumours. **a**, Sensitive and resistant tumours are both treated most efficiently by combination nanotherapy. For resistant tumours, free drug combination therapy and single-drug nanotherapy showed no benefit over single free-drug therapy. Multi-drug combination nanotherapy was the only treatment that significantly reduced tumour growth in resistant models. **b**, In xenograft versus allograft models, therapy regimens produced very similar performance. In both cases, combination nanotherapy was found to be the by far the most effective treatment. Data are presented as violin plots depicting the mean, range, and 25%, 50% and 75% percentiles. Mean values, from top to bottom, correspond with the violin plots from left to right. Statistical significance was determined by a two-sided Kruskal–Wallis test with Dunn’s correction for multiple comparisons (****P < 0.0001, ***P < 0.001, **P < 0.01, *P < 0.05).

As drug resistance is a major cause of treatment failure in case of chemotherapy in clinical practice, we next assessed whether the benefit of multi-drug nanotherapy is observed in both sensitive and resistant tumour models. Although sensitive tumours were responsive to all treatments by comparison with single free drug therapy, resistant tumours only showed a statistically significant response to combination nanotherapy, reducing tumour growth by 43.9% compared with single free drug therapy (Fig. 5a). Yet, it needs to be acknowledged that the lack of statistical significance between the free drug therapy and free drug combination therapy groups can be a matter of smaller sample size. Either way, in both sensitive and resistant tumour models, combination nanotherapy outperformed free drug combination therapy (Fig. 5a). Finally, we evaluated whether therapy outcomes differ between xenograft models (that is, human tumours grown in immunodeficient mice) and allograft models (that is, mouse tumours grown in immunocompetent mice). Meta-analysis of published datasets clearly shows that multi-drug nanotherapy is most efficient in both model system set-ups, statistically outperforming all other treatment groups (Fig. 5b).

### Future directions of multi-drug cancer nanomedicine

This meta-analysis demonstrates a statistically significant benefit of combination anti-cancer nanotherapy over free drug combination therapy in pre-clinical mouse models (Fig. 2). One clear reason for this beneficial outcome is undoubtfully the ability of nanocarriers to improve the circulation time and target site accumulation of drug molecules (Supplementary Fig. 2). This benefit was consistently observed across different tumour types and experimental settings (Figs. 3–5 and Supplementary Figs. 3–9). Moreover, and arguably most importantly, our meta-analysis showcases that co-loading two different drugs within the same nanomedicine formulation is statistically superior to the co-administration of two single-drug-loaded nanomedicines (Fig. 3a,b). These findings corroborate ongoing translational efforts towards expanding the use of combinatorial nanomedicines for multi-drug delivery.

The outcome of our meta-analysis should be interpreted with care. Publication bias is a factor that should not be overlooked, as many researchers opt out from publishing negative results. In most cases, we found that combination therapy is better than monotherapy, for both free drug and for nanodrug treatments; however, we also identified four cases in which monotherapy performed better than combination therapy, and 17 cases in which combination nanotherapy was less effective than single-drug nanotherapy. Nonetheless, this issue is evident even in studies focusing on single-drug nanotherapies, where researchers may only publish positive results regarding the anti-cancer efficacy of a single-drug nanomedicine intervention. Consequently, publication bias seems to affect both the single-drug and the multi-drug nanotherapy groups equally in our comparative analysis.

When translating results from mice to humans, it is important to appreciate the considerable interspecies differences, especially in terms of nanoparticle circulation half-life in blood. Mice often have faster metabolism rates and shorter circulation times than humans. Thus, nanoparticles may be cleared faster from the blood in mice, potentially leading to discrepancies in their efficacy benefits when translating findings to human applications. Advanced studies involving non-human primates may better inform pharmacokinetic assumptions about human responses.

To move the field forward and ensure translational impact, it is important to carefully consider the cases in which multi-drug delivery is helpful and needed, versus those in which it is conceptually inappropriate. For cancer chemotherapy, several scenarios can be envisaged in which multi-drug delivery adds value. Co-delivering two different cytotoxic drugs to and into the same target cell at a synergistic ratio is viable and valuable, as evidenced by the successful product development and clinical approval of the frontrunner double-drug nanoformulation Vyxeos. This non-PEGylated liposome, which contains daunorubicin and cytarabine in a 5:1 ratio, efficiently targets and kills leukaemia cells upon intravenous administration, and it creates significant added value for patients, with an improvement in median overall survival from six months for daunorubicin and cytarabine in free form to ten months for Vyxeos (HR = 0.69; *P* = 0.003)^8^. When co-administered in free form, because of the different physicochemical and pharmacokinetic parameters of daunorubicin and cytarabine, it seems chanceless to achieve synergistic ratios in target cells, even if intravenously administered at a 5:1 dose. If two single-drug-loaded liposomes would be administered at a 5:1 dose, the question is how many of those would eventually end up in that ideal ratio in the same leukaemia target cell in the blood stream or in the bone marrow. For three-drug chemotherapy combinations, mathematical modelling has shown that chances are >10 higher to achieve synergistic drug ratios in target cells when co-formulating drugs in nanomedicines versus co-administering single-drug nanomedicines^13^. For double-drug formulations, the mathematical chances will be somewhat lower, but certainly still high enough to produce a significant and clinically meaningful increase in therapeutic outcome.

When transiting from haematological to solid cancers, the multi-drug delivery situation becomes very different. In the case of haematological malignancies, like leukaemia, liposomes have reasonably good access to leukaemia cells in the blood stream and bone marrow, particularly if they are not PEGylated (as in the case of Vyxeos). To optimally reach non-haematological solid tumours and metastases, nanomedicines are typically PEGylated, as this increases circulation times and tumour concentrations. However, PEGylation will also skew the uptake of nanoparticles away from cancer cells, towards tumour-associated macrophages. For multi-drug delivery, this means that synergistic ratios are potentially delivered to the tumour compartment as a whole, but not to individual tumour cells, where pharmacological synergy is needed the most. Nonetheless, the outcomes of our meta-analysis show that even in the case of solid tumour targeting, nanomedicine co-delivery is superior over co-administration of two single-drug-loaded nanomedicines (Fig. 3a,b). In line with the above reasoning on the importance of cancer cell uptake versus tumour-associated macrophage uptake of multi-drug-loaded nanomedicines, we found no statistically significant added value of PEGylation on therapeutic performance (Fig. 3c). Also, in agreement with this, we did find a statistically significant added value of active over passive targeting in case of nanomedicine combination therapy, which was not observed in case of active versus passive targeting with single-drug-loaded nanomedicines (Fig. 3d). It is important to keep in mind in this regard that active targeting typically only improves the balance between cancer cell versus macrophage uptake, and not the overall levels of nanomedicine tumour accumulation^40–42^. Accordingly, in the case of multi-drug nanomedicine, where transportation of synergistic amounts of chemotherapeutic drugs to and into cancer cells is crucial, active targeting is identified here as an important enabler.

The added value of multi-drug cancer nanomedicine crucially depends on the mechanism of action of the agents that are being co-delivered. When combining a classical chemotherapy drug with a tumour microenvironment-modulating or immune-activating agent, it seems unnecessary to co-formulate both agents in the same nanoparticle. There may be intellectual property-related reasons or other translationally relevant arguments for doing so (for example, having to perform toxicology studies and phase I trials for just one double-drug nanoformulation versus for two single-drug nanomedicines), but from a pure pharmacological point of view, there does not seem to be much added value in co-loading agents that do not target the same cell. Scenarios can be envisaged in which there can be benefit, for example, when co-delivering a vascular disrupting agent together with a chemotherapy drug in a nanoparticle that enables temporally controlled release kinetics, with the former agent being released first, to cause tumour vascular shutdown, and the latter agent being released afterwards, to restrict cytotoxicity effects to the tumour compartment and attenuate systemic side effects^43^.

Conceptually and scientifically, multi-drug nanomedicine therapies are elegant and appealing. The possibility of being able to co-deliver more than one active pharmaceutical ingredient to the same site or cell in the body at the same point in time opens up many therapeutic opportunities. These go way beyond cancer, encompassing for example, also the co-delivery of 3–4 antiviral drugs for HIV or tuberculosis therapy, or the co-delivery of siRNAs, miRNAs, mRNAs and/or sgRNAs for optimized protein replacement therapy, gene silencing or gene editing^11,44–46^.

To create clinical impact, it will be crucial to align scientific and conceptual nanotechnology engineering advances with rational pharmacological mechanisms and realistic clinical scenarios. The multi-drug nanomedicine field is still in its infancy, with only Vyxeos on the market, and only a few double-drug formulations in clinical development. It is expected that in the years to come, multi-drug nanomedicines will gain more traction. From a pharmaceutical technology and industrial development point of view, they are very appealing, as there still is a lot of intellectual property and clinical application space to claim. Based on the outcome of our meta-analysis, and on the above-discussed insights and opportunities, we conclude that multi-drug nanomedicine holds significant promise for applications in oncology and beyond.

## Conclusion

This meta-analysis shows that, in pre-clinical mouse models, multi-drug cancer nanomedicine therapy improves tumour growth inhibition and prolongs survival times compared with free drug combination regimens. Most importantly, our analysis exemplifies that combination nanotherapy achieves the most therapeutic benefit when two drugs are co-administered in the same formulation, rather than when applying them sequentially or simultaneously in two separate nanoformulations. The importance of these findings is confirmed for different types of drugs and in multiple pre-clinical tumour models. These insights instigate and inspire efforts to explore multi-drug nanomedicine for applications in oncology and beyond.

## Methods

### Literature search

As a first step, we scanned exemplary literature for identifying groups of keywords relevant to multi-drug nanomedicine research for oncological purposes. Through this process, we identified three relevant groups of keywords, namely, ‘combination therapy’, ‘combination chemotherapy’ and ‘co-delivery' (or 'co-delivery’); we then combined them with the term ‘nanomedicine’ to eventually perform three individual group keyword searches on scopus.com: (1) ‘combination therapy’ AND ‘nanomedicine’; (2) ‘combination chemotherapy’ AND ‘nanomedicine’; and (3) (‘co-delivery’ OR ‘co-delivery’) AND ‘nanomedicine’. The manuscripts derived upon searching for the above-mentioned terms, and published between January 2007 and December 2022, were then classified and included in the final analysis once meeting the following criteria: (1) they were a peer-reviewed research article of a primary study; (2) they contained in vivo quantitative therapy data from pre-clinical murine models; (3) they focused on oncological disorders; (4) they contained at least one pharmacologically active small drug molecule with proven anti-cancer effect; (5) they contained at least two loadable drugs, that is, cases where the nanocarrier acts as therapeutic component (for example, photothermal therapy) were not included; and (6) they presented tumour growth curves as therapy efficacy readouts (Supplementary Tables 3 and 4). Following the initial search, screening and inclusion/exclusion, the studies were then used for extraction and digitalization. Next, they were all thoroughly screened for analysis accuracy and clarity in result presentation. Data extraction included qualitative and quantitative information, for example, the drug used, the type of nanoformulation, the tumour model, the administration route, the tumour growth kinetics and so on (Supplementary Table 1).

### Tumour growth curves and plots

Tumour sizes were extracted from in vivo therapy studies that reported tumour sizes in the text or presented tumour growth curves as part of the figures. From tumour growth curves, numbers were extracted using the online plot digitizer graphreader (www.graphreader.com). Tumour sizes were usually indicated as absolute or relative tumour volumes (in millimetres-cubed), tumour volume increase (%) or tumour inhibition rate (%). These tumour sizes were compared with the control groups in the same in vivo therapy studies—mostly encompassing mice injected with PBS or vehicles. Absolute tumour values from each therapy study were then compared with the control group growth. In each individual study, the latter was normalized as ‘100%’ tumour growth, and the values of the various treatment groups were expressed as a percentage of the normalized tumour growth. The therapies injected to mice were categorized in four groups; namely, therapy (single free-drug), combination therapy (free-drug combination), nanotherapy (single-drug nanocarrier) and, finally, combination nanotherapy (multi-drug nanocarriers, combination of single-drug nanocarriers, or mixtures of free and nanodrugs). The single and combination nanotherapy categories were further subdivided depending on the focus of different analyses (for example, passive versus active targeting, nanomaterial comparison, and so on). Numbers were entered as replicate values in GraphPad Prism 9 (ref. ^47^) and graphed as violin plots for depicting the mean, range, and 25%, 50% and 75% percentiles. Statistical significance was determined by a two-sided Kruskal–Wallis test with Dunn’s correction for multiple comparisons, or a two-tailed Mann–Whitney test (****P < 0.0001, ***P < 0.001, **P < 0.01, *P < 0.05). Descriptive statistics and *P*-values are displayed in Supplementary Tables 5–55.

### Survival curves

Kaplan–Meier survival curves from mice treated with single-drug therapy, nanotherapy, combination therapy or combination nanotherapy, were extracted from publications. Curves were normalized for each study internally, taking into consideration the individual study duration, and they were rearranged to fit in a defined quadratic area. The analysis was performed using Inkscape Project (2020).

### Forest plot

For the forest plot analysis, non-normalized tumour sizes, standard deviations, and animal group size from mice treated with combination therapy (free-drug combination) versus combination nanotherapy were extracted from tumour growth curves. Values were entered into a systematic review software (Review Manager, v.5.4.1)^25^ to perform a meta-analysis using an inverse variance and random effects model. The standardized mean difference in effect between combination therapy and combination nanotherapy is expressed in numbers or depicted graphically as a forest plot. Heterogeneity between the groups was investigated using *τ*^2^ and *χ*^2^ tests, the *I*^2^ index and was visually presented via a funnel plot, displaying standard mean difference versus standard error.

## Online content

Any methods, additional references, Nature Portfolio reporting summaries, source data, extended data, supplementary information, acknowledgements, peer review information; details of author contributions and competing interests; and statements of data and code availability are available at 10.1038/s41565-025-01932-1.

## Supplementary information

Supplementary Information Supplementary Figs. 1–12, Tables 1–55 and references.

## Data Availability

All data supporting the results of this study are available within the paper and Supplementary Information.
