# Supplementary material for: Analysis of multi-drug cancer nanomedicine
Source: Nat Nanotechnol. 2025 May 15;20(8):1163–72. doi: 10.1038/s41565-025-01932-1 (PMC12373501; doi:10.1038/s41565-025-01932-1)
Supplement: Supplementary file 1 — Supplementary Figs. 1–12, Tables 1–55 and references. [file 41565_2025_1932_MOESM1_ESM.pdf]

nature nanotechnology

Supplementary information https://doi.org/10.1038/s41565-025-01932-1

Analysis of multi-drug cancer nanomedicine

In the format provided by the authors and unedited

SUPPLEMENTARY INFORMATION

- Supplementary Table 1
- Supplementary Table 2
- Supplementary Figures 1 - 12
- Supplementary Tables 3 - 55
- Supplementary References

Number	Name	Drug 1	Drug 2	Drug 3	Nanomaterial	PEGylation	Delivery type	Proven synergism	Immunological status	Cancer type	Cell line	Resistance status	Administration type	Drug administration (times)	Time span (cell inoculation until death of control group)	Death of control group (days)	Treatment period (days)	Time passed after the administration of the last treatment (days)
**Study identification**		**Used drugs**			**Nanoparticle composition**			**Tumor Model**					**Therapy study**					
**Paper 1**	Co-Administration of Tretinoin Enhances the Anti-Cancer Efficacy of Etoposide via Tumor-Targeted Green Nano-Micelles	etoposide	trans-retinoic acid	x	other	x	co-delivery	yes	allograft	ascite	Ehrlich ascites	sensitive	intravenous	6	x	20	20	0
**Paper 2**	Cuprous oxide nanoparticles trigger reactive oxygen species-induced apoptosis through activation of erk-dependent autophagy in bladder cancer	GEM	Cu2O	x	other	x	separate, sequential, free drug	yes	xenograft	bladder	T24	sensitive	intratumoral + intraperitoneal	8	x	23	21	2
**Paper 3**	Synergistic Chemotherapy for Breast Cancer and Breast Cancer Brain Metastases via Paclitaxel-Loaded Oleanolic Acid Nanoparticles	PTX	oleanolic acid	x	other	x	co-delivery	yes	xenograft	breast	MDA-MB-231	sensitive	intravenous	9	x	25	21	4
**Paper 4**	Triple drugs co-delivered by a small gemcitabine-based carrier for pancreatic cancer immunochemotherapy	GEM	PTX	NLG919 IDO 1 inhibitor	polymer	PEG	co-delivery	no	allograft	pancreas	PANC02	sensitive	intravenous	6	x	21	20	1
**Paper 4**	Triple drugs co-delivered by a small gemcitabine-based carrier for pancreatic cancer immunochemotherapy	GEM	PTX	NLG919 IDO 1 inhibitor	polymer	PEG	co-delivery	no	allograft	breast	4T1	sensitive	intravenous	6	x	21	20	1
**Paper 4**	Triple drugs co-delivered by a small gemcitabine-based carrier for pancreatic cancer immunochemotherapy	GEM	PTX	NLG919 IDO 1 inhibitor	polymer	PEG	co-delivery	no	allograft	colon	CT26	sensitive	intravenous	6	x	18	15	3
**Paper 5**	Stimuli-responsive polymeric prodrug-based nanomedicine delivering nifuroxazide and doxorubicin against primary breast cancer and pulmonary metastasis	DOX	nifuroxazide	x	polymer	PEG	co-delivery	no	allograft	breast	4T1	sensitive	intravenous	5	30	20	15	5
**Paper 6**	Combination therapy of lung cancer using layer-by-layer cisplatin prodrug and curcumin co-encapsulated nanomedicine	cisplatin	curcumin	x	lipid-polymer	x	co-delivery	no	xenograft	lung	A549	sensitive	intravenous	5	x	21	16	5
**Paper 7**	Co-delivery of doxorubicin and paclitaxel by reduction/pH dual responsive nanocarriers for osteosarcoma therapy	DOX	PTX	x	polymer	PEG	co-delivery	yes	allograft	osteosarcoma	K7	sensitive	intravenous	4	x	16	9	7
**Paper 8**	Hyaluronic acid reduction-sensitive polymeric micelles achieving co-delivery of tumor-targeting paclitaxel/apatinib effectively reverse cancer multidrug resistance	PTX	apatinib	x	polymer	nonPEG	co-delivery	yes	xenograft	breast	MCF-7/MDR	resistant	intravenous	3	x	21	6	15
**Paper 9**	Actively priming autophagic cell death with novel transferrin receptor-targeted nanomedicine for synergistic chemotherapy against breast cancer	PTX	rapamycin	x	lipid-polymer	x	separate, sequential	yes	xenograft	breast	MCF-7	sensitive	intravenous	8	x	15	10	5
**Paper 10**	Self-assembled green tea polyphenol-based coordination nanomaterials to improve chemotherapy efficacy by inhibition of carbonyl reductase 1	DOX	Epigallocatechingallate	x	polymer	PEG	co-delivery	no	xenograft	brain	U87MG	sensitive	intravenous	3	x	24	4	20
**Paper 11**	Small Molecular Theranostic Assemblies Functionalized by Doxorubicin-Hyaluronic Acid-Methotrexate Prodrug for Multiple Tumor Targeting and Imaging-Guided Combined Chemo-Photothermal Therapy	DOX	ICG	x	polymer	nonPEG	co-delivery	no	allograft	breast	4T1	sensitive	intravenous	1	x	13	0	13
**Paper 12**	Preparation of a one-dimensional nanorod/metal organic framework Janus nanoplatform: Via side-specific growth for synergistic cancer therapy	DOX	Au	x	inorganic	x	co-delivery	no	allograft	liver	H22	sensitive	intravenous	1	x	10	1	9
**Paper 13**	A nano-complex system to overcome antagonistic photo-chemo combination cancer therapy	DOX	Ce6	x	polymer	PEG	co-delivery	yes	xenograft	Fibrosarcoma	UV-2237M/MDR	resistant	intravenous	1	x	12	1	11
**Paper 14**	Polyphenol-based nanoplatform for MRI/PET dual-modality imaging guided effective combination chemotherapy	DOX	simvastatin	x	polymer	PEG	co-delivery	no	xenograft	brain	U87MG	sensitive	intravenous	3	x	14	4	10
**Paper 15**	A polymeric nanogel-based treatment regimen for enhanced efficacy and sequential administration of synergistic drug combination in pancreatic cancer	cisplatin	GEM	x	polymer	PEG	separate	no	xenograft	pancreas	T3M4	sensitive	intravenous	4	28	14	12	2
**Paper 16**	Assembly simulation and synergistic chemotherapy of TPGS derivative functionalized polymersomes in hepatocellular carcinoma	DOX	oxaliplatin	x	polymer	PEG	co-delivery	yes	xenograft	liver	HepG2/MDR	resistant	intravenous	4	x	21	9	12
**Paper 17**	Dual-functionalized liposome by co-delivery of paclitaxel with sorafenib for synergistic antitumor efficacy and reversion of multidrug resistance	PTX	sorafenib	x	lipid	nonPEG	co-delivery	no	xenograft	breast	MCF-7/MDR	resistant	intravenous	5	x	9	8	1
**Paper 18**	Magnetic field-inducible drug-eluting nanoparticles for image-guided thermo-chemotherapy	DOX	iron oxide	x	polymer	nonPEG	co-delivery	no	allograft	colon	CT26	sensitive	intratumoral	2	x	14	3	11
**Paper 19**	Methotrexate-based amphiphilic prodrug nanoaggregates for co-administration of multiple therapeutics and synergistic cancer therapy	DOX	CPT	MTX	other	x	co-delivery / separate	yes	xenograft	breast	MCF-7	sensitive	intravenous	6	x	24	20	4
**Paper 20**	Charge-reversal-functionalized PLGA nanobubbles as theranostic agents for ultrasonic-imaging-guided combination therapy	DOX	P-gp ShRNA	x	other	x	co-delivery	no	xenograft	breast	MCF-7/MDR	resistant	intratumoral	3	16	10	9	1
**Paper 21**	Oral administration of edelfosine encapsulated lipid nanoparticles causes regression of lung metastases in pre-clinical models of osteosarcoma	DOX	edelfosine	x	lipid	nonPEG	separate, sequential, free drug	no	xenograft	osteosarcoma	HOS	sensitive	oral + intravenous	12	27	21	20	1
**Paper 22**	TGF-β inhibition combined with cytotoxic nanomedicine normalizes triple negative breast cancer microenvironment towards anti-tumor immunity	DOX	tranilast	x	lipid	PEG	separate, sequential, free drug	no	allograft	breast	4T1	sensitive	oral + intraperitoneal / intravenous	22	24	20	20	0
**Paper 22**	TGF-β inhibition combined with cytotoxic nanomedicine normalizes triple negative breast cancer microenvironment towards anti-tumor immunity	DOX	tranilast	antiCTLA-4/anti-PD-1	lipid	PEG	separate, sequential, free drug	no	allograft	breast	4T1	sensitive	oral + intraperitoneal / intravenous	25	24	20	20	0
**Paper 22**	TGF-β inhibition combined with cytotoxic nanomedicine normalizes triple negative breast cancer microenvironment towards anti-tumor immunity	DOX	tranilast	x	lipid	PEG	separate, sequential, free drug	no	allograft	breast	E0771	sensitive	oral + intraperitoneal / intravenous	22	26	22	22	0
**Paper 22**	TGF-β inhibition combined with cytotoxic nanomedicine normalizes triple negative breast cancer microenvironment towards anti-tumor immunity	DOX	tranilast	antiCTLA-4/anti-PD-1	lipid	PEG	separate, sequential, free drug	no	allograft	breast	E0771	sensitive	oral + intraperitoneal / intravenous	25	26	22	22	0
**Paper 23**	Nanostructured Lipid Carrier Co-loaded with Doxorubicin and Docosahexaenoic Acid as a Theranostic Agent: Evaluation of Biodistribution and Antitumor Activity in Experimental Model	DOX	docosahexaenoic acid	x	lipid	PEG	co-delivery	no	allograft	breast	4T1	sensitive	intravenous	4	17	10	6	4
**Paper 24**	Liposomal Codelivery of Doxorubicin and Andrographolide Inhibits Breast Cancer Growth and Metastasis	DOX	andrographolide	x	lipid	PEG	co-delivery	no	allograft	breast	4T1	sensitive	intravenous	9	x	17	16	1
**Paper 25**	Selective mTORC2 inhibitor therapeutically blocks breast cancer cell growth and survival	lapatinib	Rictor siRNA	x	polymer	PEG	separate, sequential, free drug	no	xenograft	breast	MDA-MB-361	sensitive	oral + intravenous	25	x	20	19	1
**Paper 26**	P-glycoprotein-targeted photodynamic therapy boosts cancer nanomedicine by priming tumor microenvironment	DOX	IR700	x	other	x	separate, sequential	yes	xenograft	cervix	KB-8-5-11 +3T3, MDR	resistant	intravenous	1	14	7	2	5
**Paper 27**	Gastric cancer combination therapy: Synthesis of a hyaluronic acid and cisplatin containing lipid prodrug coloaded with sorafenib in a nanoparticulate system to exhibit enhanced anticancer efficacy and reduced toxicity	cisplatin	sorafenib	x	lipid	PEG	co-delivery	no	xenograft	gastric	MKN28	sensitive	intravenous	5	x	21	12	9
**Paper 28**	The effects of novel chitosan-targeted gemcitabine nanomedicine mediating cisplatin on epithelial mesenchymal transition, invasion and metastasis of pancreatic cancer cells	GEM	cisplatin	x	polymer	nonPEG	separate	no	xenograft	pancreas	SW1990	sensitive	intravenous	4	x	24	21	3
**Paper 29**	Drug-induced co-assembly of albumin/catalase as smart nano-theranostics for deep intra-tumoral penetration, hypoxia relieve, and synergistic combination therapy	PTX	catalase	Ce6	other	x	co-delivery	no	allograft	breast	4T1	sensitive	intravenous	1	x	14	1	13
**Paper 30**	Sonic-hedgehog pathway inhibition normalizes desmoplastic tumor microenvironment to improve chemo- and nanotherapy	DOX	vismodegib	x	lipid	PEG	separate, sequential	no	xenograft	breast	MCF10CA1a	sensitive	oral+intravenous	26	28	24	24	0
**Paper 30**	Sonic-hedgehog pathway inhibition normalizes desmoplastic tumor microenvironment to improve chemo- and nanotherapy	PTX	vismodegib	x	other	x	separate, sequential, free drug	no	xenograft	breast	MCF10CA1a	sensitive	oral+intravenous	26	28	24	24	0

Number	Normalized Tumor Sizes																								
													Studies using two or three drugs								Additional categorization of two-drug combinations in studies using three drugs					
	Control	Therapy			Combination therapy		Passive nanotherapy			Active nanotherapy			Passive combination nanotherapy				Active combination nanotherapy			Passive combination nanotherapy (two drugs)				Active combination nanotherapy (two drugs)	
													Co-delivery		Separate delivery		Co-delivery	Separate delivery		Co-delivery			Separate delivery	Co-delivery	
		Drug 1	Drug 2	Drug 3	Two drugs	Three drugs	Drug 1	Drug 2	Drug 3	Drug 1	Drug 2	Drug 3	Full combination (1+2 for two-drug studies, 1+2+3 for three-drug studies)	Full combination (1+2 for two-drug studies, 1+2+3 for three-drug studies)	Full combination (1+2 for two-drug studies, 1+2+3 for three-drug studies)	Full combination (1+2 for two-drug studies, 1+2+3 for three-drug studies)	Full combination (1+2 for two-drug studies, 1+2+3 for three-drug studies)	Full combination (1+2 for two-drug studies, 1+2+3 for three-drug studies)	Full combination (1+2 for two-drug studies, 1+2+3 for three-drug studies)	Drug 1+2	Drug 1+3	Drug 2+3	Drug 1+2	Drug 1+2	Drug 2+3
**Paper 1**	100.0	35.9	31.0		25.7								14.3												
**Paper 2**	100.0	12.2						40.8							4.1										
**Paper 3**	100.0	85.0	86.0										31.0												
**Paper 4**	100.0					50.0	63.6						20.5							40.9	34.1				
**Paper 4**	100.0					45.5	63.6						9.1							31.8	22.7				
**Paper 4**	100.0					63.0	22.2						9.3							16.7	22.2				
**Paper 5**	100.0	79.4	90.6				73.0						43.1												
**Paper 6**	100.0	82.2	93.7		76.9		75.4	83.9					23.3												
**Paper 7**	100.0	70.0	78.0				22.0	65.0					13.0												
**Paper 8**	100.0	72.9			51.8					33.3							14.5								
**Paper 9**	100.0				25.1					31.1	65.5					18.7			9.4						
**Paper 10**	100.0	59.9	82.0		55.9								30.0												
**Paper 11**	100.0	45.5			9.1					79.5							4.5								
**Paper 12**	100.0	46.9								26.9	62.5						3.1								
**Paper 13**	100.0						70.8	54.2					25.8	8.3											
**Paper 14**	100.0	46.7	66.7		36.7								6.7												
**Paper 15**	100.0		51.9				76.3			57.7					37.8			14.4							
**Paper 16**	100.0	60.9	57.8		28.3								29.7				6.5								
**Paper 17**	100.0	50.0	69.2		34.6								28.8				22.3								
**Paper 18**	100.0						81.4	61.0					5.8												
**Paper 19**	100.0															34.0					60.0	57.0			
**Paper 20**	100.0	68.0					56.0						35.0												
**Paper 21**	100.0	74.6	27.6					50.7							82.8										
**Paper 22**	100.0		98.2				74.0								34.1										
**Paper 22**	100.0			90.7											20.0										
**Paper 22**	100.0		102.7				91.8								30.1										
**Paper 22**	100.0			89.1											18.2										
**Paper 23**	100.0	43.8					31.6						9.7												
**Paper 24**	100.0				63.3								39.8				19.9								
**Paper 25**	100.0	47.6						31.7							11.1										
**Paper 26**	100.0						54.2				58.3								16.7						
**Paper 27**	100.0	71.8	65.3		55.2					32.6	39.2		26.9				17.0								
**Paper 28**	100.0						83.3			50.0								40.0							
**Paper 29**	100.0												16.3							73.2	32.5	48.8			
**Paper 30**	100.0		99.3				91.5								15.5										
**Paper 30**	100.0		99.3				95.8								42.3										

**Paper 31**	Panitumumab-Conjugated Pt-Drug Nanomedicine for Enhanced Efficacy of Combination Targeted Chemotherapy against Colorectal Cancer	oxaliplatin	panitumumab	x	other	x	co-delivery	no	xenograft	colon	Caco2	sensitive	intravenous	3	x	35	8	27	100.0	99.0	99.0		69.0								7.0												
**Paper 31**	Panitumumab-Conjugated Pt-Drug Nanomedicine for Enhanced Efficacy of Combination Targeted Chemotherapy against Colorectal Cancer	oxaliplatin	panitumumab	x	other	x	co-delivery	no	xenograft	colon	HT29	sensitive	intravenous	3	x	35	8	27	100.0	100.0	87.5		79.6								20.0												
**Paper 32**	MiR-375 and Doxorubicin Co-delivered by Liposomes for Combination Therapy of Hepatocellular Carcinoma	DOX	miRNA-375	x	lipid	PEG	co-delivery	no	xenograft	liver	HepG2	sensitive	intravenous	3	x	13	9	4	100.0	27.5					23.2	71.5					7.4												
**Paper 33**	Programmed Multiresponsive Vesicles for Enhanced Tumor Penetration and Combination Therapy of Triple-Negative Breast Cancer	DOX	oxaliplatin	pyropheophorbide-a	lipid	PEG	co-delivery	yes	allograft	breast	4T1	sensitive	intravenous	2	x	13	7	6	100.0						43.5	39.1	5.8				1.4							20.3					
**Paper 34**	IONP-doped nanoparticles for highly effective nir-controlled drug release and combination tumor therapy	DOX	carbon nanotubes	x	lipid	PEG	co-delivery	no	allograft	breast	4T1	sensitive	intravenous	2	19	13	13	0	100.0	52.8					93.1			81.9	54.2						6.3								
**Paper 35**	Efficient co-delivery of immiscible hydrophilic/hydrophobic chemotherapeutics by lipid emulsions for improved treatment of cancer	oxaliplatin	irinotecan	x	lipid	nonPEG	co-delivery / separate	yes	allograft	colon	CT26	sensitive	intravenous	3	28	21	14	7	100.0	63.2	80.7		49.1		75.4	71.9					33.3			42.1									
**Paper 36**	Development of a hybrid paclitaxel-loaded arsenite nanoparticle (HPAN) delivery system for synergistic combined therapy of paclitaxel-resistant cancer	PTX	arsenic trioxide	x	inorganic	x	co-delivery	no	xenograft	colon	HCT 116/MDR	resistant	intravenous	8	x	20	14	6	100.0	56.4			69.1								21.8												
**Paper 37**	Visible light-induced crosslinking and physiological stabilization of diselenide-rich nanoparticles for redox-responsive drug release and combination chemotherapy	DOX	CPT	x	polymer	PEG	co-delivery	no	allograft	breast	EMT-6	sensitive	intravenous	6	31	24	20	4	100.0	60.0	53.6										8.6												
**Paper 38**	Supramolecular “Trojan Horse” for Nuclear Delivery of Dual Anticancer Drugs	cisplatin	HCPT	x	other	x	co-delivery	yes	allograft	breast	4T1	sensitive	intravenous	5	x	21	12	9	100.0	84.2	82.2										19.8												
**Paper 39**	Lymph cancer chemotherapy: Delivery of doxorubicin-gemcitabine prodrug and vincristine by nanostructured lipid carriers	DOX	GEM	vincristine	lipid	nonPEG	co-delivery	no	xenograft	lymph	Raji	sensitive	intravenous	5	x	21	12	9	100.0			73.9	67.0	62.6			28.7				13.8							21.8					
**Paper 40**	Epothilone B-based 3-in-1 polymeric micelle for anticancer drug therapy	epothilone B	rapamycin	17-AAG	polymer	PEG	co-delivery	yes	xenograft	lung	A549	sensitive	intravenous	4	x	35	21	14	100.0	86.8											0.0												
**Paper 41**	Molecularly targeted co-delivery of a histone deacetylase inhibitor and paclitaxel by lipid-protein hybrid nanoparticles for synergistic combinational chemotherapy	PTX	vorinostat	x	other	x	co-delivery	yes	xenograft	liver	HepG2	sensitive	intravenous	4	42	28	9	19	100.0	63.6	77.3		52.2								33.0				20.4								
**Paper 42**	Doxorubicin- and cisplatin-loaded nanostructured lipid carriers for breast cancer combination chemotherapy	DOX	cisplatin	x	lipid	nonPEG	co-delivery	no	xenograft	breast	MCF-7/MDR	resistant	intravenous	7	x	21	18	3	100.0	80.2	61.1				48.1	38.1					16.0												
**Paper 43**	Targeted hepatocellular carcinoma therapy: transferrin modified, self-assembled polymeric nanomedicine for co-delivery of cisplatin and doxorubicin	DOX	cisplatin	x	polymer	PEG	co-delivery	yes	xenograft	liver	HepG2	sensitive	intravenous	4	x	24	21	3	100.0	57.3	72.0		53.9			51.2		36.0							19.8								
**Paper 44**	Rationally Designed 2-in-1 Nanoparticles Can Overcome Adaptive Resistance in Cancer	DTX	dasatanib	PI3K inhibitor SPI103	lipid	PEG	co-delivery / free drug	yes	allograft	breast	4T1	sensitive	intravenous	3	x	16	13	3	100.0	53.8											76.9		30.8										
**Paper 45**	Co-delivery of baicalein and doxorubicin by hyaluronic acid decorated nanostructured lipid carriers for breast cancer therapy	DOX	baicalein	x	lipid	nonPEG	co-delivery	yes	xenograft	breast	MCF-7/MDR	resistant	intravenous	3	x	21	14	7	100.0				51.6		82.2	28.0					17.9				11.5								
**Paper 46**	A nanomedicine based combination therapy based on QLPVM peptide functionalized liposomal tamoxifen and doxorubicin against Luminal A breast cancer	DOX	tamoxifen	x	lipid	PEG	separate, sequential	yes	xenograft	breast	MCF-7	sensitive	intravenous	4	27	21	11	10	100.0	48.6	87.1		42.7		47.2	65.7		25.7	50.6					38.2			13.5						
**Paper 47**	Polymeric Nanomedicine for Combined Gene/Chemotherapy Elicits Enhanced Tumor Suppression	PTX	PEDF DANN	x	polymer	PEG	co-delivery / separate	no	allograft	colon	CT26	sensitive	intravenous	8	x	14	14	0	100.0	56.3					37.5	35.0					23.8			33.6									
**Paper 48**	Active tumor permeation and uptake of surface charge-switchable theranostic nanoparticles for imaging-guided photothermal/chemo combinatorial therapy	DOX	ICG	x	polymer	nonPEG	co-delivery	no	allograft	prostate	TRAMP-C1	sensitive	intravenous	1	x	15	0	15	100.0	96.1	87.2										67.8	24.0											
**Paper 49**	Engineering of lipid prodrug-based, hyaluronic acid-decorated nanostructured lipid carriers platform for 5-fluorouracil and cisplatin combination gastric cancer therapy	5-FU	cisplatin	x	lipid	nonPEG	co-delivery	yes	xenograft	gastric	BGC823	sensitive	intravenous	3	x	21	14	7	100.0	75.8	71.7		54.5		49.5	46.5					43.4				23.2								
**Paper 50**	Delivery of baicalein and paclitaxel using self-assembled nanoparticles: Synergistic antitumor effect in vitro and in vivo	PTX	baicalein	x	polymer	nonPEG	co-delivery	yes	xenograft	lung	A549/MDR	resistant	intravenous	7	x	24	24	0	100.0	91.1	78.6		62.5					64.3	60.7						33.9								
**Paper 51**	Co-delivery of all-trans-retinoic acid and doxorubicin for cancer therapy with synergistic inhibition of cancer stem cells	DOX	trans-retinoic acid	x	polymer	PEG	co-delivery / separate	no	xenograft	breast	MDA-MB-231	sensitive	intravenous	11	48	20	11	9	100.0	66.7	75.0		45.8		56.3	66.3					16.7			25.0									
**Paper 52**	Safety and efficacy of quadrapeutics versus chemoradiation in head and neck carcinoma xenograft model	cisplatin	panitumumab	Au	other	PEG	separate	no	xenograft	head and neck	HN31	sensitive	intravenous	x	x	18	7	11	100.0	17.2														3.8				17.2					
**Paper 52**	Safety and efficacy of quadrapeutics versus chemoradiation in head and neck carcinoma xenograft model	DOX	panitumumab	Au	other	PEG	separate	no	xenograft	head and neck	HN31	sensitive	intravenous	x	x	18	x	x	100.0															0.4									
**Paper 53**	Dual pH-sensitive oxidative stress generating micellar nanoparticles as a novel anticancer therapeutic agent	benzoyloxy cinnamaldehyde	zinc porphyrin	x	polymer	nonPEG	co-delivery	no	xenograft	colon	SW620	sensitive	intravenous	8	x	22	21	1	100.0	53.3	48.9		46.7		35.6						20.0												
**Paper 54**	Modulating cancer multidrug resistance by sertraline in combination with a nanomedicine	DOX	sertraline	x	lipid	PEG	separate, sequential	no	xenograft	ovarian	OVCAR-8	sensitive	oral+intravenous	33	x	45	33	12	100.0		87.3				68.3								36.5										
**Paper 55**	A smart and versatile theranostic nanomedicine platform based on nano porphyrin	DOX	pyropheophorbide-a	x	polymer	PEG	co-delivery	no	xenograft	ovarian	SKOV3	sensitive	intravenous	3	x	16	9	7	100.0	54.9					53.5	52.2					19.2												
**Paper 56**	Doxorubicin and lapatinib combination nanomedicine for treating resistant breast cancer	DOX	lapatinib	x	polymer	PEG	separate	no	xenograft	breast	MCF-7/MDR	resistant	intravenous	x	x	21	21	0	100.0	20.0					17.8									5.6									
**Paper 57**	Codelivery of doxorubicin and paclitaxel by cross-linked multilamellar liposome enables synergistic antitumor activity	DOX	PTX	x	lipid	PEG	co-delivery	yes	allograft	breast	4T1	sensitive	intravenous	8	37	21	21	0	100.0				68.3								44.1												
**Paper 57**	Codelivery of doxorubicin and paclitaxel by cross-linked multilamellar liposome enables synergistic antitumor activity	DOX	PTX	x	lipid	PEG	co-delivery	yes	allograft	breast	4T1	sensitive	intravenous	8	37	21	21	0	100.0				75.0								21.6												
**Paper 57**	Codelivery of doxorubicin and paclitaxel by cross-linked multilamellar liposome enables synergistic antitumor activity	DOX	PTX	x	lipid	PEG	co-delivery	yes	allograft	breast	4T1	sensitive	intravenous	8	37	21	21	0	100.0				65.0								62.5												
**Paper 58**	Encapsulation of poorly soluble drugs in polymer-drug conjugates: Effect of dual-drug nanoformulations on cancer therapy	PTX	floxuridine	x	polymer	nonPEG	co-delivery	no	allograft	breast	4T1	sensitive	oral	4	20	12	x	x	100.0							77.4					63.4												
**Paper 58**	Encapsulation of poorly soluble drugs in polymer-drug conjugates: Effect of dual-drug nanoformulations on cancer therapy	PTX	17-AAG	x	polymer	nonPEG	co-delivery	no	allograft	breast	4T1	sensitive	oral	4	20	12	x	x	100.0							77.4					53.5												
**Paper 59**	Intrinsically active nanobody-modified polymeric micelles for tumor-targeted combination therapy	DOX	EGa1	x	polymer	PEG	co-delivery	no	xenograft	head and neck	14C	sensitive	intravenous	5	x	17	12	5	100.0	60.0					42.9	76.2					27.1												
**Paper 59**	Intrinsically active nanobody-modified polymeric micelles for tumor-targeted combination therapy	DOX	EGa1	x	polymer	PEG	co-delivery	no	xenograft	head and neck	14C	sensitive	intravenous	4	x	17	9	8	100.0						24.8						23.8												
**Paper 60**	Combination of TRAIL and Actinomycin D liposomes enhances antitumor effect in non-small cell lung cancer	actinomycin D	TRAIL	x	lipid	PEG	separate	no	xenograft	lung	A549	sensitive	intravenous	12	39	19	12	7	100.0	89.8	100.0		81.4		84.9	100.0								49.1									
**Paper 61**	Multifunctional magnetic Fe3O4 nanoparticles combined with chemotherapy and hyperthermia to overcome multidrug resistance	daunorubicin	5-bromotetrandrine	iron oxide	inorganic	x	co-delivery	no	xenograft	leukemia	K562/A02 (MDR)	resistant	intravenous	1	x	12	0	12	100.0	92.5		6.8	76.3								2.2												
**Paper 62**	Tumor priming enhances delivery and efficacy of nanomedicines	DOX	PTX	x	lipid	PEG	separate, sequential	no	xenograft	pharynx	FaDu	sensitive	intravenous	x	x	36	0	36	100.0		22.2		3.9										0.9										
**Paper 63**	Surfactant-stripped J-aggregates of azaBODIPY derivatives: All-in-one phototheranostics in the second near infrared window	PTX	BODIPY	x	other	x	co-delivery	no	allograft	breast	4T1	sensitive	intravenous	1	x	12	1	11	100.0						37.5	33.3					0.0												
**Paper 64**	Development of an ultrasound triggered nanomedicine-microbubble complex for chemo-photodynamic-gene therapy	DOX	Ce6	VEGF siRNa	other	x	co-delivery/separate	no	allograft	abdomen	Scc-7	sensitive	intravenous	1	x	14	0	14	100.0						77.8									27.8				77.8					

**Paper 65**	Co-delivery of doxorubicin and DNAzyme using ZnO@polydopamine core-shell nanocomposites for chemo/gene/photothermal therapy	DOX	PDA	DNAzyme	inorganic	x	co-delivery	no	xenograft	lung	A549	sensitive	intravenous	8	x	14	14	0	100.0	23.3					65.1						23.3							39.5	41.9				
**Paper 66**	Mitochondrion- and nucleus-acting polymeric nanoagents for chemo-photothermal combination therapy [作用于线粒体和细胞核的聚合物纳米材料及其在化疗/光热治疗联合抗癌中的应用]	HCPT	IR825	x	lipid-polymer	PEG	co-delivery	no	allograft	breast	4T1	sensitive	intravenous	1	x	14	0	14	100.0	70.8	41.7							37.5	19.2						4.2								
**Paper 67**	Synergistic action of doxorubicin and 7-Ethyl-10-hydroxycamptothecin polyphosphorylcholine polymer prodrug	DOX	SN38	x	polymer	PEG	co-delivery	yes	allograft	breast	4T1	sensitive	intravenous	5	x	21	12	9	100.0	27.2					47.2	31.7					22.2												
**Paper 68**	Near-infrared/pH dual-responsive nanocomplexes for targeted imaging and chemo/gene/photothermal tri-therapies of non-small cell lung cancer	erlotinib	Cy7	survivin shRNA expressing plasmid	polymer	nonPEG	co-delivery	no	xenograft	lung	H1975	sensitive	intravenous	7	x	18	18	0	100.0								77.1				7.1								52.9	21.4			
**Paper 69**	Hyaluronic Acid-Decorated Glycol Chitosan Nanoparticles for pH-Sensitive Controlled Release of Doxorubicin and Celecoxib in Nonsmall Cell Lung Cancer	DOX	celecoxib	x	polymer	nonPEG	co-delivery	no	xenograft	lung	A549	sensitive	intravenous	4	34	20	9	11	100.0	64.5			55.9					32.5							16.3								
**Paper 70**	Size-Transformable Hyaluronan Stacked Self-Assembling Peptide Nanoparticles for Improved Transcellular Tumor Penetration and Photo-Chemo Combination Therapy	SN38	ICG	x	polymer	x	co-delivery	no	allograft	skin	B16	sensitive	intravenous	1	20	12	0	12	100.0	70.0	70.0				22.5			11.5			3.6				0.0								
**Paper 71**	Nanoformulation of Talazoparib Increases Maximum Tolerated Doses in Combination With Temozolomide for Treatment of Ewing Sarcoma	temozolomide	talazoparib	x	lipid	PEG	separate, sequential	no	xenograft	ewings sarkom	TC-71 ES	sensitive	intravenous + oral	8	x	12	5	7	100.0	71.4						92.9							28.6										
**Paper 72**	Combination of chemotherapy and photodynamic therapy for cancer treatment with sonoporation effects	DOX	Ce6	x	other	x	co-delivery	no	xenograft	pancreas	MIA-paca-2	sensitive	intravenous	1	x	14	x	x	100.0						28.6	37.1					10.3												
**Paper 73**	Near-infrared light switching nitric oxide nano emitter for triple-combination therapy of multidrug resistant cancer	DOX	BNN6	Photoresponsive	polymer	PEG	separate	no	xenograft	breast	MCF-7/MDR	resistant	intravenous	3	x	18	4	14	100.0	93.9								70.7									36.6						
**Paper 74**	Multifunctional Theranostic Liposomes Loaded with a Hypoxia-Activated Prodrug for Cascade-Activated Tumor Selective Combination Therapy	tirapazamine	ICG	Ce6	lipid	PEG	co-delivery	no	xenograft	lung	A549	sensitive	intravenous	1	x	13	0	13	100.0									91.3							8.7							39.1	17.4
**Paper 75**	Magnetic Manganese Oxide Sweetgum-Ball Nanospheres with Large Mesopores Regulate Tumor Microenvironments for Enhanced Tumor Nanotheranostics	DOX	Ce6	MnO2	inorganic	x	co-delivery	no	xenograft	brain	U87MG	sensitive	intravenous	1	x	14	0	14	100.0	78.1			60.1		45.3			53.6	24.0		3.4				1.5								
**Paper 76**	Tandem activated photodynamic and chemotherapy: Using pH-Sensitive nano systems to realize different tumor distributions of photosensitizer/prodrug for amplified combination therapy	banoxantrone	perfluorononanesulfonic acid	x	inorganic	x	co-delivery	no	xenograft	liver	HepG2	sensitive	intravenous	1	x	20	0	20	100.0						66.4	59.3					26.5												
**Paper 77**	Dual Inhibitors-Loaded Nanotherapeutics that Target Kinase Signaling Pathways Synergize with Immune Checkpoint Inhibitor	PI3K inhibitor PI103	selumetinib	x	lipid	PEG	co-delivery	yes	allograft	skin	D4M	sensitive	intravenous	3	10	12	4	8	100.0				70.5								50.0												
**Paper 77**	Dual Inhibitors-Loaded Nanotherapeutics that Target Kinase Signaling Pathways Synergize with Immune Checkpoint Inhibitor	PI3K inhibitor PI103	selumetinib	PD-L1	lipid	PEG	separate	no	allograft	skin	D4M	sensitive	intravenous	3	10	12	4	8	100.0			110.3		63.6									13.6										
**Paper 78**	A Simple Glutathione-Responsive Turn-On Theranostic Nanoparticle for Dual-Modal Imaging and Chemo-Photothermal Combination Therapy	PTX	1,1- Dioctadecyl-3,3,3,3-tetramethylindotricarbocyanine iodide	x	polymer	nonPEG	co-delivery	no	allograft	breast	4T1	sensitive	intravenous	4	x	16	4	12	100.0		94.3				89.5						19.1												
**Paper 79**	NIR-Responsive Polypeptide Nanocomposite Generates NO Gas, Mild Photothermia, and Chemotherapy to Reverse Multidrug-Resistant Cancer	DOX	NO	PDA	polymer	PEG	co-delivery	yes	xenograft	breast	MCF-7/MDR	resistant	intravenous	2	x	30	5	25	100.0	93.7					76.2						0.0								35.0	56.2			
**Paper 80**	Cancer-Selective Bio reductive Chemotherapy Mediated by Dual Hypoxia-Responsive Nanomedicine upon Photodynamic Therapy-Induced Hypoxia Aggravation	tirapazamine	Ce6	x	polymer	nonPEG	co-delivery	no	allograft	breast	4T1	sensitive	intratumoral	3	x	12	8	4	100.0	77.0								63.6	53.5						13.4								
**Paper 81**	Highly stable near-infrared dye conjugated cerasomes for fluorescence imaging-guided synergistic chemo-photothermal therapy of colorectal cancer	DOX	Cy7	x	other	x	co-delivery	no	xenograft	colon	HT29	sensitive	intravenous	1	x	15	0	15	100.0	62.1					34.5	12.4					5.0												
**Paper 82**	Toxic Reactive Oxygen Species Enhanced Synergistic Combination Therapy by Self-Assembled Metal-Phenolic Network Nanoparticles	DOX	cisplatin	x	polymer	PEG	co-delivery	yes	xenograft	brain	U87MG	sensitive	intravenous	3	x	25	4	21	100.0	40.7	44.4		77.8								15.6												
**Paper 83**	Temporary suppression the sequestrated function of host macrophages for better nanoparticles tumor delivery	PTX	clodronate	x	other	x	separate, sequential	no	allograft	skin	B16F10	sensitive	intravenous	6	x	12	7	5	100.0						37.1	68.6								12.0									
**Paper 84**	Self-Assembled Oxaliplatin(IV) Prodrug-Porphyrin Conjugate for Combinational Photodynamic Therapy and Chemotherapy	Oxaliplatin	porphyrin	x	other	x	co-delivery	no	allograft	breast	4T1	sensitive	intratumoral	2	x	14	3	11	100.0						86.9	91.0					42.8												
**Paper 85**	Water-Responsive Hybrid Nanoparticles Codelivering ICG and DOX Effectively Treat Breast Cancer via Hyperthermia-aided DOX Functionality and Drug Penetration	DOX	ICG	x	inorganic	x	co-delivery	yes	allograft	breast	4T1	sensitive	intravenous	5	x	20	8	12	100.0	58.0					36.3	43.5					8.7												
**Paper 86**	Prussian blue-modified ferritin nanoparticles for effective tumor chemo-photothermal combination therapy via enhancing reactive oxygen species production	GEM	Prussian blue	x	other	x	separate, sequential	no	allograft	breast	4T1	sensitive	intratumoral + intraperitoneal	x	x	30	x	x	100.0	74.8						86.4							0.0										
**Paper 87**	A switchable NO-releasing nanomedicine for enhanced cancer therapy and inhibition of metastasis	PTX	NO	IR789	other	x	co-delivery	no	allograft	breast	4T1	sensitive	intravenous	1	x	15	2	13	100.0						71.8						6.7							47.8					
**Paper 88**	Effective co-encapsulation of doxorubicin and irinotecan for synergistic therapy using liposomes prepared with triethylammonium sucrose octasulfate as drug trapping agent	DOX	Irinotecan	x	lipid	PEG	co-delivery / separate	yes	allograft	breast	4T1	sensitive	intravenous	3	x	14	6	8	100.0				67.9								31.4			46.4									
**Paper 89**	Ultrasound-triggered spatiotemporal delivery of topotecan and curcumin as combination therapy for cancer	topotecan	curcumin	x	other	x	co-delivery	yes	allograft	skin	B16F10	sensitive	intravenous	5	29	14	12	2	100.0				80.0		14.0	42.0					0.0												
**Paper 90**	Glucose-responsive mesoporous silica nanoparticles to generation of hydrogen peroxide for synergistic cancer starvation and chemistry therapy	PTX	glucose oxidase	x	inorganic	x	co-delivery	no	xenograft	liver	Heps	sensitive	intravenous	7	x	13	12	1	100.0	56.8					52.3						18.2				0.0								
**Paper 91**	Improving Drug Delivery of Micellar Paclitaxel against Non-Small Cell Lung Cancer by Coloading Itraconazole as a Micelle Stabilizer and a Tumor Vascular Manipulator	PTX	itraconazole	x	polymer	PEG	co-delivery / separate	no	xenograft	lung	A549/MDR	resistant	intravenous	4	25	14	9	5	100.0						75.0	70.0					20.0			70.0									
**Paper 92**	A highly integrated precision nanomedicine strategy to target esophageal squamous cell cancer molecularly and physically	DTX	AZD8186	x	polymer	x	separate	yes	xenograft	esophagus	KYSE70	sensitive	intravenous	8	x	21	21	0	100.0	70.5	78.1				38.1	51.4								11.4									
**Paper 93**	Hyaluronic Acid-Methotrexate Conjugates Coated Magnetic Polydopamine Nanoparticles for Multimodal Imaging-Guided Multistage Targeted Chemo-Photothermal Therapy	DOX	MTX	PDA	inorganic	nonPEG	co-delivery	no	allograft	breast	4T1	sensitive	intravenous	8	21	14	14	0	100.0	60.0											0.0				0.0			32.5					
**Paper 94**	NIR-controlled morphology transformation and pulsatile drug delivery based on multifunctional phototheranostic nanoparticles for photoacoustic imaging-guided photothermal-chemotherapy	DOX	CPT	PPy	polymer	nonPEG	co-delivery	no	allograft	breast	4T1	sensitive	intravenous	1	x	21	0	21	100.0	29.1	38.8						16.5				0.0							87.4					
**Paper 95**	Dual pH/reduction-responsive hybrid polymeric micelles for targeted chemo-photothermal combination therapy	DOX	ICG	x	lipid-polymer	PEG	co-delivery	no	xenograft	liver	BEL-7404	sensitive	intravenous	6	x	20	18	2	100.0	62.4			64.0		32.0			23.2			6.4				0.0								
**Paper 96**	A nanoparticle-based combination chemotherapy delivery system for enhanced tumor killing by dynamic rewiring of signaling pathways	DOX	erlotinib	x	lipid	PEG	co-delivery	yes	xenograft	lung	A549	sensitive	intravenous	1	x	32	0	31	100.0									27.7							1.1								
**Paper 96**	A nanoparticle-based combination chemotherapy delivery system for enhanced tumor killing by dynamic rewiring of signaling pathways	DOX	erlotinib	x	lipid	PEG	co-delivery	yes	xenograft	breast	BT-20	sensitive	intravenous	1	x	32	0	31	100.0									52.1							3.8								
**Paper 97**	A novel GSH responsive poly(alpha-lipoic acid) nanocarrier bonding with the honokiol-DMXAA conjugate for combination therapy [一种新型GSH响应性聚硫辛酸纳米载体键合和厚朴酚-DMXAA缀合物用于联合治疗]	honokiol	DMXAA	x	polymer	PEG	co-delivery	no	allograft	breast	4T1	sensitive	intravenous	5	x	18	16	2	100.0	85.7	22.9		11.4								7.1												
**Paper 98**	Nanoscale covalent organic polymers as a biodegradable nanomedicine for chemotherapy-enhanced photodynamic therapy of cancer	cisplatin	porphyrin	x	other	x	co-delivery	no	allograft	breast	4T1	sensitive	intravenous	1	x	14	1	13	100.0	85.7					71.4	71.4					17.9												
**Paper 99**	Combination drug delivery via multilamellar vesicles enables targeting of tumor cells and tumor vasculature	DOX	CA-4	x	lipid	PEG	co-delivery	no	allograft	Skin	B16F10	sensitive	intravenous	5	21	10	8	2	100.0	68.4	52.6										3.9				1.3								
**Paper 100**	In Situ shRNA Synthesis on DNA–Polylactide Nanoparticles to Treat Multidrug Resistant Breast Cancer	DOX	MDR1 targeting shRNA	x	other	PEG	co-delivery	no	xenograft	breast	MCF-7/MDR	resistant	intravenous	4	49	30	9	21	100.0	87.6					58.2						14.7												
**Paper 101**	Nanocomposite plasters for the treatment of superficial tumors by chemo-photothermal combination therapy	DOX	Au	GO	inorganic	x	co-delivery	no	xenograft	skin	A431	sensitive	on the skin	1	x	16	0	16	100.0		41.7	75.0									0.0								29.2	8.3			
**Paper 102**	Thermosensitive micellar hydrogel for enhanced anticancer therapy through redox modulation mediated combinational effects	PTX	porphyrin	x	polymer	nonPEG	separate	yes	xenograft	liver	HepG2	sensitive	peritumoral	x	x	20	x	x	100.0						30.0	56.7								15.0									

**Paper 103**	Development of redox-responsive theranostic nanoparticles for near-infrared fluorescence imaging-guided photodynamic/chemotherapy of tumor	PTX	Ce6	x	polymer	nonPEG	co-delivery	no	allograft	breast	4T1	sensitive	intravenous	1	x	14	0	14	100.0	83.3	86.7				46.7						10.7												
**Paper 104**	Self-assembly of porphyrin-grafted lipid into nanoparticles encapsulating doxorubicin for synergistic chemo-photodynamic therapy and fluorescence imaging	DOX	porphyrin	x	lipid	PEG	co-delivery	no	allograft	breast	4T1	sensitive	intravenous	x	x	11	1	10	100.0	61.4						52.3					22.7												
**Paper 105**	Oxygen-generating hybrid polymeric nanoparticles with encapsulated doxorubicin and chlorin e6 for trimodal imaging-guided combined chemo-photodynamic therapy	DOX	Ce6	MnO2	polymer	PEG	co-delivery	no	xenograft	breast	MCF-7	sensitive	intravenous	3	x	18	5	13	100.0	51.3					39.5	71.8					10.3							30.8	38.5	53.8			
**Paper 106**	Traceable bioinspired nanoparticle for the treatment of metastatic breast cancer via NIR-trigged intracellular delivery of methylene blue and cisplatin	Cisplatin	MB	x	other	x	co-delivery / separate	no	allograft	breast	4T1	sensitive	intravenous	4	x	17	6	11	100.0				73.2		61.9						0.0			37.0									
**Paper 107**	Tumor-targeted hybrid protein oxygen carrier to simultaneously enhance hypoxia-dampened chemotherapy and photodynamic therapy at a single dose	DOX	Ce6	x	other	x	co-delivery	no	xenograft	breast	MCF-7	sensitive	intravenous	1	x	15	0	15	100.0	48.6			48.1		9.5						0.0												
**Paper 108**	A Metal–Polyphenol Network Coated Nanotheranostic System for Metastatic Tumor Treatments	DOX	Au	x	inorganic	x	co-delivery	no	allograft	liver	H22	sensitive	subcutaneous	2	x	8	6	2	100.0	54.7						47.4					21.1												
**Paper 109**	Hollow MnO2 as a tumor-microenvironment-responsive biodegradable nano-platform for combination therapy favoring antitumor immune responses	DOX	Ce6	x	inorganic	x	co-delivery	no	allograft	breast	4T1	sensitive	intravenous	4	x	17	6	11	100.0						84.2						40.0												
**Paper 109**	Hollow MnO2 as a tumor-microenvironment-responsive biodegradable nano-platform for combination therapy favoring antitumor immune responses	DOX	Ce6	PD-L1	inorganic	x	separate, sequential	no	allograft	breast	4T1	sensitive	intravenous	x	x	18	8	10	100.0						84.2								75.8										
**Paper 110**	Codelivery of sorafenib and GPC3 siRNA with PEI-modified liposomes for hepatoma therapy	sorafenib	siRNA (siGPC3)	x	lipid	PEG	co-delivery	no	xenograft	liver	HepG2	sensitive	intravenous	10	54	30	30	0	100.0						30.7	67.1					4.7												
**Paper 111**	Targetable Clinical Nanoparticles for Precision Cancer Therapy Based on Disease-Specific Molecular Inflection Points	dactolisib	enzalutamide	x	inorganic	x	co-delivery	no	xenograft	prostate	LNCaP	sensitive	intravenous	4	x	7	6	1	100.0				50.0												0.0								
**Paper 111**	Targetable Clinical Nanoparticles for Precision Cancer Therapy Based on Disease-Specific Molecular Inflection Points	cabazitaxel	riluzole	x	inorganic	x	co-delivery	no	xenograft	prostate	PC3-PSMA MDR	resistant	intravenous	14	x	30	28	2	100.0									97.4	42.1						0.0								
**Paper 112**	MiR-375 delivered by lipid-coated doxorubicin-calcium carbonate nanoparticles overcomes chemoresistance in hepatocellular carcinoma	DOX	miR-375	x	inorganic	x	co-delivery	no	xenograft	liver	HepG2/MDR	resistant	intravenous	4	x	16	6	10	100.0	74.1					43.7	63.8					23.7												
**Paper 113**	Carrier-free, self-assembled pure drug nanorods composed of 10-hydroxycamptothecin and chlorin e6 for combinatorial chemo-photodynamic antitumor therapy: In vivo	HCPT	Ce6	x	other	x	co-delivery	no	allograft	breast	4T1	sensitive	intravenous	3	x	12	6	6	100.0	43.1	58.8				43.1						23.5												
**Paper 114**	Sub-100 nm, long tumor retention SN-38-loaded photonic micelles for tri-modal cancer therapy	SN38	pyropheophorbide-a	x	polymer	PEG	co-delivery	yes	xenograft	colon	HT29	sensitive	intravenous	3	x	24	12	12	100.0	54.8						67.7					16.1												
**Paper 114**	Sub-100 nm, long tumor retention SN-38-loaded photonic micelles for tri-modal cancer therapy	SN38	pyropheophorbide-a	x	polymer	PEG	co-delivery	yes	xenograft	colon	HT29	sensitive	intravenous	3	x	24	12	12	100.0	54.8						32.3					9.7												
**Paper 115**	Chemotherapeutic drug-photothermal agent co-self-assembling nanoparticles for near-infrared fluorescence and photoacoustic dual-modal imaging-guided chemo-photothermal synergistic therapy	epirubicin	ICG	x	lipid-polymer	x	co-delivery	no	allograft	breast	4T1	sensitive	intravenous	x	x	20	17	3	100.0	80.7	100.0				56.3						0.0												
**Paper 116**	In vivo comparative study of distinct polymeric architectures bearing a combination of paclitaxel and doxorubicin at a synergistic ratio	DOX	PTX	x	polymer	PEG	co-delivery / separate	yes	allograft	breast	4T1	sensitive	intravenous	5	29	13	8	5	100.0	**64.9**	100.0				79.6	79.7					47.3			69.7									
**Paper 117**	Lanthanide-integrated supramolecular polymeric nanoassembly with multiple regulation characteristics for multidrug-resistant cancer therapy	DOX	antiPgp- siRNA	neodymium	other	x	co-delivery	no	xenograft	breast	MCF-7/MDR	resistant	intravenous	6	x	16	16	0	100.0	80.7					83.0						13.6							56.8					
**Paper 118**	Chemo-photodynamic combined gene therapy and dual-modal cancer imaging achieved by pH-responsive alginate/chitosan multilayer-modified magnetic mesoporous silica nanocomposites	DOX	Ce6	P-gp shRNA	inorganic	x	co-delivery	no	allograft	breast	EMT-6	sensitive	intravenous	x	x	18	x	x	100.0						50.0	65.0					11.7							21.7					
**Paper 119**	Redox-Activatable ATP-Depleting Micelles with Dual Modulation Characteristics for Multidrug-Resistant Cancer Therapy	PTX	PLK1 siRNA	x	polymer	nonPEG	co-delivery	no	xenograft	Colon	HCT8/T	resistant	intratumoral	6	x	21	21	0	100.0	60.3					46.9	52.3					34.3												
**Paper 120**	Precise Engineering of Prodrug Cocktails into Single Polymeric Nanoparticles for Combination Cancer Therapy: Extended and Sequentially Controllable Drug Release	SN38	CA-4	x	polymer	PEG	co-delivery	no	xenograft	Colon	HT29	sensitive	intravenous	3	40	26	x	x	100.0						34.0	92.8					22.4												
**Paper 121**	A novel temperature-responsive micelle for enhancing combination therapy	17-AAG	IR-780	x	polymer	PEG	co-delivery	yes	xenograft	colon	HCT-116	sensitive	intravenous	1	x	29	1	28	100.0						83.8	73.3					15.2												
**Paper 122**	Hyaluronic acid-coated, prodrug-based nanostructured lipid carriers for enhanced pancreatic cancer therapy	GEM	Baicalein	x	lipid	nonPEG	co-delivery	yes	xenograft	pancreas	AsPC1	sensitive	intravenous	11	x	21	20	1	100.0	71.8	78.1		47.1		47.1	54.6					35.3				23.5								
**Paper 123**	Cancer nanomedicines stabilized by π-π stacking between heterodimeric prodrugs enable exceptionally high drug loading capacity and safer delivery of drug combinations	SN38	DTX	x	lipid-polymer	PEG	co-delivery	yes	xenograft	colon	HCT116	sensitive	intravenous	3	x	18	6	12	100.0	39.0											17.5												
**Paper 124**	Aggregation Induced Emission Fluorogens Based Nanotheranostics for Targeted and Imaging-Guided Chemo-Photothermal Combination Therapy	PTX	Pyrole	x	other	x	co-delivery	no	xenograft	brain	U87MG	sensitive	intravenous	1	x	14	0	14	100.0						45.5			60.6							6.8								
**Paper 125**	Comprehensively priming the tumor microenvironment by cancer-associated fibroblast-targeted liposomes for combined therapy with cancer cell-targeted chemotherapeutic drug delivery system	DOX	Navitoclax	x	lipid	PEG	separate, sequential	no	xenograft	liver	HepG2	sensitive	intravenous	4	22	10	4	6	100.0						62.2	50.0		40.8						12.2			13.3						
**Paper 126**	Mild photothermal therapy/photodynamic therapy/chemotherapy of breast cancer by Lyp-1 modified Docetaxel/IR820 Co-loaded micelles	DTX	IR820	x	polymer	PEG	co-delivery	yes	allograft	breast	4T1	sensitive	intravenous	3	x	23	2	21	100.0	38.9	67.8		26.7		27.8	18.9		11.1			3.3				0.0								
**Paper 127**	Combinatorial nanocarrier based drug delivery approach for amalgamation of anti-tumor agents in breast cancer cells: An improved nanomedicine strategies	topotecan	quercetin	x	inorganic	x	co-delivery	no	xenograft	breast	MDA-MB-231	sensitive	intravenous	x	32	24	24	0	100.0	58.4	60.0										28.7				14.6								
**Paper 128**	Exploiting in situ antigen generation and immune modulation to enhance chemotherapy response in advanced melanoma: A combination nanomedicine approach	Cisplatin	CpG	x	lipid	PEG	separate, sequential	no	allograft	skin	B16F10	sensitive	intravenous	6	35	9	7	2	100.0						37.5	37.5								10.0									
**Paper 129**	Targeted nanomedicine for prostate cancer therapy: docetaxel and curcumin co-encapsulated lipid–polymer hybrid nanoparticles for the enhanced anti-tumor activity in vitro and in vivo	DTX	curcumin	x	lipid-polymer	x	co-delivery	no	xenograft	prostate	PC-3	sensitive	intravenous	3	x	21	14	7	100.0	80.2	89.5				54.6	67.4					16.3												
**Paper 130**	Multifunctional theranostic nanomedicine for photoacoustic imaging-guided combination tumor treatment	PTX	BCl2 siRNA	x	other	x	co-delivery	no	xenograft	skin	B16F10	sensitive	intravenous	4	x	16	6	10	100.0						83.2						47.1												
**Paper 131**	Self-Monitoring and Self-Delivery of Photosensitizer-Doped Nanoparticles for Highly Effective Combination Cancer Therapy in Vitro and in Vivo	curcumin	porphyrin	x	other	x	co-delivery	no	xenograft	lung	A549	sensitive	intravenous	14	21	14	14	0	100.0	73.2	34.1										12.2												
**Paper 132**	Co-Eradication of Breast Cancer Cells and Cancer Stem Cells by Cross-Linked Multilamellar Liposomes Enhances Tumor Treatment	DOX	salinomycin	x	lipid	PEG	co-delivery / separate	yes	allograft	breast	4T1	sensitive	intravenous	5	18	10	8	2	100.0						52.3	55.4					23.1			52.3									
**Paper 133**	On-demand CO release for amplification of chemotherapy by MOF functionalized magnetic carbon nanoparticles with NIR irradiation	DOX	ManganCO	x	inorganic	x	co-delivery	no	xenograft	colon	HCT-116	sensitive	intratumoral	1	x	14	0	14	100.0						41.1	100.0					9.5												
**Paper 134**	Drug-induced self-assembly of modified albumins as nano-theranostics for tumor-targeted combination therapy	PTX	Ce6	x	other	x	co-delivery	no	xenograft	brain	U87MG	sensitive	intravenous	1	x	14	0	14	100.0									74.5			45.5				7.3								
**Paper 135**	Combination therapy with epigenetic-targeted and chemotherapeutic drugs delivered by nanoparticles to enhance the chemotherapy response and overcome resistance by breast cancer stem cells	DOX	decitabine	x	polymer	PEG	separate, sequential	no	xenograft	breast	MDA-MB-231	sensitive	intravenous	5	26	12	8	4	100.0	90.0	90.0		50.0		52.5	82.5								35.6									
**Paper 136**	Polymeric nanomedicine for tumor-targeted combination therapy to elicit synergistic genotoxicity against prostate cancer	DOX	5-FU	x	polymer	nonPEG	co-delivery	yes	xenograft	prostate	PC-3	sensitive	intravenous	2	x	21	6	15	100.0	60.0	90.0				47.0	68.0					30.0				18.0								
**Paper 137**	Self-assembled core-shell nanoparticles for combined chemotherapy and photodynamic therapy of resistant head and neck cancers	DOX	pyropheophorbide-a	x	lipid	PEG	co-delivery	yes	xenograft	larynx	SQ20B	resistant	intravenous	2	x	12	7	5	100.0						90.3	100.0					0.0												
**Paper 138**	Anticancer polymeric nanomedicine bearing synergistic drug combination is superior to a mixture of individually-conjugated drugs	DOX	PTX	x	polymer	nonPEG	co-delivery / separate	yes	xenograft	breast	MDA-MB-231	sensitive	intravenous	5	37	26	x	x	100.0	19.0	30.0		19.0		105.0	55.0					8.0			38.0									
**Paper 139**	Combinational delivery of hydrophobic and hydrophilic anticancer drugs in single nanoemulsions to treat MDR in cancer	PTX	5-FU	x	other	x	co-delivery	yes	xenograft	cervix	KB-3-1	sensitive	intravenous	5	x	15	12	3	100.0	18.2					74.3	62.1					40.9												

**Paper 139**	Combinational delivery of hydrophobic and hydrophilic anticancer drugs in single nanoemulsions to treat MDR in cancer	PTX	5-FU	x	other	x	co-delivery	yes	xenograft	cervix	KB-8-5/MDR	resistant	intravenous	5	x	15	12	3	100.0	93.2					69.5	93.2					40.6												
**Paper 140**	Sequential application of a cytotoxic nanoparticle and a PI3K inhibitor enhances antitumor efficacy	Cisplatin	PI818	x	lipid	PEG	separate, sequential	no	allograft	Breast	4T1	sensitive	intravenous + intraperitoneal	6	x	11	10	1	100.0		102.1				81.2								52.1										
**Paper 141**	Antitumor effects of MsurvivinT34A–CaPi complex-embedded PLGA nanoparticles in combination with Doxil in mice	DOX	MsurvivinT34A plasmid	x	other	x	separate, sequential	no	allograft	Breast	4T1	sensitive	intravenous + intratumoral	12	26	22	22	0	100.0						25.1	59.7								9.7									
**Paper 142**	Folding graft copolymer with pendant drug segments for co-delivery of anticancer drugs	DOX	CPT	x	polymer	PEG	co-delivery	no	xenograft	lung	A549	sensitive	intravenous	6	x	22	20	2	100.0	81.7	68.6					40.8					23.7												
**Paper 143**	Engineering the assemblies of biomaterial nanocarriers for delivery of multiple theranostic agents with enhanced antitumor efficacy	DOX	PTX	survivin siRNA	polymer	PEG	co-delivery	no	allograft	skin	B16F10	sensitive	intravenous	3	13	9	6	3	100.0	74.5			62.6	50.0	81.9		100.0				11.7							54.2					
**Paper 144**	Macromolecular nanotheranostics for multimodal anticancer therapy	DOX	GEM	x	polymer	nonPEG	co-delivery	no	allograft	lung	AT1	sensitive	intravenous	x	x	25	x	x	100.0				76.1								54.3												
**Paper 145**	A pH-responsive dissociable mesoporous silica-based nanoplatform enabling efficient dual-drug co-delivery and rapid clearance for cancer therapy	DOX	curcumin	x	inorganic	x	co-delivery	no	xenograft	liver	HepG2	sensitive	intravenous	5	x	14	8	6	100.0						50.0						44.4												
**Paper 146**	Enhancing Triple Negative Breast Cancer Immunotherapy by ICG-Templated Self-Assembly of Paclitaxel Nanoparticles	PTX	ICG	x	other	x	co-delivery	no	allograft	breast	4T1	sensitive	intravenous	1	x	20	1	19	100.0	60.1	38.7										18.3												
**Paper 146**	Enhancing Triple Negative Breast Cancer Immunotherapy by ICG-Templated Self-Assembly of Paclitaxel Nanoparticles	PTX	ICG	PD-L1	other	x	separate, sequential	no	allograft	breast	4T1	sensitive	intravenous	1	x	20	1	19	100.0	60.1	38.7	74.3											0.0										
**Paper 147**	Co-delivery of Poria cocos extract and doxorubicin as an 'all-in-one' nanocarrier to combat breast cancer multidrug resistance during chemotherapy	DOX	pachymicacid	dehydrotumulosic acid	lipid	nonPEG	co-delivery	no	xenograft	breast	MCF-7/MDR	resistant	intravenous	6	x	17	15	2	100.0	54.7					70.6	91.2					35.3												
**Paper 148**	High loading of hydrophobic and hydrophilic agents via small immunostimulatory carrier for enhanced tumor penetration and combinational therapy	PTX	GEM	x	polymer	PEG	co-delivery	yes	allograft	pancreas	PANC02	sensitive	intravenous	5	37	24	12	12	100.0				51.2			46.5					24.2												
**Paper 148**	High loading of hydrophobic and hydrophilic agents via small immunostimulatory carrier for enhanced tumor penetration and combinational therapy	PTX	GEM	x	polymer	PEG	co-delivery	yes	allograft	colon	CT26	sensitive	intravenous	5	x	15	12	3	100.0				62.9			29.7					20.0												
**Paper 149**	Polymeric Hybrid Nanomicelles for Cancer Theranostics: An Efficient and Precise Anticancer Strategy for the Codelivery of Doxorubicin/miR-34a and Magnetic Resonance Imaging	DOX	miR-34a	x	polymer	PEG	co-delivery	no	xenograft	breast	MDA-MB-231	sensitive	intravenous	4	x	18	4	14	100.0	76.3					52.6	76.3					9.2												
**Paper 150**	Zwitterionic polypeptide nanomedicine with dual NIR/reduction-responsivity for synergistic cancer photothermal-chemotherapy	DOX	ICG	x	polymer	PEG	co-delivery	yes	xenograft	cervix	HeLa	sensitive	intravenous	1	x	30	0	30	100.0	95.0			66.0		82.0	45.0					0.0												
**Paper 151**	A novel chitosan-based nanomedicine for multi-drug resistant breast cancer therapy	DOX	oleanolic acid	x	polymer	nonPEG	co-delivery	no	xenograft	breast	MDA-MB-231	sensitive	intravenous	12	x	25	24	1	100.0	34.2	60.3								42.5						0.0								
**Paper 152**	Light-activatable dual prodrug polymer nanoparticle for precise synergistic chemotherapy guided by drug-mediated computed tomography imaging	Cisplatin	cantharidin	x	other	x	co-delivery	yes	xenograft	cervix	U14	sensitive	intravenous	3	x	21	6	15	100.0	100.0	33.3				13.9	27.8					0.0												
**Paper 153**	Enhancing solid tumor therapy with sequential delivery of dexamethasone and docetaxel engineered in a single carrier to overcome stromal resistance to drug delivery	DTX	dexamethasone	x	lipid	PEG	co-delivery	no	xenograft	cervix	KBv	sensitive	intravenous	3	x	9	4	5	100.0	9.6	53.1				6.4	32.2					4.8												
**Paper 153**	Enhancing solid tumor therapy with sequential delivery of dexamethasone and docetaxel engineered in a single carrier to overcome stromal resistance to drug delivery	DTX	dexamethasone	x	lipid	PEG	co-delivery	no	xenograft	cervix	KBv/MDR(DTX)	resistant	intravenous	3	x	11	6	5	100.0	40.0	38.2				32.0	35.2					13.7												
**Paper 153**	Enhancing solid tumor therapy with sequential delivery of dexamethasone and docetaxel engineered in a single carrier to overcome stromal resistance to drug delivery	DTX	dexamethasone	x	lipid	PEG	co-delivery	no	allograft	breast	4T1	sensitive	intravenous	4	x	14	9	5	100.0						33.8	36.2					17.5												
**Paper 154**	Co-delivery of chlorin e6 and doxorubicin using PEGylated hollow nanocapsules for â€ all-in-one' tumor theranostics	DOX	Ce6	CuS	inorganic	x	co-delivery	yes	allograft	breast	4T1	sensitive	intravenous	1	x	25	1	24	100.0	85.0	64.0										4.0								36.0				
**Paper 155**	Hypoxia- and singlet oxygen-responsive chemo-photodynamic Micelles featured with glutathione depletion and aldehyde production	DOX	Ce6	x	polymer	PEG	co-delivery	no	allograft	breast	4T1	sensitive	intravenous	1	x	16	1	15	100.0	65.9			46.0		33.7						15.3												
**Paper 156**	Co-delivery of paclitaxel and PLK1-targeted siRNA using aptamer-functionalized cationic liposome for synergistic anti-breast cancer effects in vivo	PTX	PLK1 siRNA	x	lipid	PEG	co-delivery/separate	no	xenograft	breast	MCF-7	sensitive	intravenous	4	x	21	6	15	100.0	62.5								87.5	84.4		75.0				32.5		62.5						
**Paper 157**	Targeting lipid metabolism to overcome EMT-associated drug resistance via integrin β3/FAK pathway and tumor-associated macrophage repolarization using legumain-activatable delivery	PTX	simvastatin	x	lipid	PEG	co-delivery	no	xenograft	lung	A549T	resistant	intravenous	x	x	27	x	x	100.0	67.1			47.4								13.2				6.6								
**Paper 158**	A nanomedicine approach enables co-delivery of cyclosporin A and gefitinib to potentiate the therapeutic efficacy in drug-resistant lung cancer	gefitinib	Cyclosporin	x	polymer	PEG	co-delivery	no	xenograft	lung	PC-9-GR/MDR EGFR-TKI	resistant	oral + intravenous	3 - 6	x	17	6	11	100.0	100.0			75.8		98.5	121.2					45.5												
**Paper 159**	Remodeling Tumor-Associated Macrophages and Neovascularization Overcomes EGFRT790M-Associated Drug Resistance by PD-L1 Nanobody-Mediated Codelivery	gefitinib	simvastatin	x	lipid	PEG	co-delivery	no	xenograft	lung	H1975/MDR Gef	resistant	intravenous	9	x	18	16	2	100.0	93.3			44.0								23.8				13.1								
**Paper 160**	Evaluation of a novel biocompatible magnetic nanomedicine based on beta-cyclodextrin, loaded doxorubicin-curcumin for overcoming chemoresistance in breast cancer	DOX	Curcumin	x	other	x	co-delivery	yes	allograft	breast	4T1/MDR	resistant	intravenous	3	x	21	14	7	100.0	41.9					27.5						29.0				25.8								
**Paper 161**	Dual drug delivery and sequential release by amphiphilic Janus nanoparticles for liver cancer theranostics	DOX	DTX	Au	inorganic	x	co-delivery	yes	allograft	liver	H22	sensitive	intravenous	2	x	10	6	4	100.0	88.6			65.9		51.9	47.0	18.4				0.0							3.8					
**Paper 162**	Cargo-Free Nanomedicine with pH Sensitivity for Codelivery of DOX Conjugated Prodrug with SN38 to Synergistically Eradicate Breast Cancer Stem Cells	DOX	SN38	x	other	x	co-delivery	yes	xenograft	breast	MCF-7	sensitive	intravenous	5	26	22	8	14	100.0	94.3	34.7		29.1								13.2												
**Paper 163**	A DNA-Based Nanocarrier for Efficient Gene Delivery and Combined Cancer Therapy	DOX	p53 DNA	x	other	x	co-delivery	no	xenograft	breast	MCF-7/MDR	resistant	intravenous	3	x	24	12	12	100.0	62.9								38.8	77.6						20.4								
**Paper 164**	Self-Stabilized Hyaluronate Nanogel for Intracellular Codelivery of Doxorubicin and Cisplatin to Osteosarcoma	DOX	cisplatin	x	polymer	nonPEG	co-delivery	no	allograft	osteosarcoma	K7	sensitive	intravenous	5	36	17	16	1	100.0				37.9												10.9								
**Paper 165**	Enzyme Degradable Hyperbranched Polyphosphoester Micellar Nanomedicines for NIR Imaging-Guided Chemo-Photothermal Therapy of Drug-Resistant Cancers	DOX	IR780	x	polymer	PEG	co-delivery	no	xenograft	breast	MCF-7/MDR	resistant	intravenous	4	x	14	7	7	100.0	60.0					28.0	29.0					6.0												
**Paper 166**	Precision design of nanomedicines to restore gemcitabine chemosensitivity for personalized pancreatic ductal adenocarcinoma treatment	GEM	con siRNA	x	lipid	PEG	co-delivery	no	xenograft	pancreas	Panc1	sensitive	intravenous	x	x	20	x	x	100.0						46.7	100.0					41.7												
**Paper 166**	Precision design of nanomedicines to restore gemcitabine chemosensitivity for personalized pancreatic ductal adenocarcinoma treatment	GEM	RRM2 siRNA	x	lipid	PEG	co-delivery	no	xenograft	pancreas	Panc1-RRM2	sensitive	intravenous	x	x	20	x	x	100.0						70.0	100.0					40.0												
**Paper 167**	Doxorubicin@Bcl-2 siRNA Core@Shell nanoparticles for synergistic anticancer chemotherapy	DOX	(Bcl-2) siRNA	x	polymer	PEG	co-delivery / separate	no	allograft	breast	4T1	sensitive	intravenous	2	x	14	7	7	100.0	73.2	65.4		42.8		32.7	67.3					23.4			25.7									
**Paper 168**	Cascade-amplifying synergistic effects of chemo photodynamic therapy using ROS-responsive polymeric nanocarriers	DOX	Ce6	x	polymer	PEG	co-delivery	no	xenograft	breast	MCF-7/MDR	resistant	intravenous	3	x	16	x	x	100.0	85.3					64.8	69.4					21.6												
**Paper 169**	Targeting intracellular MMPs efficiently inhibits tumor metastasis and angiogenesis	PTX	marimastat	x	other	x	co-delivery	no	allograft	breast	4T1	sensitive	intravenous	5	x	15	12	3	100.0		66.5							66.5							31.0								
**Paper 170**	A drug-self-gated and tumor microenvironment-responsive mesoporous silica vehicle: "four-in-one" versatile nanomedicine for targeted multidrug-resistant cancer therapy	DOX	P-gp siRNA	x	inorganic	x	co-delivery	no	xenograft	breast	MCF-7/MDR	resistant	intravenous	6	x	16	16	0	100.0				64.5					48.4			36.6				21.5								
**Paper 171**	Thermosensitive Liposomal Codelivery of HSA-Paclitaxel and HSA-Ellagic Acid Complexes for Enhanced Drug Perfusion and Efficacy Against Pancreatic Cancer	PTX	ellagic acid	x	lipid	PEG	co-delivery	no	xenograft	pancreas	BxPC-3&HPaSteC	sensitive	intravenous	x	x	15	x	x	100.0	85.0											24.8												
**Paper 172**	Co-delivery of microRNA-21 antisense oligonucleotides and gemcitabine using nanomedicine for pancreatic cancer therapy	Gem	microRNA-21 antisense oligonucleotides	x	inorganic	x	co-delivery	no	xenograft	pancreas	PaCa-2	sensitive	intravenous	2	x	21	6	15	100.0	62.0					40.5	57.0					48.0				35.0								
**Paper 173**	Core-shell hierarchical mesostructured silica nanoparticles for gene/chemo-synergetic stepwise therapy of multidrug-resistant cancer	DOX	Pgp siRNA	x	inorganic	x	co-delivery	no	xenograft	breast	MCF-7/MDR	resistant	intravenous	3	x	13	6	7	100.0	49.3					23.2	90.0					13.0												

**Paper 174**	Luminescent/magnetic PLGA-based hybrid nanocomposites: A smart nanocarrier system for targeted codelivery and dual-modality imaging in cancer theranostics	DOX	VEGF-shRNA	x	polymer	PEG	co-delivery	no	allograft	breast	EMT-6	sensitive	intratumoral	4	27	21	18	3	100.0									26.5	58.8						23.5								
**Paper 175**	Chitosan nanoparticle-mediated co-delivery of shAtg-5 and gefitinib synergistically promoted the efficacy of chemotherapeutics through the modulation of autophagy	gefitinib	shAtg-5-shRNA	x	polymer	nonPEG	co-delivery	no	xenograft	liver	PLC	sensitive	intraperitoneal	15	x	19	17	2	100.0	64.3			58.3		22.6						7.1												
**Paper 176**	Construction and comparison of different nanocarriers for co-delivery of cisplatin and curcumin: A synergistic combination nanotherapy for cervical cancer	cisplatin	curcumin	x	lipid-polymer	x	co-delivery	yes	xenograft	cervix	HeLa	sensitive	intravenous	3	x	21	21	0	100.0	70.2	79.8				33.0	47.9					12.7												
**Paper 177**	Engineered fusion protein-loaded gold nanocarriers for targeted co-delivery of doxorubicin and erbB2-siRNA in human epidermal growth factor receptor-2+ ovarian cancer	DOX	HER2-siRNA	x	other	x	co-delivery	no	xenograft	ovarian	SK-OV-3	sensitive	intraperitoneal	5	x	8	8	0	100.0									65.4	39.8						15.2								
**Paper 178**	Dual-acting, function-responsive, and high drug payload nanospheres for combining simplicity and efficacy in both self-targeted multi-drug co-delivery and synergistic anticancer effect	HCPT	MTX	x	lipid-polymer	x	co-delivery	yes	xenograft	cervix	HeLa	sensitive	intravenous	x	x	14	x	x	100.0	62.5			53.8		56.3						26.3												
**Paper 179**	A nanoparticle carrier for co-delivery of gemcitabine and small interfering rna in pancreatic cancer therapy	GEM	si-Bmi-1RNA	x	inorganic	x	co-delivery	no	xenograft	pancreas	PANC-1	sensitive	intravenous	5	39	21	x	x	100.0	76.5					30.8	73.9			43.1		27.8				22.7								
**Paper 180**	Carrier-Free, Chemophotodynamic Dual Nanodrugs via Self-Assembly for Synergistic Antitumor Therapy	DOX	Ce6	x	other	x	co-delivery	yes	xenograft	breast	MCF-7	sensitive	intravenous	3	x	14	8	6	100.0	64.7	45.9				49.4						0.0												
**Paper 181**	Effects of paclitaxel (PTX) prodrug-based self-assembly peptide hydrogels combined with suberoylanilide hydroxamic acid (SAHA) for PTX-resistant cancer and synergistic antitumor therapy	PTX	Vorinostat	x	polymer	x	co-delivery	yes	allograft	liver	H22	sensitive	intravenous	6	26	12	10	2	100.0	76.8	83.1										37.6												
**Paper 181**	Effects of paclitaxel (PTX) prodrug-based self-assembly peptide hydrogels combined with suberoylanilide hydroxamic acid (SAHA) for PTX-resistant cancer and synergistic antitumor therapy	PTX	Vorinostat	x	polymer	x	co-delivery	yes	allograft	liver	H22	sensitive	intravenous	6	26	12	10	2	100.0	67.0	80.0										25.3												
**Paper 182**	Self-Targeted, Shape-Assisted, and Controlled-Release Self-Delivery Nanodrug for Synergistic Targeting/Anticancer Effect of Cytoplasm and Nucleus of Cancer Cells	CPT	MTX	x	lipid-polymer	x	co-delivery	yes	xenograft	cervix	HeLa	sensitive	intravenous	3	x	14	8	6	100.0	60.6			52.1								28.8												
**Paper 183**	Novel CD44 receptor targeting multifunctional "nano-eggs" based on double pH-sensitive nanoparticles for co-delivery of curcumin and paclitaxel to cancer cells and cancer stem cells	PTX	curcumin	x	polymer	nonPEG	co-delivery	no	xenograft	breast	MDA-MB-231	sensitive	intravenous	10	x	20	18	2	100.0	90.9	93.0		86.4		75.9	66.8					16.6				14.4								
**Paper 184**	Gel-liposome-mediated co-delivery of anticancer membrane-associated proteins and small-molecule drugs for enhanced therapeutic efficacy	DOX	TRAIL	x	lipid	nonPEG	co-delivery	no	xenograft	breast	MDA-MB-231	sensitive	intravenous	7	x	14	12	2	100.0	54.4								32.2							22.2								
**Paper 185**	Enhancing therapeutic effects of docetaxel-loaded dendritic copolymer nanoparticles by co-treatment with autophagy inhibitor on breast cancer	DTX	chloroquine	x	polymer	nonPEG	separate	no	xenograft	breast	MCF-7	sensitive	intraperitoneal	7	x	12	12	0	100.0		67.2				47.5								33.6										
**Paper 186**	Spatiotemporally controlled co-Delivery of anti-Vasculature agent and cytotoxic drug by octreotide-Modified stealth liposomes	DOX	CA-4	x	lipid	PEG	co-delivery / separate	no	xenograft	breast	MCF-7	sensitive	intravenous	7	x	13	12	1	100.0												31.9			46.8	18.6								
**Paper 187**	Rational design of a minimalist nanoplatform to maximize immunotherapeutic efficacy: Four birds with one stone	GEM	1-methyl-tryptophan	x	other	x	co-delivery	no	allograft	skin	B16F10	sensitive	intravenous	5	22	15	13	2	100.0	44.7	79.5		19.8								12.2												
**Paper 188**	Efficiency of Different Treatment Regimens Combining Anti-tumor and Anti-inflammatory Liposomes for Metastatic Breast Cancer	DOX	celecoxib	curcumin	lipid	PEG	separate [drug1, (drug2+3)]	no	allograft	breast	4T1	sensitive	intravenous	6	34	24	21	3	100.0						54.3									25.7									
**Paper 189**	Glioblastoma Therapy Using Codelivery of Cisplatin and Glutathione Peroxidase Targeting siRNA from Iron Oxide Nanoparticles	cisplatin	glutathione peroxidase 4 siRNA	x	inorganic	x	co-delivery	no	xenograft	brain	U87MG	sensitive	intracranial	6	19	12	10	2	100.0	77.0					46.7						34.8				13.9								
**Paper 190**	Dual drug-loaded nano-platform for targeted cancer therapy: Toward clinical therapeutic efficacy of multifunctionality	PTX	gambogic acid	x	lipid	PEG	co-delivery	yes	xenograft	breast	MDA-MB-231	sensitive	intravenous	7	x	15	12	3	100.0	67.9	74.0		55.5		60.2	62.7					47.9				24.8								
**Paper 191**	Bone marrow mesenchymal stem cells-derived exosomes for penetrating and targeted chemotherapy of pancreatic cancer	PTX	GEM	x	other	x	co-delivery	no	xenograft	pancreas	MiaPaca-2	sensitive	intravenous	6	38	25	15	10	100.0	54.7			36.5		23.4						4.3												
**Paper 192**	Non-small cell lung cancer combination therapy: Hyaluronic acid modified, epidermal growth factor receptor targeted, pH sensitive lipid-polymer hybrid nanoparticles for the delivery of erlotinib plus bevacizumab	erlotinib	bevacizumab	x	lipid-polymer	x	co-delivery	yes	xenograft	lung	A549	sensitive	intravenous	8	x	21	21	0	100.0	81.7	88.3		61.7					43.5	50.6		38.8				20.3								
**Paper 193**	Fabrication of doxorubicin-gated mesoporous polydopamine nanoplatforms for multimode imaging-guided synergistic chemophotothermal therapy of tumors	DOX	perfluoro pentane	PDA	inorganic	x	co-delivery	no	xenograft	prostate	PC-3	sensitive	intravenous	3	x	14	x	x	100.0	65.7							57.1				7.9								18.6				
**Paper 194**	Spatiotemporally Targeted Nanomedicine Overcomes Hypoxia-Induced Drug Resistance of Tumor Cells after Disrupting Neovasculature	bortezomib	CA-4P	x	polymer	PEG	co-delivery	no	xenograft	lung	A549	sensitive	intravenous	4	x	15	12	3	100.0	53.0	53.0		53.0		38.0	43.3					12.1												
**Paper 195**	Multifunctional theranostic nano systems enabling photothermal-chemo combination therapy of triple-stimuli-responsive drug release with magnetic resonance imaging	DOX	PDA	x	inorganic	x	co-delivery	no	xenograft	cervix	HeLa	sensitive	intratumoral	1	x	14	1	13	100.0	40.8								35.2	26.8						0.0								
**Paper 196**	Nanoscale platform for delivery of active IRINOX to combat pancreatic cancer	platin	SN38	x	polymer	PEG	co-delivery	yes	xenograft	pancreas	HPAF	sensitive	intravenous	4	x	36	12	24	100.0				71.0		55.0						6.0												
**Paper 197**	Novel t7-modified ph-responsive targeted nanosystem for co-delivery of docetaxel and curcumin in the treatment of esophageal cancer	DTX	curcumin	x	other	PEG	co-delivery	yes	xenograft	Esophagus	KYSE150	sensitive	intravenous	7	x	15	12	3	100.0	68.4					47.8			34.8			44.3				15.2								
**Paper 198**	A Multistage Cooperative Nanoplatform Enables Intracellular Co-Delivery of Proteins and Chemotherapeutics for Cancer Therapy	DOX	ribonuclease	x	polymer	PEG	co-delivery	yes	allograft	skin	B16F10	sensitive	intravenous	4	x	14	9	5	100.0	52.3					60.0	69.1					30.0												
**Paper 199**	Targeted rod-shaped mesoporous silica nanoparticles for the co-delivery of camptothecin and survivin shRNA in to colon adenocarcinoma in vitro and in vivo	CPT	iSur-shRNA	x	inorganic	x	co-delivery	no	allograft	colon	CT26	sensitive	intravenous	4	x	24	9	15	100.0	74.5					52.3	39.0					42.5				14.0								
**Paper 200**	Synergistic Antitumor Effects of Doxorubicin-Loaded Carboxymethyl Cellulose Nanoparticle in Combination with Endostar for Effective Treatment of Non-Small-Cell Lung Cancer	DOX	Endostatin	x	polymer	nonPEG	separate	no	xenograft	lung	A549	sensitive	intravenous	4	x	18	12	6	100.0	36.9	75.6		27.5		24.4								13.6										
**Paper 201**	Inhibition of tumor growth by targeted anti-EGFR/IGF-1R nanobullets depends on efficient blocking of cell survival pathways	Anti EGFR Ab	anti-IGFR kinase inhibitor AG538	x	lipid	PEG	co-delivery / separate	no	xenograft	breast	MDA-MB-468	sensitive	intravenous	10	x	52	x	x	100.0						75.0	64.4					47.2			52.8									
**Paper 202**	Co-delivery of Cu(I) chelator and chemotherapeutics as a new strategy for tumor theranostic	DOX	Cu Chelator	x	polymer	PEG	co-delivery	no	xenograft	breast	MDA-MB-231	sensitive	intravenous	3	x	30	14	16	100.0	61.0	78.0							43.9	56.1		36.6				18.3								
**Paper 203**	AS1411 Aptamer/Hyaluronic Acid-Bifunctionalized Microemulsion Co-Loading Shikonin and Docetaxel for Enhanced Antiglioma Therapy	DTX	shikonin	x	lipid-polymer	x	co-delivery	yes	xenograft	brain	U87	sensitive	intravenous	x	x	x	x	x	100.0				84.4								48.4				35.9								
**Paper 204**	A light-facilitated drug delivery system from a pseudo-protein/hyaluronic acid nanocomplex with improved anti-tumor effects	DOX	AlPcS2a	x	polymer	PEG	co-delivery	yes	xenograft	breast	MDA-MB-231	sensitive	intravenous	3	x	27	15	12	100.0	90.9								48.9	71.6						19.3								
**Paper 205**	Synergistically enhanced anticancer effect of codelivered curcumin and siPlk1 by stimuli-responsive α-lactalbumin nanospheres	Curcumin	siPlk1	x	polymer	nonPEG	co-delivery	no	xenograft	cervix	HeLa	sensitive	intravenous	8	x	16	16	0	100.0	53.5	72.5		53.3		45.8	54.3					40.8				33.3								
**Paper 206**	Co-delivery of aurora-A inhibitor XY-4 and Bcl-xl siRNA enhances antitumor efficacy for melanoma therapy	Aurora-A kinase inhibitor XY-4 i	Bcl-x1 siRNA	x	lipid	nonPEG	co-delivery	no	allograft	skin	B16	sensitive	intratumoral	8	20	17	16	1	100.0						59.0	73.8					32.8												
**Paper 207**	Rational Design of Multi-Stimuli-Responsive Nanoparticles for Precise Cancer Therapy	PTX	Cypate	x	polymer	PEG	co-delivery	yes	allograft	breast	4T1	sensitive	intravenous	1	x	16	1	15	100.0	96.4	26.4				85.5						0.0												
**Paper 208**	Simultaneous delivery of cytotoxic and biologic therapeutics using nanophotoactivatable liposomes enhances treatment efficacy in a mouse model of pancreatic cancer	bevacizumab	visudyne	x	lipid	PEG	co-delivery	no	xenograft	pancreas	AsPC-1	sensitive	intravenous	x	x	35	x	x	100.0	52.6	60.5		26.3		51.3	59.2					2.6												
**Paper 209**	Theranostic vitamin E TPGS micelles of transferrin conjugation for targeted co-delivery of docetaxel and ultra bright gold nanoclusters	DTX	Au	x	Polymer	PEG	co-delivery	no	xenograft	breast	MDA-MB-231	sensitive	intravenous	4	24	12	12	0	100.0	76.3	105.3										47.4				23.7								
**Paper 210**	Nanostructured lipid carriers as multifunctional nanomedicine platform for pulmonary co-delivery of anticancer drugs and siRNA	PTX	BCL2 siRNA	x	lipid	PEG	co-delivery	no	xenograft	lung	A549	sensitive	inhalation	8	x	24	24	0	100.0	69.2					17.5										0.0								
**Paper 211**	Co-delivery of cpt-11 and panobinostat with anti-gd2 antibody conjugated immunoliposomes for targeted combination chemotherapy	CPT	panobinostat	x	lipid	PEG	co-delivery	yes	xenograft	brain	U87MDR	resistant	intravenous	4	14	8	6	2	100.0												75.0				51.7								
**Paper 212**	Combination of magnetic targeting with synergistic inhibition of NF-κB and glutathione via micellar drug nanomedicine enhances its anti-tumor efficacy	celastrol	sulfasalazine	x	other	x	co-delivery	yes	allograft	ascite	Erlich ascite	sensitive	intravenous	x	x	21	x	x	100.0	70.0	85.0		55.0								40.5				20.0								

**Paper 213**	Pharmacophore hybridisation and nanoscale assembly to discover self-delivering lysosomotropic new-chemical entities for cancer therapy	napabuscain	Lys05	x	lipid	nonPEG	co-delivery / free drug	yes	xenograft	pancreas	PCSC	sensitive	intravenous + intraperitoneal	9	x	44	24	20	100.0	101.9					53.7						27.8		72.2										
**Paper 214**	Injectable thermo-sensitive chitosan hydrogel containing CPT-11-loaded EGFR-targeted graphene oxide and SLP2 shRNA for localized drug/gene delivery in glioblastoma therapy	CPT	cetuximab	SLP2 shRNA	other	PEG	co-delivery	no	xenograft	brain	U87	sensitive	intratumoral	1	x	11	0	11	100.0								103.8				42.5												
**Paper 215**	Programmed Size-Changeable Nanotheranostic Agents for Enhanced Imaging-Guided Chemo/Photodynamic Combination Therapy and Fast Elimination	CPT	MeOTTMN	x	inorganic	x	co-delivery	no	allograft	colon	CT26	sensitive	intravenous	1	x	21	1	20	100.0	43.5	80.5										12.3												
**Paper 216**	Chemotherapeutic potency stimulated by SNAI1-knockdown based on multifaceted nanomedicine	DOX	SNAI1-silencing shRNA	x	polymer	nonPEG	co-delivery	no	xenograft	lung	A549	resistant	intravenous	4	x	15	6	9	100.0									33.1	34.9						13.4								
**Paper 217**	Multicomponent-assembled nanodiamond hybrids for targeted and imaging guided triple-negative breast cancer therapy: Via a ternary collaborative strategy	Curcumin	IR780	x	inorganic	x	co-delivery	no	xenograft	breast	MDA-MB	sensitive	intravenous	x	x	12	1	11	100.0	109.4	67.6							66.4	31.6						0.0								
**Paper 218**	NO-releasing polypeptide nanocomposites reverse cancer multidrug resistance via triple therapies	DOX	NO	PDA	polymer	PEG	co-delivery	yes	xenograft	breast	MCF-7/MDR	resistant	intravenous	x	x	20	x	x	100.0	97.1					62.8	40.7					0.0												
**Paper 219**	Liposome co-encapsulation of anti-cancer agents for pharmacological optimization of nanomedicine-based combination chemotherapy	DOX	mitomycin-C	alendronate	lipid	PEG	co-delivery / separate	no	allograft	breast	4T1	sensitive	intravenous	2	20	15	12	3	100.0	96.6					51.3	79.0					47.3										49.1		
**Paper 219**	Liposome co-encapsulation of anti-cancer agents for pharmacological optimization of nanomedicine-based combination chemotherapy	DOX	mitomycin-C	alendronate	lipid	PEG	co-delivery / separate	no	allograft	lung	M109	sensitive	intravenous	2	20	15	12	3	100.0						54.6	26.0					54.6										40.4		
**Paper 219**	Liposome co-encapsulation of anti-cancer agents for pharmacological optimization of nanomedicine-based combination chemotherapy	DOX	mitomycin-C	alendronate	lipid	PEG	co-delivery / separate	no	allograft	lung	M109 MDR	sensitive	intravenous	2	25	20	7	13	100.0						59.5	30.0					22.2							39.1			41.8		
**Paper 220**	Anticancer nanocage platforms for combined immunotherapy designed to harness immune checkpoints and deliver anticancer drugs	DOX	PD-L1	x	other	x	co-delivery	no	allograft	colon	CT26	sensitive	intravenous	10	x	18	18	0	100.0	62.3	69.4					83.1					39.3												
**Paper 221**	Self-assembled fluorescent hybrid nanoparticles-mediated collaborative lncRNA CCAT1 silencing and curcumin delivery for synchronous colorectal cancer theranostics	Curcumin	lncRNA CCAT1	x	lipid-polymer	PEG	co-delivery	no	xenograft	colon	HT29	sensitive	intravenous	7	x	13	13	0	100.0	91.3					59.0	53.4					29.5												
**Paper 222**	NIR-laser-triggered gadolinium-doped carbon dots for magnetic resonance imaging, drug delivery and combined photothermal chemotherapy for triple negative breast cancer	DOX	IR825	x	inorganic	x	co-delivery	no	allograft	breast	4T1	sensitive	intravenous	1	x	14	0	14	100.0						58.9	37.9					12.6												
**Paper 223**	Theranostic potential of biodegradable polymeric nanoparticles with paclitaxel and curcumin against breast carcinoma	PTX	curcumin	x	polymer	PEG	co-delivery	yes	xenograft	breast	4T1	sensitive	intravenous	6	25	18	15	3	100.0	71.1	76.8		64.9					49.4	53.6		59.3				39.5								
**Paper 224**	Albumin-stabilized layered double hydroxide nanoparticles synergized combination chemotherapy for colorectal cancer treatment	5-FU	PTX	x	other	x	co-delivery	yes	xenograft	colon	HCT116	sensitive	intravenous	3	x	10	8	2	100.0						62.6	41.7					8.7												
**Paper 225**	Precisely engineering a carrier-free hybrid nanoassembly for multimodal DNA damage-augmented photodynamic therapy	etoposide	pyropheophorbide a	x	lipid-polymer	PEG	co-delivery	yes	allograft	breast	4T1	sensitive	intravenous	1	x	11	1	10	100.0	84.2	76.3		67.9		56.3						24.7												
**Paper 226**	Multifunctional Size-Expandable Nanomedicines Enhance Tumor Accumulation and Penetration for Synergistic Chemo-Photothermal Therapy	celasterol	ICG	x	polymer	PEG	co-delivery	yes	allograft	skin	B16F10	sensitive	intravenous	1	x	8	1	7	100.0	50.9	10.2		0.0		43.7	7.1					0.0												
**Paper 227**	Multi-functionalized dendrimers for targeted co-delivery of sorafenib and paclitaxel in liver cancers	PTX	sorafenib	x	polymer	nonPEG	co-delivery	no	xenograft	liver	HepG2	sensitive	intravenous	6	x	11	10	1	100.0	84.4	75.4		55.7								42.5				26.2								
**Paper 228**	Vitamin E-facilitated carbon monoxide pro-drug nanomedicine for efficient light-responsive combination cancer therapy	tocopherol	CO	x	lipid	PEG	co-delivery	yes	xenograft	colon	HCT116	sensitive	intravenous	4	x	12	9	3	100.0									61.9	43.3						6.0								
**Paper 229**	Construction of all-in-one peptide nanomedicine with photoacoustic imaging guided mild hyperthermia for enhanced cancer chemotherapy	tyroservatide	porphyrin	x	other	x	co-delivery	no	allograft	breast	4T1	sensitive	intravenous	4	x	21	9	12	100.0						57.6						26.1												
**Paper 230**	Reprogramming Cancer Stem-like Cells with Nanoforskolin Enhances the Efficacy of Paclitaxel in Targeting Breast Cancer	PTX	forskolin	x	lipid-polymer	nonPEG	co-delivery	no	xenograft	breast	MDA-MB-231	sensitive	intravenous	1	101	80	0	80	100.0	29.5	53.8		35.9								0.0												
**Paper 231**	Combination prostate cancer therapy: Prostate-specific membranes antigen targeted, pH-sensitive nanoparticles loaded with doxorubicin and tanshinone	DOX	tanshinones	x	lipid	PEG	co-delivery	yes	xenograft	prostate	Pca	sensitive	intravenous	9	x	18	18	0	100.0				69.5		72.3	74.7					46.2				27.7								
**Paper 232**	Photodynamic-Chemodynamic Cascade Reactions for Efficient Drug Delivery and Enhanced Combination Therapy	DOX	pyropheophorbide a	x	lipid-polymer	PEG	co-delivery	no	xenograft	brain	U87MG	sensitive	intravenous	2	x	14	3	11	100.0	58.1					49.6	57.3					16.0												
**Paper 233**	Synergistic Therapy for Cervical Cancer by Codelivery of Cisplatin and JQ1 Inhibiting Plk1-Mutant Trp53 Axis	cisplatin	Bromodomain Inhibitor	x	polymer	PEG	co-delivery / separate	yes	allograft	cervix	U14	sensitive	intravenous	4	20	16	10	6	100.0		87.3				60.1	60.7					15.3			37.8									
**Paper 234**	A Dual-Responsive Platform Based on Antifouling Dendrimer–CuS Nanohybrids for Enhanced Tumor Delivery and Combination Therapy	DOX	CuS	x	polymer	PEG	co-delivery	no	xenograft	breast	4T1	sensitive	intravenous	1	x	12	0	12	100.0						68.3			58.4			36.9				6.8								
**Paper 235**	A DM1-doped porous gold nanoshell system for NIR accelerated redox-responsive release and triple modal imaging guided photothermal synergistic chemotherapy	maytansine	Herceptin	Au	inorganic	PEG	co-delivery	yes	xenograft	breast	SK-BR-3	sensitive	intravenous	7	x	20	20	0	100.0	41.9					36.8						16.1							30.0					
**Paper 236**	Hyaluronic acid coated liposomes Co-delivery of natural cyclic peptide RA-XII and mitochondrial targeted photosensitizer for highly selective precise combined treatment of colon cancer	RA-XII	porphyrin	x	lipid	PEG	co-delivery	no	allograft	liver	HCT116	sensitive	intravenous	5	x	12	12	0	100.0						64.6	55.8					30.6												
**Paper 237**	Singlet Oxygen-Responsive Polymeric Nanomedicine for Light-Controlled Drug Release and Image-Guided Photodynamic-Chemo Combination Therapy	PTX	boron dipyrromethene	x	polymer	PEG	co-delivery	no	allograft	liver	H22	sensitive	intravenous	3	x	14	4	10	100.0	59.2	85.9				70.6						5.3												
**Paper 238**	Coupling metal organic frameworks with molybdenum disulfide nanoflakes for targeted cancer theranostics	DOX	Dopamine	x	inorganic	x	co-delivery	no	allograft	breast	4T1	sensitive	intravenous	x	x	14	x	x	100.0						37.1	13.3					9.8												
**Paper 239**	Lipid/PAA-coated mesoporous silica nanoparticles for dual-pH-responsive codelivery of arsenic trioxide/paclitaxel against breast cancer cells	PTX	ATO	x	inorganic	x	co-delivery	yes	xenograft	breast	MCF-7	sensitive	intravenous	12	x	21	21	0	100.0				39.3			74.4					26.5				15.4								
**Paper 240**	Co-delivery of nanoparticle and molecular drug by hollow mesoporous organosilica for tumor-activated and photothermal-augmented chemotherapy of breast cancer	disulfiram	CuS	x	inorganic	PEG	co-delivery	no	allograft	breast	4T1	sensitive	intravenous + intragastric	x	x	14	x	x	100.0				66.3		34.8	22.0					12.2												
**Paper 241**	Co-delivery of etoposide and cisplatin in dual-drug loaded nanoparticles synergistically improves chemoradiotherapy in non-small cell lung cancer models	cisplatin	etoposide	x	polymer	PEG	co-delivery / separate	yes	allograft	lung	344SQ	sensitive	intravenous	1	x	9	1	8	100.0				68.7								33.0			56.8									
**Paper 241**	Co-delivery of etoposide and cisplatin in dual-drug loaded nanoparticles synergistically improves chemoradiotherapy in non-small cell lung cancer models	cisplatin	etoposide	x	polymer	PEG	co-delivery / separate	yes	xenograft	lung	H460	sensitive	intravenous	1	x	13	1	12	100.0				65.2								51.8			60.5									
**Paper 242**	Versatile metal-phenolic network nanoparticles for multitargeted combination therapy and magnetic resonance tracing in glioblastoma	cisplatin	DOX	GA/Fe2+ nanoparticles	lipid-polymer	PEG	co-delivery	no	xenograft	brain	U87MG	sensitive	intravenous	5	22	15	12	3	100.0	60.2		50.4									35.2				8.3				75.4				
**Paper 243**	Metal (Au)-Decorated Chitosan-l-Arginine Polymeric Vector for Codelivery of Gefitinib and miR125b for Lung Cancer Therapy	gefitinib	mi125bRNA	x	polymer	nonPEG	co-delivery	no	allograft	lung	LLC	sensitive	intravenous	11	x	20	20	0	100.0	88.6					44.5	72.7					24.3												
**Paper 244**	Stimuli-Sensitive Linear–Dendritic Block Copolymer–Drug Prodrug as a Nanoplatform for Tumor Combination Therapy	DOX	Ce6	x	polymer	PEG	co-delivery	yes	allograft	breast	4T1	sensitive	intravenous	4	27	20	12	8	100.0	59.5	68.2				45.7						21.3												
**Paper 244**	Stimuli-Sensitive Linear–Dendritic Block Copolymer–Drug Prodrug as a Nanoplatform for Tumor Combination Therapy	DOX	Ce6	x	polymer	PEG	co-delivery	yes	allograft	breast	4T1	sensitive	intravenous	4	27	20	12	8	100.0	59.5	68.2				51.0						31.9												
**Paper 244**	Stimuli-Sensitive Linear–Dendritic Block Copolymer–Drug Prodrug as a Nanoplatform for Tumor Combination Therapy	DOX	Ce6	x	polymer	PEG	co-delivery	yes	allograft	breast	4T1	sensitive	intravenous	4	27	20	12	8	100.0	25.8	74.5				48.6						22.0												
**Paper 245**	iRGD Tumor-Penetrating Peptide-Modified Nano-Delivery System Based on a Marine Sulfated Polysaccharide for Enhanced Anti-Tumor Efficiency Against Breast Cancer	oridonin	Bromodomain Inhibitor	x	polymer	nonPEG	co-delivery	yes	allograft	breast	4T1	sensitive	intravenous	5	x	20	8	12	100.0	85.5	73.7		61.2								42.8				17.1								
**Paper 246**	Enhanced tumor homing of pathogen-mimicking liposomes driven by R848 stimulation: A new platform for synergistic oncology therapy	podophyllotoxin	resiquimod	x	other	nonPEG	separate	no	allograft	breast	4T1	sensitive	intravenous	5	18	12	8	4	100.0	83.1					63.5	52.1								25.4									
**Paper 247**	Ce6- and Bez235-Based Nanomedicine for Chemo-/Photodynamic Combination Therapy of Tumors	dactolisib	Ce6	x	other	x	co-delivery	no	xenograft	breast	MCF-7	sensitive	intravenous	1	x	17	0	17	100.0	80.8	79.9				77.9						9.6												

**Paper 248**	Multifunctional FeS2@SRF@BSA nanoplatform for chemo-combined photothermal enhanced photodynamic/chemodynamic combination therapy	sorafenib	FeS2	x	other	x	co-delivery	no	allograft	breast	4T1	sensitive	intravenous	1	x	20	0	20	100.0	85.4	29.1				51.3	14.6					4.2												
**Paper 249**	Magnetic lipid nanovehicles synergize the controlled thermal release of chemotherapeutics with magnetic ablation while enabling non-invasive monitoring by MRI for melanoma theranostics	DOX	iron oxide	x	lipid	nonPEG	co-delivery	no	allograft	skin	B16F10	sensitive	intravenous	3	14	6	4	2	100.0	75.0	58.8				76.1						34.4												
**Paper 250**	Synthesis of doxorubicin-loaded peptosomes hybridized with gold nanorod for targeted drug delivery and CT imaging of metastatic breast cancer	DOX	gold	x	polymer	PEG	co-delivery	no	allograft	breast	4T1	sensitive	intravenous	1	x	18	0	18	100.0	39.2					51.0						51.0				8.8								
**Paper 251**	In Situ Nanoparticle Self-Assembly for Combination Delivery of Therapeutics to Non-Small Cell Lung Cancer	GEM	gambogicacid	x	other	x	co-delivery	yes	xenograft	lung	A549	sensitive	intraperitoneal	8	x	28	28	0	100.0	65.1	88.4		34.9								23.1												
**Paper 252**	Brain-Targeted Codelivery of Bcl-2/Bcl-xl and Mcl-1 Inhibitors by Biomimetic Nanoparticles for Orthotopic Glioblastoma Therapy	Navitoclax	Mcl-1 inhibitor A12	x	other	x	co-delivery	yes	xenograft	brain	U87 MG MDR	resistant	intravenous	5	20	10	8	2	100.0				40.5					33.7	33.7						7.4								
**Paper 253**	Combinational Chemoimmunotherapy for Breast Cancer by Codelivery of Doxorubicin and PD-L1 siRNA Using a PAMAM-Incorporated Liposomal Nanoplatform	DOX	siRNA PD-L1	x	lipid-polymer	PEG	co-delivery	no	xenograft	breast	MCF-7	sensitive	intravenous	5	x	12	8	4	100.0	46.0						76.0					26.0												
**Paper 254**	Targeted Drug/Gene/Photodynamic Therapy via a Stimuli-Responsive Dendritic-Polymer-Based Nanococktail for Treatment of EGFR-TKI-Resistant Non-Small-Cell Lung Cancer	gefitinib	YAP siRNA	Ppa	polymer	PEG	co-delivery	no	xenograft	lung	PC-9/BB4	sensitive	intravenous	5	x	15	13	2	100.0	76.5					59.6	61.3	72.3				5.9							38.6	27.7	27.7			
**Paper 254**	Targeted Drug/Gene/Photodynamic Therapy via a Stimuli-Responsive Dendritic-Polymer-Based Nanococktail for Treatment of EGFR-TKI-Resistant Non-Small-Cell Lung Cancer	gefitinib	YAP siRNA	Ppa	polymer	PEG	co-delivery	no	xenograft	lung	PDX MDR	resistant	intravenous	6	x	17	15	2	100.0	72.5											15.0							56.2					
**Paper 255**	Tumor-Activatable Nanoparticles Target Low-Density Lipoprotein Receptor to Enhance Drug Delivery and Antitumor Efficacy	oxaliplatin	SN38	x	lipid	PEG	co-delivery	no	allograft	colon	CT26	sensitive	intravenous	8	36	24	21	3	100.0				73.9		51.3	78.6					9.6												
**Paper 255**	Tumor-Activatable Nanoparticles Target Low-Density Lipoprotein Receptor to Enhance Drug Delivery and Antitumor Efficacy	oxaliplatin	SN38	x	lipid	PEG	co-delivery	no	allograft	colon	MC38	sensitive	intravenous	6	25	18	15	3	100.0				65.6		48.8	76.8					9.4												
**Paper 255**	Tumor-Activatable Nanoparticles Target Low-Density Lipoprotein Receptor to Enhance Drug Delivery and Antitumor Efficacy	oxaliplatin	SN38	x	lipid	PEG	co-delivery	no	xenograft	colon	HT29	sensitive	intravenous	11	40	35	33	2	100.0				86.1								3.5												
**Paper 255**	Tumor-Activatable Nanoparticles Target Low-Density Lipoprotein Receptor to Enhance Drug Delivery and Antitumor Efficacy	oxaliplatin	SN38	x	lipid	PEG	co-delivery	no	xenograft	colon	HCT116	sensitive	intravenous	7	27	20	18	2	100.0				29.7								1.4												
**Paper 255**	Tumor-Activatable Nanoparticles Target Low-Density Lipoprotein Receptor to Enhance Drug Delivery and Antitumor Efficacy	oxaliplatin	SN38	x	lipid	PEG	co-delivery	no	xenograft	colon	SW480	sensitive	intravenous	7	27	20	18	2	100.0				44.4								0.0												
**Paper 256**	Engineering naphthalimide-cyanine integrated near-infrared dye into ROS-responsive nanohybrids for tumor PDT/PTT/chemotherapy	NBDHEX	ML880	x	inorganic	x	co-delivery	no	xenograft	liver	HepG2	sensitive	intravenous	5	21	15	15	0	100.0						59.1	24.3					7.8												
**Paper 257**	Concurrent silencing of TBCE and drug delivery to overcome platinum-based resistance in liver cancer	cisplatin	TBCE siRNA	x	lipid-polymer	PEG	co-delivery	no	xenograft	liver	BEL-7402R MDR	resistant	intravenous	4	26	12	6	6	100.0	57.3					46.6	84.4					20.4												
**Paper 258**	Co-Delivery of Precisely Prescribed Multi-Prodrug Combination by an Engineered Nanocarrier enables Efficient Individualized Cancer Chemotherapy	PTX	DOX	CPT	polymer	nonPEG	co-delivery	yes	xenograft	breast	MCF-7/MDR	resistant	intravenous	5	x	12	8	4	100.0	80.4	36.8	80.4		50.1							18.4												
**Paper 258**	Co-Delivery of Precisely Prescribed Multi-Prodrug Combination by an Engineered Nanocarrier enables Efficient Individualized Cancer Chemotherapy	PTX	DOX	CPT	polymer	nonPEG	co-delivery	yes	xenograft	breast	MCF-7/MDR	resistant	intravenous	5	x	12	8	4	100.0	69.0	54.6	69.0									19.6												
**Paper 258**	Co-Delivery of Precisely Prescribed Multi-Prodrug Combination by an Engineered Nanocarrier enables Efficient Individualized Cancer Chemotherapy	PTX	DOX	CPT	polymer	nonPEG	co-delivery	yes	allograft	breast	4T1	sensitive	intravenous	5	20	15	12	3	100.0	80.0	43.9	53.3		40.4							35.8				19.5								
**Paper 259**	Bioresponsive immune-booster-based prodrug nanogel for cancer immunotherapy	DOX	Mannose	x	polymer	PEG	co-delivery	no	allograft	breast	4T1	sensitive	intravenous	4	18	11	9	2	100.0	33.7	65.2				33.7	49.4					20.2												
**Paper 260**	Complete ablation of tumors using synchronous chemoradiation with bimetallic theranostic nanoparticles	MTX	Curcumin	Bi2S3	other	x	co-delivery	no	allograft	breast	4T1	sensitive	intravenous	x	x	20	x	x	100.0						22.7		39.1				0.0												
**Paper 261**	Redox-Activatable Theranostic Co-Prodrug for Precise Tumor Diagnosis and Selective Combination Chemotherapy	CDDO-Me	Vorinostat	x	other	x	co-delivery	no	xenograft	lung	A549	sensitive	intravenous	11	x	21	20	1	100.0				49.5												17.4								
**Paper 262**	A self-amplified ROS-responsive chemodrug-inhibitor conjugate for multi-drug resistance tumor therapy	tamoxifen	tocopherol	x	other	x	co-delivery	no	xenograft	breast	MCF-7/MDR	resistant	intravenous	6	x	18	15	3	100.0	70.8			34.9								15.5												
**Paper 263**	Self-assembly nanomicelle-microneedle patches with enhanced tumor penetration for superior chemo-photothermal therapy	PTX	IR780	x	polymer	nonPEG	co-delivery	no	allograft	skin	B16	sensitive	transdermal	1	14	9	0	9	100.0									82.9	25.6						9.8								
**Paper 264**	Membrane-camouflaged supramolecular nanoparticles for co-delivery of chemotherapeutic and molecular-targeted drugs with siRNA against patient-derived pancreatic carcinoma	GEM	erlotinib	siRNA RAK4	other	x	co-delivery	no	xenograft	pancreas	SW199	sensitive	intravenous	6	32	21	7	14	100.0	64.3											23.4							40.5					
**Paper 264**	Membrane-camouflaged supramolecular nanoparticles for co-delivery of chemotherapeutic and molecular-targeted drugs with siRNA against patient-derived pancreatic carcinoma	GEM	erlotinib	siRNA RAK4	other	x	co-delivery	no	xenograft	pancreas	PDX	sensitive	intravenous	6	50	45	28	17	100.0	69.3											21.8							34.6					
**Paper 265**	Artificial tumor microenvironment regulated by first hemorrhage for enhanced tumor targeting and then occlusion for synergistic bioactivation of hypoxia-sensitive platesomes	nitrogen mustard	Ppa	Ca4P	lipid	PEG	co-delivery / separate	yes	allograft	breast	4T1	sensitive	intravenous	4	x	12	9	3	100.0					75.7									49.5			21.3						48.6	
**Paper 266**	Advanced Nanoengineering Approach for Target-Specific, Spatiotemporal, and Ratiometric Delivery of Gemcitabine–Cisplatin Combination for Improved Therapeutic Outcome in Pancreatic Cancer	cisplatin	GEM	x	inorganic	PEG	co-delivery	yes	allograft	pancreas	KCM	sensitive	intravenous	6	34	28	18	10	100.0				60.7								45.0				24.0								
**Paper 267**	Ratiometric co-delivery of doxorubicin and paclitaxel prodrug by remote-loading liposomes for the treatment of triple-negative breast cancer	DOX	PTX	x	lipid	PEG	co-delivery / separate	yes	allograft	breast	4T1	sensitive	intravenous	4	x	11	9	2	100.0	55.2	66.0		48.2		43.1	29.9					12.6			39.1									
**Paper 268**	Intelligent Nanoparticles With pH-Sensitive Co-Delivery of Temozolomide and siEGFR to Ameliorate Glioma Therapy	temozolomide	EGFR-siRNA	x	polymer	PEG	co-delivery	no	xenograft	brain	U87	sensitive	intravenous	3	30	18	4	14	100.0	81.5			53.5								36.2				10.3								
**Paper 269**	A Hybrid Supramolecular Polymeric Nanomedicine for Cascade-Amplified Synergetic Cancer Therapy	CPT	iron oxide	x	other	PEG	co-delivery	no	allograft	breast	4T1	sensitive	intravenous	3	x	17	6	11	100.0	75.0											24.5												
**Paper 269**	A Hybrid Supramolecular Polymeric Nanomedicine for Cascade-Amplified Synergetic Cancer Therapy	CPT	iron oxide	PD-L1	other	PEG	co-delivery / separate	no	allograft	breast	4T1	sensitive	intravenous	x	x	17	6	11	100.0	60.9				49.1									12.1							27.1			
**Paper 270**	Image-guided drug delivery of nanotheranostics for targeted lung cancer therapy	gefitinib	genistein	x	other	x	separate	yes	allograft	lung	LLC	sensitive	intravenous	11	x	13	11	2	100.0	84.5			56.4											28.6									
**Paper 271**	Construction of pH-sensitive targeted micelle system co-delivery with curcumin and dasatinib and evaluation of anti-liver cancer	curcumin	dasatinib	x	other	x	co-delivery	no	xenograft	liver	HepG2	sensitive	intravenous	6	29	18	12	6	100.0	86.2			64.8												52.4								
**Paper 272**	Co-Delivery of Doxorubicin and Anti-PD-L1 Peptide in Lipid/PLGA Nanocomplexes for the Chemo-Immunotherapy of Cancer	DOX	PD-L1 inhibitor	x	lipid-polymer	PEG	co-delivery	no	allograft	colon	CT26	sensitive	intravenous	6	x	12	10	2	100.0	76.8	90.4		64.0					60.0	76.8		51.1				38.4								
**Paper 273**	Self-Delivery Nanomedicine for Glutamine-Starvation Enhanced Photodynamic Tumor Therapy	ASCT2 inhibitor V9302	Ce6	x	lipid-polymer	PEG	co-delivery	no	allograft	colon	CT26	sensitive	intravenous	2	x	13	11	x	100.0	89.8	51.0		34.7		77.5						12.2												

Abbreviations

17-AAG	17-(Allylamino)-17-demethoxygeldanamycin
5-FU	5-Fluoruricil
ACST	Alanine, Serine, Cysteine Transporter 2
AlPcS2a	Al(III) phthalocyanine chloride disulfonic acid
ATO	Arsenic trioxide
BCL	B-cell lymphoma
BNN6	N,N'-di-sec-butyl-N,N'-dinitroso-1,4-phenylenediamine
BODIPY	4,4-difluoro-4-bora-3a,4a-diaza-s-indacene
CA4	Combretastatin-A4
CCAT1	Colon cancer associated transcript 1
CDDO-Me	Methyl-2-cyano-3,12-dioxooleana-1,9(11)-dien-28-oate
Ce6	Chlorine e6
CO	Carbon monoxide
CPP	Cell penetrating peptide

CPT	Camptothecin
CTLA-4	Cytotoxic T-lymphocyte-associated Protein 4
Cu2O	Copper oxide
CuS	Copersulfid
DMXAA	d 5,6-dimethylxanthenone-4-acetic acid
DOX	Doxorubicin
EGFR	Epidermal Growth Factor Receptor
FA	Folic acid
GEM	Gemcitabine
GO	Graphene oxide
GPC3	Glypican 3
HA	Hyaluronic acid
HCPT	10-Hydroxycamptothecin
HER2	Human epidermal growth factor receptor 2
ICG	Indocyanine green
IDO	Indolamin-2,3-Dioxygenase
IF7	IFLLWQR
IR	Cyanine dye
IR	Infrared
LHRH	Luteinizing hormone releasing hormone
Lys	Lysine
MB	Methylenblue
Mcl	Mantle cell lymphoma
MDR	Multidrug resistance
miRNA	microRNA
MnO2	Mangan(IV)-oxide
MTX	Methotrexate
MUC1	Mucin 1
NO	Nitrogen monoxide
PD-1	Programmed cell death protein 1
PDA	Polydopamine
PEDF	Pigment epithelium-derived factor
PEG	Polyethylenglycol
PFNS	Perfluorononanesulfonic acid
P-gp	P-glycoprotein
PI103	PI3K inhibitor
Plk1	Polo-like-Kinase 1
Ppa	Pyropheophorbide a
PPy	Polypyrrole
PSMA	Prostate-specific membrane antigen
PTX	Paclitaxel
RRM2	Ribonucleoside-diphosphate reductase subunit M2
scFv	Single chain variable fragment)
SLP2	Stomatin Like 2
SN38	7-Ethyl-10-hydroxycamptothecin
SNAI1	Snail Family Transcriptional Repressor 1
TBCE	Tubulin-specific chaperone E
THPP	Mesotetra(p-hydroxyphenyl) porphine
TRAF	TNF receptor associated factor
TRAIL	TNF-related apoptosis-inducing ligand
VDA	Vascular disrupting agent
VEGF	Vascular Endothelial Growth Factor
YAP	Yes-associated protein 1

Supplementary table 2 | Meta-analytic one-to-one comparison of combination therapy versus combination nanotherapy.

Study or Subgroup	Combination therapy Mean	SD	Total	Combination nanotherapy Mean	SD	Total	Weight	Std. Mean Difference IV, Random, 95% CI
Markovsky 2014 b	161	2	4	302	54	4	1.1%	-3.21 [-5.81, -0.60]
Chen 2017	800	0	5	252	0	5		Not estimatable
Chen 2019	0.5	0	3	0.1	0	3		Not estimatable
Chen 2020	625	46	6	143	0	6		Not estimatable
Ding 2019	993	92	4	0	0	4		Not estimatable
He 2022	28.6	10.7	6	5.24	0	6		Not estimatable
Jiang 2022 d	0.62	0.05	6	0.03	0	6		Not estimatable
Jiang 2022 e	0.79	0.23	6	0	0	6		Not estimatable
Li 2016 b	240	61	5	0	0	5		Not estimatable
Li 2022 b	4.25	0.6	5	3.758	0	5		Not estimatable
Li 2022 c	4.25	0.6	5	2.045	0	5		Not estimatable
Lil, Yang 2021	0	0	5	0	0	5		Not estimatable
Liu 2019 a	518	45	6	518	30	6		Not estimatable
Luo 2018	505	483	7	0	0	7		Not estimatable
Prasad 2019	6,154	903	6	0	0	6		Not estimatable
Singh 2021	524	90	4	0	0	4		Not estimatable
Sun 2020 a	1,151	348	5	393	0	5		Not estimatable
Sun 2020 b	1,156	66	5	172	0	5		Not estimatable
Tsai 2017 b	1,910	40	5	480	0	5		Not estimatable
Zhai 2018 b	1.74	0.18	5	0	0	5		Not estimatable
Zhang, Wang 208	1,639	488	7	468	0	0		Not estimatable
Zhang 201	12.2	0.6	5	0	0	5		Not estimatable
Zhou 2019	50	0	8	5.9	0	8		Not estimatable
Wang 2016 a	1,357	500	6	1,214	200	6	1.4%	0.35 [-0.80, 1.49]
Liu 2014 c	520	14	6	500	51	6	1.4%	0.49 [-0.66, 1.65]
Mei 2019 b	6.7	2.23	6	5	3.21	6	1.4%	0.57 [-0.60, 1.73]
Zhou 2020	315	244	7	193	120	7	1.5%	0.59 [-0.49, 1.67]
Zhang, Qin 2018	200	130	5	125	40	5	1.4%	0.70 [-0.60, 2.01]
Li 2019 a	640	173	6	490	157	6	1.4%	0.84 [-0.37, 2.04]
Kim 2021 a	732	32	3	695	26	3	1.3%	1.02 [-0.86, 2.89]
Ramesh 2019 a	1,000	245	5	709	245	5	1.4%	1.07 [-0.31, 2.45]
Zeng 2021	819	96	3	662	106	3	1.3%	1.24 [-0.76, 3.24]
Zhang 2016	2,333	600	6	1,583	400	6	1.4%	1.36 [0.05, 2.67]
Hatami 2022	244	41	4	162	52	4	1.3%	1.52 [-0.21, 3.26]
Lei 2019 a	450	50	5	375	25	5	1.4%	1.71 [0.14, 3.28]
Li 2019 b	640	173	6	400	54	6	1.4%	1.73 [0.32, 3.14]
Li 2014	400	81	6	285	30	6	1.4%	1.74 [0.32, 3.15]
Zhang, Hagan 2021 b	0.73	0.1	6	0.57	0.05	6	1.4%	1.87 [0.41, 3.32]
Zhai 2018 a	1.74	0.18	5	1.16	0.35	5	1.4%	1.88 [0.25, 3.51]
Guo 2012 a	640	156	5	386	66	5	1.4%	1.92 [0.27, 3.56]
Zhang 2022 a	6.23	0.41	5	4.98	0.71	5	1.4%	1.95 [0.29, 3.60]
Zhang 2021 a	460	50	5	310	80	5	1.3%	2.03 [0.34, 3.72]
Mei 2019 a	6.7	2.23	6	2.5	1.48	6	1.4%	2.05 [0.54, 3.56]
Lei 2020	368	203	8	41	65	8	1.4%	2.05 [0.77, 3.33]
Yin 2018 a	685	190	6	370	53	6	1.4%	2.08 [0.56, 3.61]
Kang 2018 a	1,213	235	4	717	124	4	1.3%	2.30 [0.19, 4.40]
Wang 2016 b	1,357	500	6	429	150	6	1.4%	2.32 [0.72, 3.92]
Sun 2018	770	73	5	350	203	5	1.3%	2.49 [0.62, 4.36]
Zhang 2022 b	6.23	0.41	5	3.74	1.04	5	1.3%	2.85 [0.82, 4.87]
Zhao 2022 a	490	160	5	130	10	5	1.3%	2.87 [0.83, 4.90]
Yin 2018 b	685	190	5	204	76	5	1.3%	3.00 [0.91, 5.10]
Cheng 2017 b	615	80	5	357	74	5	1.3%	3.02 [0.92, 5.13]
Lee 2020	189	49.2	5	55	26.8	5	1.3%	3.06 [0.93, 5.18]
Lei 2019 b	450	50	5	290	40	5	1.2%	3.19 [1.01, 5.37]
Wang 2017	574	125	4	202	57	4	1.1%	3.33 [0.65, 6.01]
Li 2016	4.3	0.7	4	2.1	0.4	4	1.1%	3.36 [0.66, 6.05]
Liu 2019	475	90	5	220	31	5	1.2%	3.42 [1.13, 5.71]
Tarannum 2021 a	266	18	5	197	18	5	1.2%	3.46 [1.15, 5.77]
Han 2018 a	1,162	252	4	324	156	4	1.1%	3.48 [0.71, 6.24]
Ramesh 2019 b	940	156	5	70	268	5	1.2%	3.58 [1.22, 5.95]
Ma 2021 b	1,003	71	4	765	40	4	1.1%	3.59 [0.76, 6.42]
Deng 2019	6	1	6	2	1	6	1.3%	3.69 [1.56, 5.82]
Jiang 2022 c	1.66	0.56	6	0.05	0.06	6	1.3%	3.73 [1.59, 5.88]
Li 2022 a	681	86	5	250	114	5	1.2%	3.86 [1.36, 6.35]
Zhang, Hagan 2021 a	0.73	0.1	6	0.35	0.08	6	1.2%	3.87 [1.67, 6.08]
Tangutoori 2016	200	38	8	20	48	8	1.3%	3.93 [2.08, 5.78]
Li 2016 a	240	61	5	30	30	5	1.2%	3.95 [1.40, 6.49]
Zhang 2020	7.9	1.2	5	3	1	5	1.2%	4.01 [1.43, 6.58]
Kang 2018 b	1,213	235	4	358	83	4	1.0%	4.22 [1.00, 7.43]
Qu 2015 a	540	25	6	430	22	6	1.2%	4.31 [1.92, 6.70]
Sun 2022	418	68	6	186	2	6	1.2%	4.45 [2.00, 6.90]
Han 2018 b	1,162	252	4	162	110	4	1.0%	4.47 [1.10, 7.85]
Chen 2021 b	851	37	6	594	64	6	1.2%	4.54 [2.05, 7.03]
Jiang 2022 b	0.98	0.24	6	0.11	0.05	6	1.2%	4.63 [2.10, 7.16]
Wang 2021	700	133	5	182	50	5	1.1%	4.66 [1.76, 7.56]
Sun 2015 a	1,100	112	5	600	70	5	1.1%	4.84 [1.84, 7.83]
Tao 2022 b	917	118	5	443	37	5	1.1%	4.90 [1.87, 7.92]
Li 2015	4.9	0.5	6	2.7	0.3	6	1.1%	4.93 [2.27, 7.58]
Yin 2022	3.39	0.37	4	1.71	0.17	4	0.9%	5.07 [1.32, 8.83]
Elhasany 2020 a	110	7	8	81	3	8	1.2%	5.09 [2.84, 7.35]
Tarannum 2021 b	266	18	5	105	36	5	1.0%	5.11 [1.98, 8.24]
Lu 2016 a	420	17	10	320	20	10	1.3%	5.16 [3.17, 7.15]
Liu 2014 b	600	66	6	297	32	6	1.1%	5.39 [2.53, 8.26]
Zhang 2021 b	460	50	5	180	40	5	1.0%	5.59 [2.21, 8.97]
Sun 2015 b	1,100	112	5	400	111	5	1.0%	5.67 [2.25, 9.09]
Cheng 2017 a	615	80	5	210	43	5	1.0%	5.70 [2.26, 9.13]
Wang 2013	1,679	262	6	393	88	6	1.0%	6.07 [2.90, 9.25]
Wang 2015	875	47	6	475	70	6	1.0%	6.19 [2.97, 9.42]
Liu 2014 a	562	18	6	353	37	6	1.0%	6.63 [3.20, 10.06]
Kim 2021 b	732	32	3	439	38	3	0.5%	6.67 [0.03, 13.31]
Ruttala 2017 a	969	60	7	604	40	7	1.0%	6.70 [3.59, 9.81]
Pang 2020 a	693	42	6	437	25	6	1.0%	6.84 [3.31, 10.36]
Tao 2022 a	917	118	5	258	20	5	0.8%	7.03 [2.88, 11.18]
Liu 2019 b	518	45	6	204	35	6	0.9%	7.19 [3.50, 10.88]
Sun 2021 a	987	52	10	657	33	10	1.1%	7.26 [4.60, 9.92]
Yang 2022	790	100	5	194	30	5	0.8%	7.29 [3.00, 11.58]
Markovsky 2014 a	161	2	4	71	15	4	0.7%	7.31 [2.10, 12.53]
Zhang	337	30	5	124	20	5	0.8%	7.55 [3.12, 11.97]
Gaber 2020	201	13	7	112	8	7	0.9%	7.72 [4.19, 11.25]
Ma 2021 a	1,003	71	4	472	43	4	0.6%	7.87 [2.28, 13.45]
Ruttala 2017 b	969	60	7	374	50	7	0.8%	10.09 [5.56, 14.62]
Zehng 2017	653	65	5	80	20	5	0.5%	10.76 [4.58, 16.95]
Ni 2016 b	506	38	6	159	16	6	0.6%	10.99 [5.51, 16.47]
Chen 2021 a	851	37	6	237	62	6	0.6%	11.10 [5.57, 16.64]
Lu 2016 b	420	17	10	211	14	10	0.8%	12.85 [8.32, 17.38]
Sun 2021 b	987	52	10	394	34	10	0.8%	12.93 [8.37, 17.48]
Elhasany 2020 b	110	7	8	40	1	8	0.6%	13.24 [7.86, 18.61]
Ni 2016 a	619	29	6	248	22	6	0.5%	13.31 [6.71, 19.90]
Qu 2015 b	540	25	6	230	12	6	0.4%	14.59 [7.38, 21.81]
Yang 2018 a	846	30	8	412	25	8	0.5%	14.86 [8.85, 20.87]
Jiang 2022 a	1.13	0.08	6	0.15	0.01	6	0.4%	15.87 [8.04, 23.70]
Li 2021	553	20	6	203	19	6	0.4%	16.56 [8.40, 24.73]
Shan 2019	680	20	5	197	30	5	0.3%	17.11 [7.40, 26.82]
Tsai 2017 a	690	40	5	70	20	5	0.3%	17.71 [7.66, 27.76]
Zhang 2018	406	21	3	45	7	3	0.1%	18.45 [0.56, 36.34]
Park 2014	420	15	4	180	5	4	0.2%	18.67 [5.75, 31.58]
Hong 2019	702	28	6	209	20	6	0.3%	18.70 [9.50, 27.90]
Zhang, Song 2021	413	20	5	74	1	5	0.2%	21.62 [9.39, 33.86]
Yang 2018 b	846	30	8	260	15	8	0.3%	23.36 [13.99, 32.73]
Chen 2015 a	2,376	46	6	456	32	6	0.1%	44.73 [22.86, 66.59]
Chen 2015 b	2,376	46	6	397	5	6	0.0%	55.83 [28.55, 83.11]
**Total (95% CI)**			**554**			**554**	**100.0%**	**4.35 [3.80, 4.91]**

Heterogeneity: Tau² = 5.24; Chi² = 490.07, df = 98 (P < 0.00001); I² = 80%
Test for overall effect: Z = 15.34 (P < 0.00001)

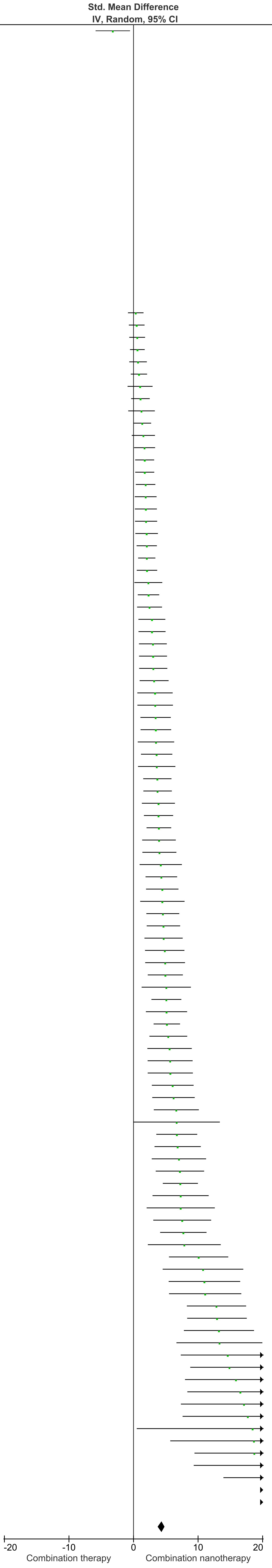

Supplementary Figure 1

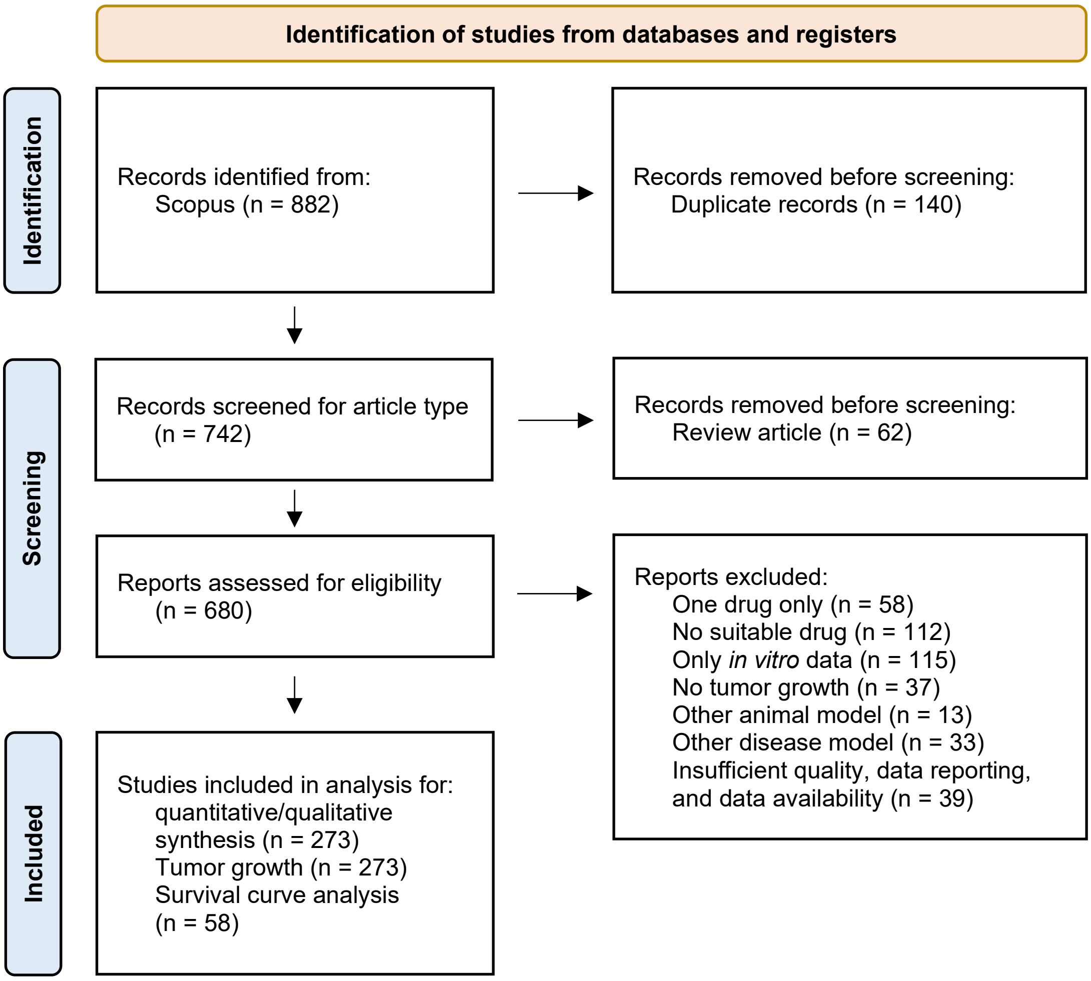

Supplementary Figure 1 | **PRISMA Flow diagram of the literature screening.** For each screening phase the number of included or excluded publications and reasons for exclusion is displayed.

Supplementary Figure 2

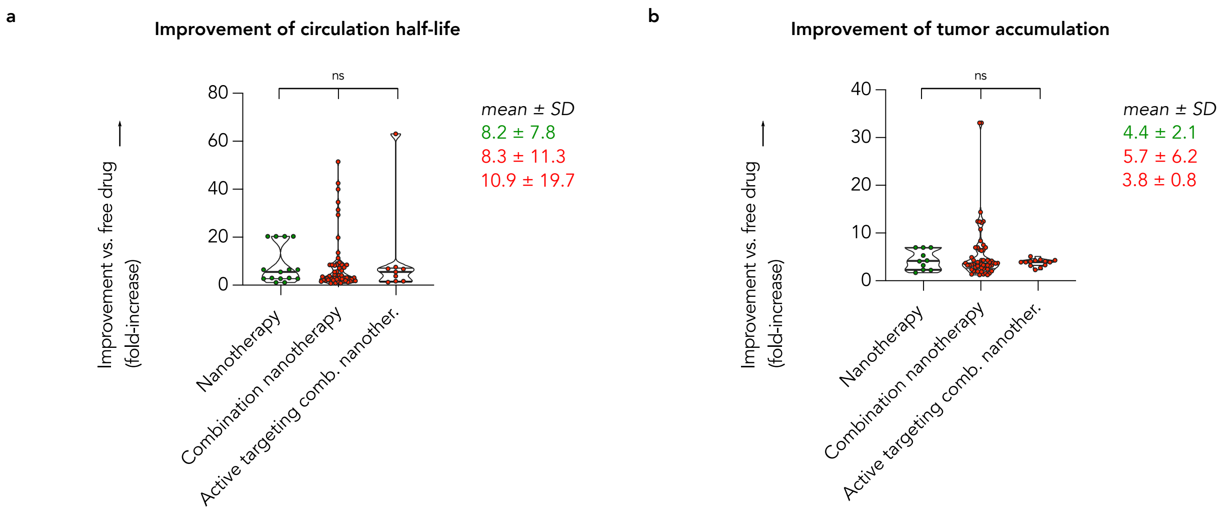

Supplementary Figure 2 | **Passive and active targeting strategies improve the circulation time and tumor accumulation of drugs. a** | In therapy as well as combination therapy, nanocarriers increase the half-life of drug molecules by 9-fold. **b** | Nanocarriers enhance drug accumulation of free drugs into the tumor by 5-fold, in combination as well as single drug therapy. Statistical significance was assessed via a two-sided Kruskal-Wallis test with Dunn's correction for multiple comparisons (**** P < .0001, *** P < .001, ** P < .01, * P < .05, ns: not significant).

Supplementary Figure 3

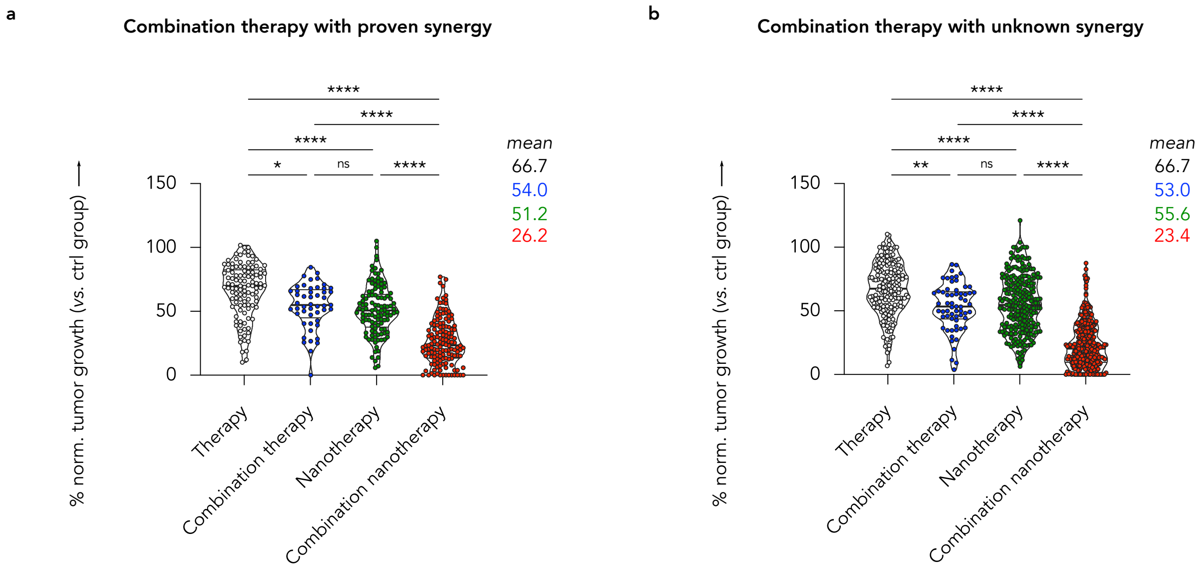

Supplementary Figure 3 | **Efficacy analysis of multidrug cancer nanotherapy with (a) proven or (b) unknown synergy relations.** The grade of response is similar in both categories. Multidrug nanomedicines outperform the other treatment groups in studies with and without proven synergy. Statistical significance was assessed via a two-sided Kruskal-Wallis test with Dunn's correction for multiple comparisons (**** $P < .0001$, *** $P < .001$, ** $P < .01$, * $P < .05$, ns: not significant).

Supplementary Figure 4

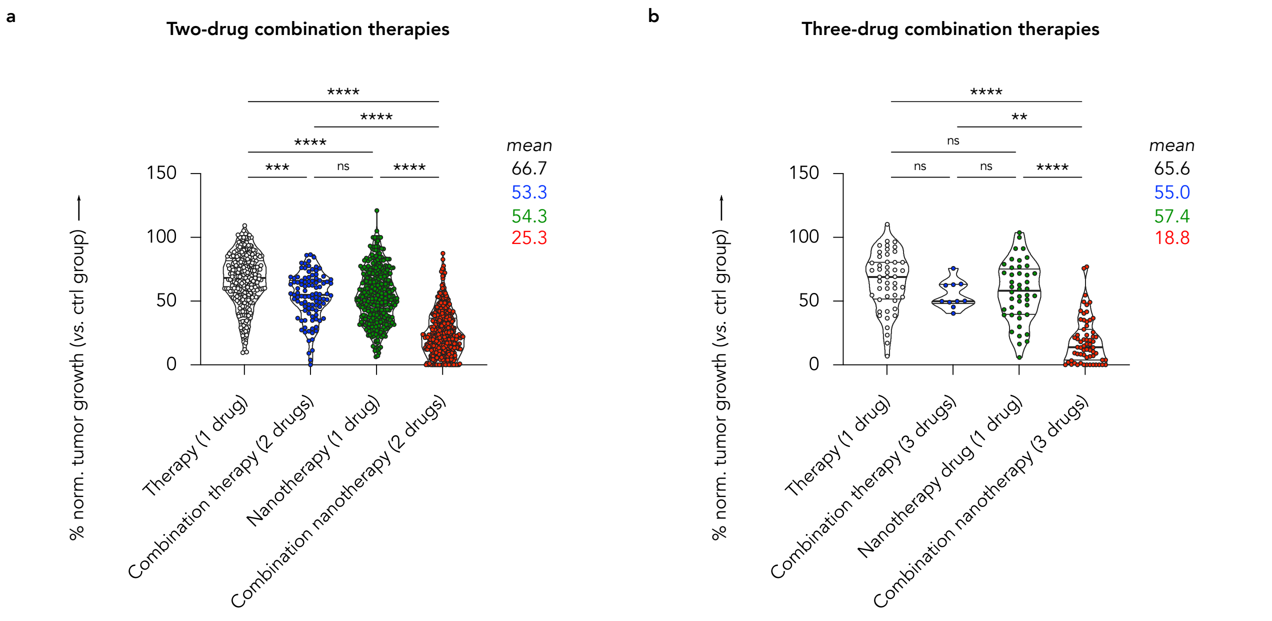

Supplementary Figure 4 | **Efficacy analysis of multidrug cancer nanotherapy in (a) two- and (b) three-drug combinations.** For both two- and three-drug formulations, combination nanotherapies outperform the other treatment groups. Three drug combinations provide an additional 6.5% reduction as compared to two drug combinations. Statistical significance was assessed via a two-sided Kruskal-Wallis test with Dunn's correction for multiple comparisons (**** P < .0001, *** P < .001, ** P < .01, * P < .05, ns: not significant).

Supplementary Figure 5

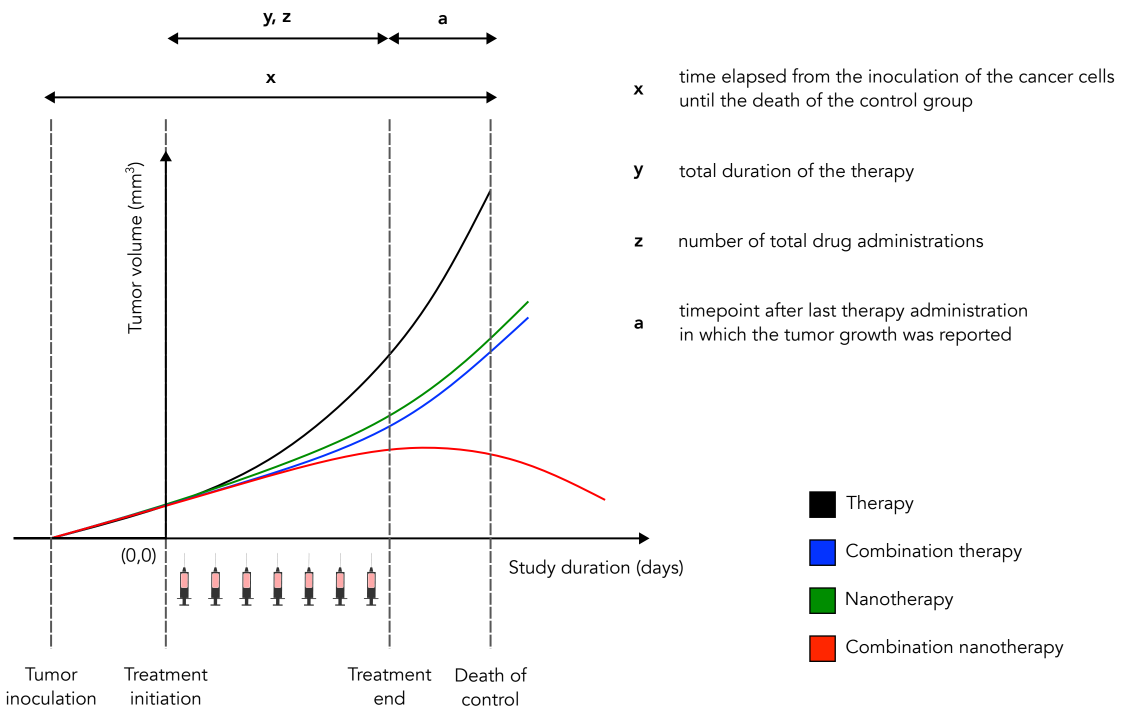

Supplementary Figure 5 | **Schematic presentation of the design of therapy study results.** Tumor inoculation starts days to weeks before therapy initiation. At day 0, therapy is initiated, resulting in a therapy duration of y days, including z numbers of total drug administrations. For all analysis performed in our study, tumor sizes were extracted at the day of the death of the control groups, to enable normalization of all therapy arms with the control groups. Variations in this study design, with respect to treatment time or study duration, may affect the readouts of our analysis. To address this, the extracted studies were fractioned according to (i) therapy duration of y days, (ii) number of total therapy administrations z, (iii) reported tumor growth at different timepoints (a days) post last therapy administration and (iv) time elapsed from the inoculation of the cancer cells until the death of the control group which amounts to x days.

Supplementary Figure 6

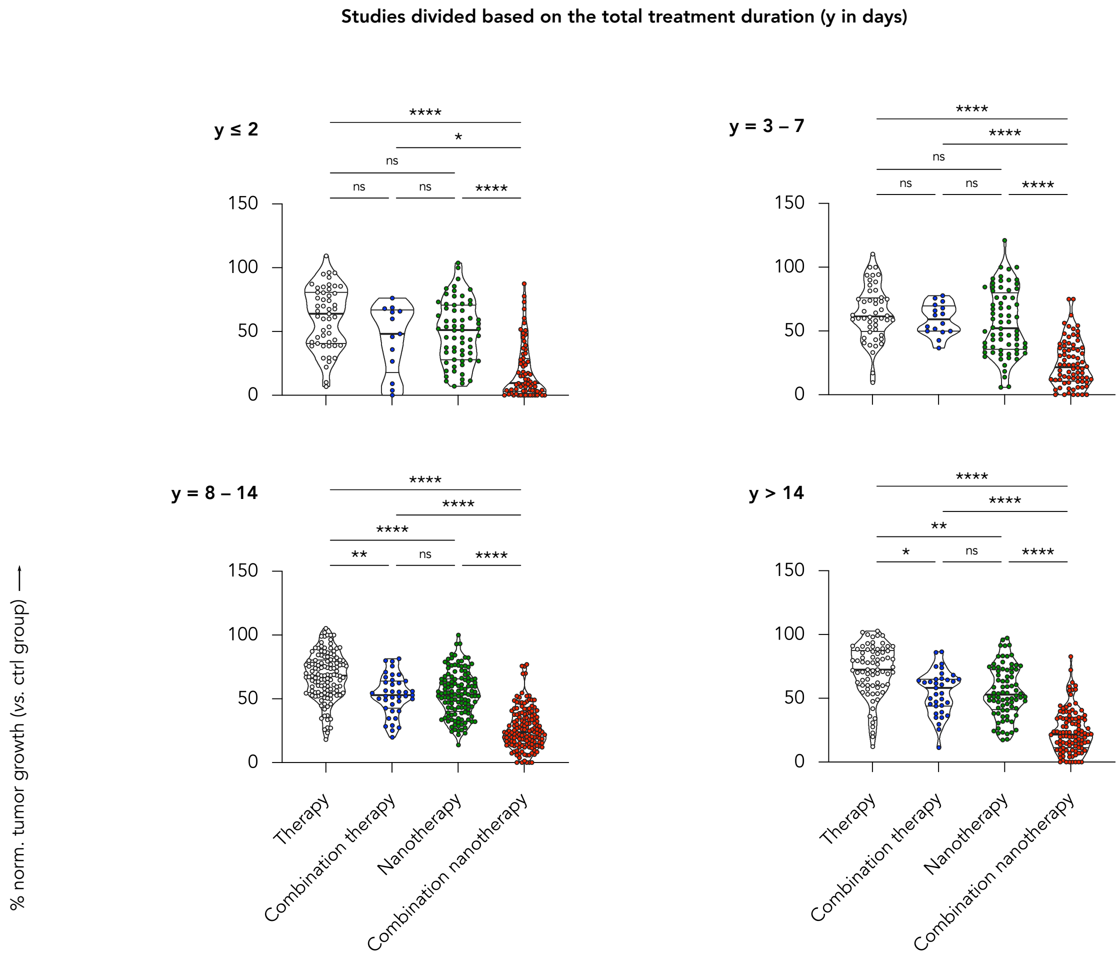

Supplementary Figure 6 | **Efficacy analysis of multidrug cancer nanotherapy fractioned by total therapy duration.** Multidrug nanomedicines outperform the other treatment groups in all fractions, showcasing the efficacy of combination nanotherapy to be independent of these classifications. Statistical significance was assessed via a two-sided Kruskal-Wallis test with Dunn's correction for multiple comparisons (**** P < .0001, *** P < .001, ** P < .01, * P < .05, ns: not significant).

Supplementary Figure 7

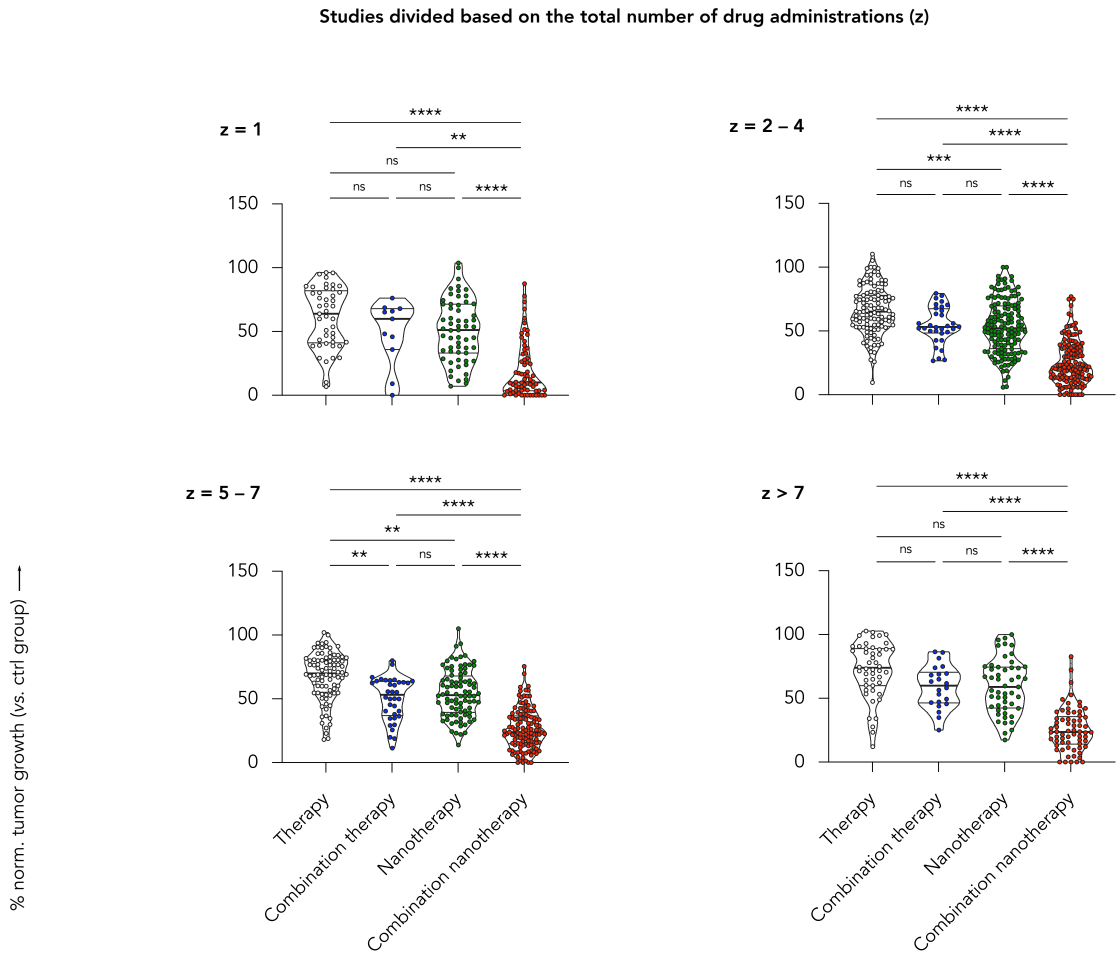

Supplementary Figure 7 | **Efficacy analysis of multidrug cancer nanotherapy fractioned by number of total therapy administrations.** Multidrug nanomedicines outperform all other treatment groups, proving the efficacy of combination nanotherapy to be independent of these classifications. Statistical significance was assessed via a two-sided Kruskal-Wallis test with Dunn's correction for multiple comparisons (**** P < .0001, *** P < .001, ** P < .01, * P < .05, ns: not significant).

Supplementary Figure 8

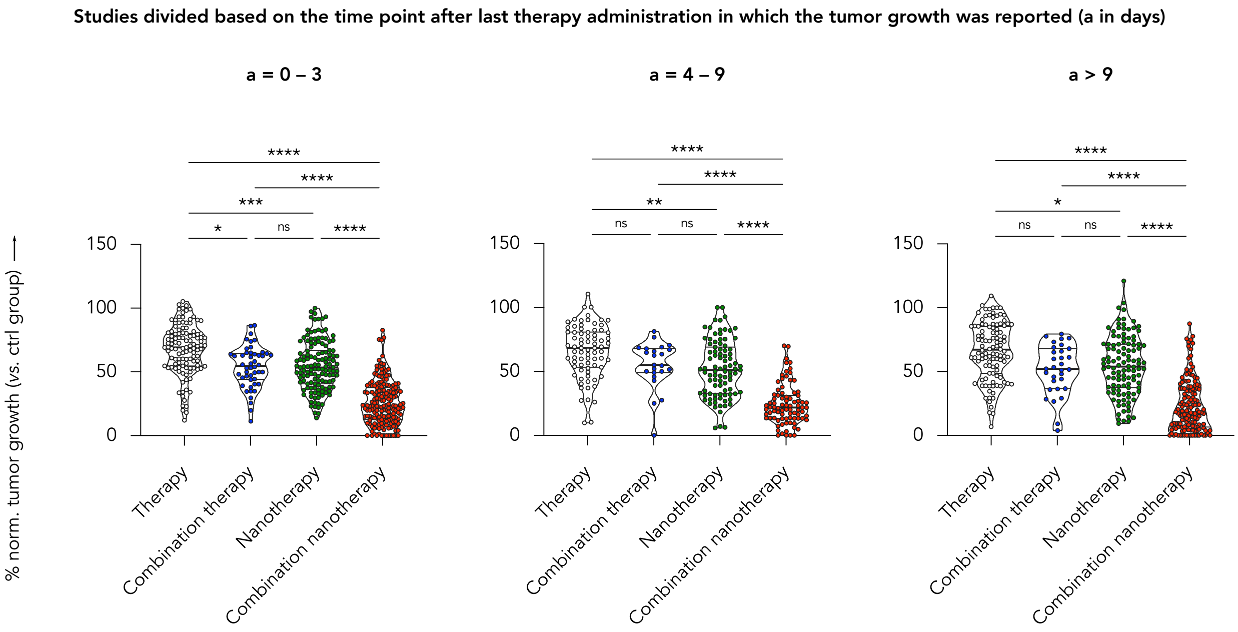

Supplementary Figure 8 | **Efficacy analysis of multidrug cancer nanotherapy fractioned by reported tumor growth at different timepoints post last therapy administration.** Multidrug nanomedicines outperform the other treatment groups in all subdivisions, proving the efficacy of combination nanotherapy to be independent of these classifications. Statistical significance was assessed via a two-sided Kruskal-Wallis test with Dunn's correction for multiple comparisons (**** $P < .0001$, *** $P < .001$, ** $P < .01$, * $P < .05$, ns: not significant).

Supplementary Figure 9

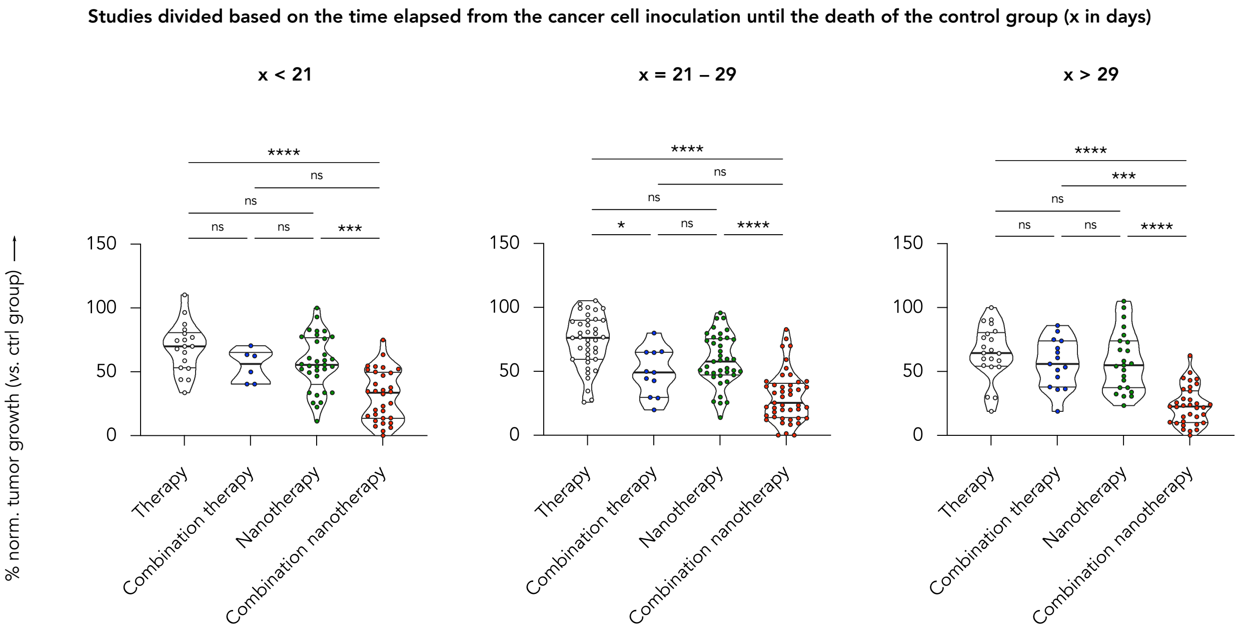

Supplementary Figure 9 | **Efficacy analysis of multidrug cancer nanotherapy fractioned by time elapsed from tumor cell inoculation until death of the control group.** Multidrug nanomedicines outperform the other treatment groups in all subdivisions, proving the efficacy of combination nanotherapy to be independent of these classifications. Statistical significance was assessed via a two-sided Kruskal-Wallis test with Dunn's correction for multiple comparisons (**** P < .0001, *** P < .001, ** P < .01, * P < .05, ns: not significant).

Supplementary Figure 10

a

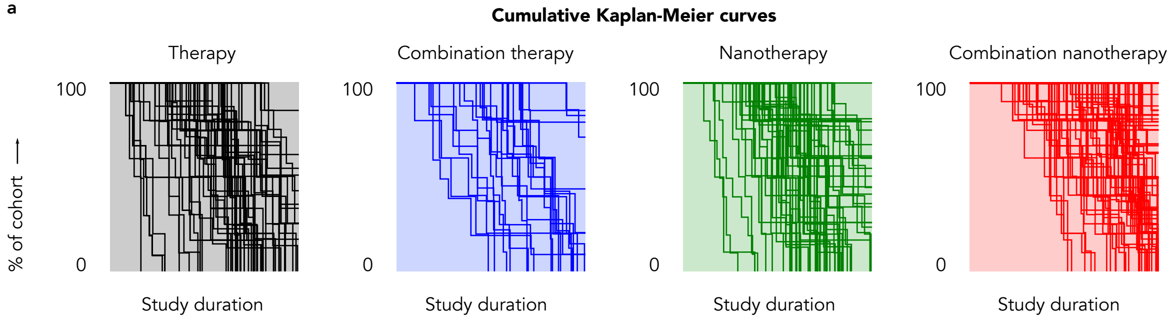

b

Descriptive statistics of Kaplan-Meier curves	Therapy	Combination therapy	Nanotherapy	Combination nanotherapy
Absolute number of Kaplan-Meier curves	59	24	64	87
Percentage of cases with full survival of the cohort by the end of the experiment	0%	0%	2%	16%
Percentage of cases with partial survival of the cohort by the end of the experiment	20%	37%	26%	40%
Percentage of cases without survival of the cohort by the end of the experiment	80%	63%	72%	44%

Supplementary Figure 10 | **Survival analysis upon multidrug cancer nanotherapy. a** | Kaplan-Meier plots were extracted from each study containing survival data. Curves were internally normalized for each study, accounting for the specific duration of each, and then adjusted to fit within a defined quadratic area. Fusing all curves reveals that multidrug nanotherapy potently prolongs survival. **b** | A thorough analysis of the results, reveals that in 87 cases of combination nanotherapy, 16% of the entire cohort achieved complete survival by the end of the experiment, in contrast to just 2% in the single drug nanotherapy group. For both single free drug treatment and free drug combination therapy, long-term survival was 0%.

Supplementary Figure 11

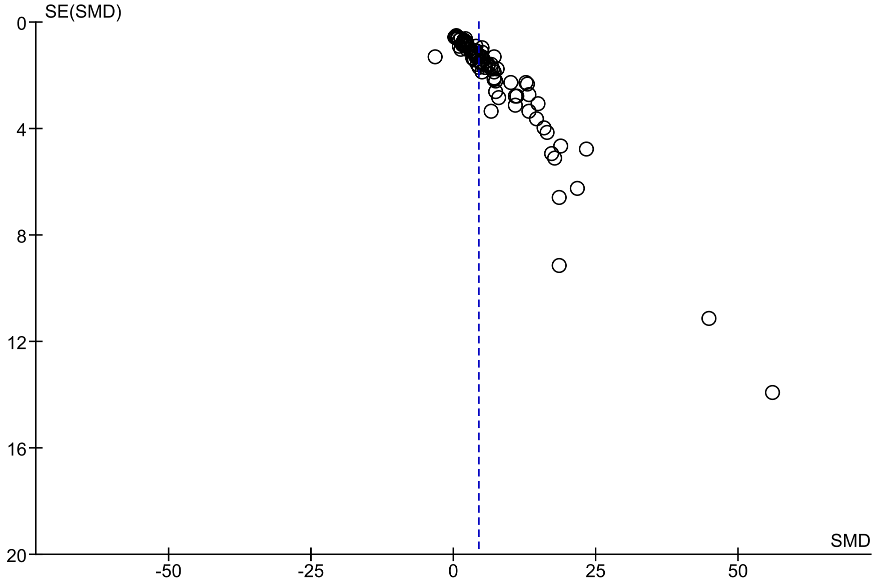

Supplementary Figure 11 | **Funnel plot of standard mean difference (SMD) between combination and nano combination therapy versus standard error (SE).** Most studies that are clustering around the true SMD, show low SE, which indicates strong consistent evidence from high precision studies. Asymmetry in the funnel plot, with scattered points around high SMD on the right side might arise from between-study heterogeneity (e.g., true differences in studies due to variance in study design, population and intervention).

Supplementary Figure 12

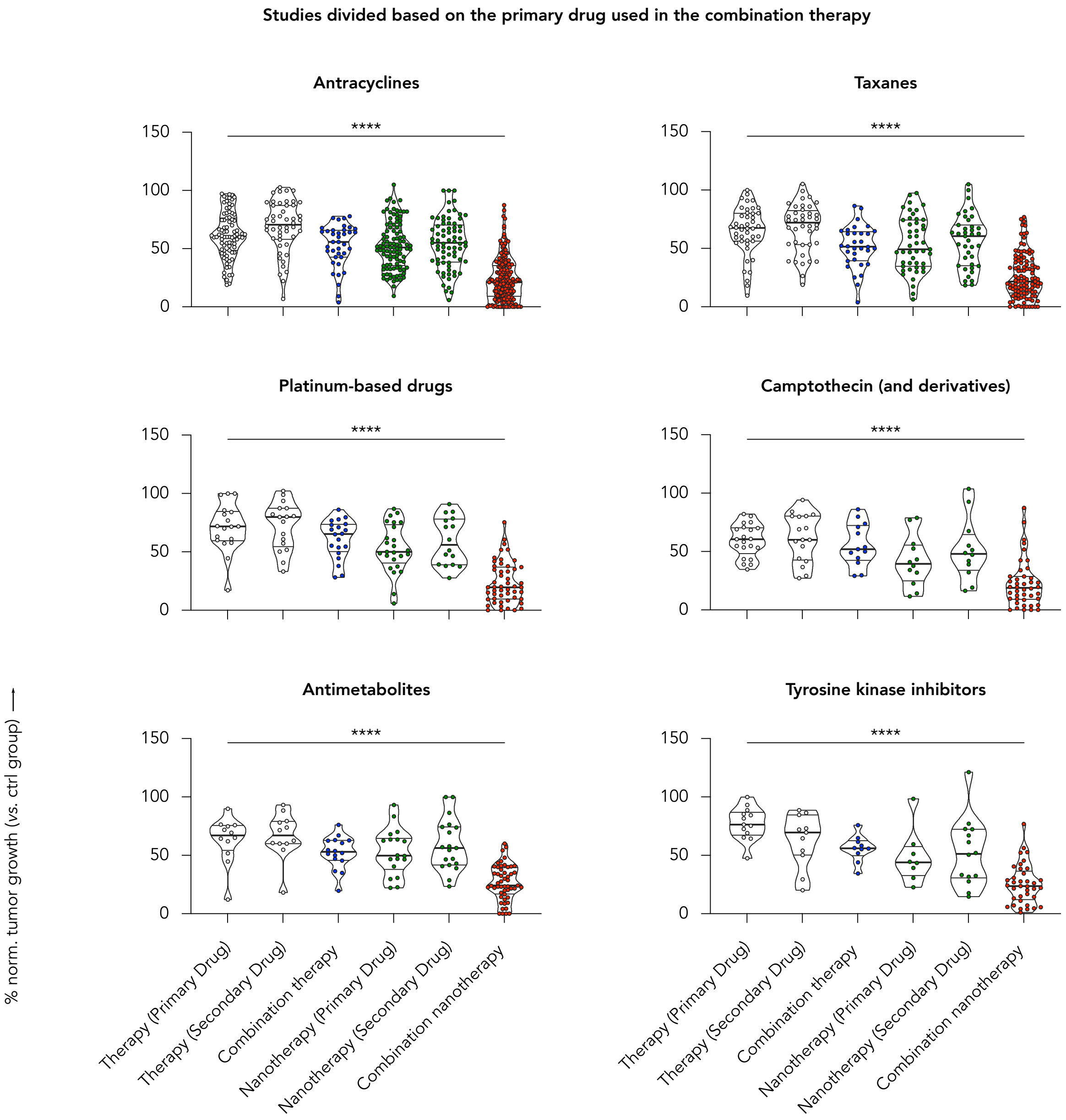

Supplementary Figure 12 | **Efficacy analysis of multidrug cancer nanotherapy based on drug combinations containing at least one anthracycline, taxane, platinum-based drug, camptothecin-derivative, antimetabolite or tyrosine kinase inhibitor.** For all drug combinations, multidrug nanomedicine results in the best tumor growth inhibition. Statistical significance was assessed via a two-sided Kruskal-Wallis test with Dunn's correction for multiple comparisons (**** P < .0001, *** P < .001, ** P < .01, * P < .05, ns: not significant).

Supplementary Table 3 | **Inclusion and exclusion criteria for the analysis.**

		Inclusion criteria	Exclusion criteria
1	Publication type	Research articles, peer reviewed	Review articles
2	Type of animals	Mouse	Other animal models
3	Type of intervention	Combination therapy with at least two drugs; at least one drug with known anticancer efficacy, and at least two drugs loadable in nanoparticles	One drug only, nanoparticle is the second “drug”, no drugs with known anticancer efficacy
4	Outcome measures	Performed anticancer efficacy study, tumor growth curves, quantitative data	Only in vitro data available, qualitative tumor growth inhibition (e.g., images of tumor bearing mice)
5	Study design	Tumor size expressed quantitatively (volume, relative volume, etc.)	Only tumor weight or number of counted lesions is provided
6	Comparator	Control group available (treated with PBS, NaCl, vehicle)	No control group available

Supplementary Table 4 | **Reporting of information in line with the PRISMA guidelines.**

No	Item	Explanation
**Title**		
1	Title	"Analysis of Multidrug Cancer Nanomedicine"
**Abstract**		
2	Abstract	"Multidrug nanomedicine is gaining momentum for co-delivering more than one drug to the same site at the same time. Our analysis of 273 pre-clinical tumour growth inhibition studies shows that multidrug nanotherapy outperforms single drug therapy, multidrug combination therapy, and single drug nanotherapy by 43, 29, and 30%, respectively. Combination nanotherapy also results in the best overall survival, with 56% of studies demonstrating complete or partial survival, versus 20-38% for control regimens. Within the multidrug nanomedicine groups, we furthermore analyzed the effect of (co-) administration schedule and strategy, passive versus active targeting, nanocarrier material, and type of therapeutic agent. Most importantly, it was found that co-encapsulating two different drugs in the same nanoformulation reduces tumor growth by an additional 19% compared to the combination of two individually encapsulated nanomedicines. We finally show that the benefit of multidrug nanotherapy is consistently observed across different cancer types, in sensitive and resistant tumors, and in xenograft and allograft models. Altogether, this meta-analysis substantiates the value of multidrug nanomedicine as a potent strategy to improve cancer therapy."
	**Introduction**	
3	Rationale	"Despite the widely anticipated advantages of nanomedicine-based combination therapy, its benefits have not yet been analyzed in a comprehensive and quantifyable manner."
4	Objectives	"Here, we therefore set out to systematically study the value of multidrug cancer nanomedicine combination therapy as compared to free drugs, free drug combinations, and single nanodrug therapy in preclinical mouse models. […]. In addition, we compared the efficacy of dual-drug nanomedicine co-formulation versus two single-drug nanomedicines. For comprehensive understanding, we finally also analyzed the impact of treatment schedule, tumor type, drug resistance, immunological status, and targeting strategy on the added value of multidrug cancer nanomedicine combination therapy".
	**Methods**	
5	Eligibility criteria	Manuscripts in English language. (i) peer-reviewed research article of a primary study (ii) containing *in vivo* quantitative therapy data from preclinical murine models, (iii) focusing on oncological disorders, (iv) containing at least one pharmacologically active small drug molecule with proven anti-cancer effect, (v) containing at least two loadable drugs, i.e., cases where the nanocarrier acts as therapeutic component (e.g., photothermal therapy) were not included, and (v) presenting tumor growth curves as therapy efficacy readouts (**Table S3,4**).
6	Information sources	Scopus.com, "manuscripts published between January 2007 and December 2022".
7	Search strategy	All searches were limited to research articles. "[(i) "combination therapy" AND "nanomedicine", (ii) "combination chemotherapy" AND "nanomedicine", and (iii) ("co-delivery" OR "codelivery") AND "nanomedicine"]".
8	Selection process	After merging all search results and removing duplicate publications, the study selection was based on screening of publication title and abstract. If a publication could not be judged by its title / abstract because of insufficient or unclear information, it was included for the second selection phase. In the second selection phase, the first author performed a comprehensive full-text screening of the remaining publications for eligibility for data extraction. Conflicting results were discussed with the second and third author.

9	Data collection process	The first author conducted the initial search, screening, and selection of the studies, followed by the data extraction and digitalization. The last author confirmed the validity of the study inclusions and data extraction. Conflicting findings, although scarce, were resolved by the middle author.
10a	Data items – Outcomes	If one manuscript contained multiple data (same therapy in multiple tumor models, different combinations in the same tumor model, different concentrations or drug ratios in the same tumor model), these were considered as individual, separate datasets. As such 273 manuscripts resulted in 311 individual datasets.<br>"Tumor sizes were extracted from *in vivo* therapy studies that reported tumor sizes in the text or presented tumor growth curves as part of the figures. From tumor growth curves, numbers were extracted using the online plot digitizer graphreader (www.graphreader.com). Tumor sizes were usually indicated as absolute or relative tumor volumes (mm3), tumor volume increase (%), or tumor inhibition rate (%). These tumor sizes were compared against the control groups in the same in vivo therapy studies, mostly encompassing mice injected with PBS or vehicles. Absolute tumor values from each therapy study were then compared with the control group growth. In each individual study, the latter was normalized as "100%" tumor growth, and the values of the various treatment groups were expressed as % of normalized tumor growth. The therapies injected to mice were categorized in four groups, namely therapy (single free-drug), combination therapy (free-drug combination), nanotherapy (single drug nanocarrier), and finally, combination nanotherapy (multidrug nanocarriers, combination of single-drug nanocarriers, or mixtures of free and nanodrugs). The single and combination nanotherapy categories were further sub-divided depending on the focus of different analysis (e.g., passive vs. active targeting, nanomaterial comparison, etc.)."
10b	Data items – Other variables	The qualitative data extraction from each manuscript contains relevant information for (i) study identification, e.g., study title, (ii) study design, e.g., used drugs, nanoparticle type, delivery strategy, tumor model, (iii) anti-tumor efficacy study, e.g., treatment duration, administration times, and (iv) treatment groups, e.g., free drugs, free drug combination, one drug nanotherapy, active targeting one drug nanotherapy, combination nanotherapy, active targeting combination nanotherapy etc.
11	Study risk of bias assessment	All studies derived upon applying the exclusion criteria were included in the final analysis. No study was excluded after this stage. Hence, even studies with negative results were included in the analysis.
12	Effect measures	Comparative outcome data between groups were received through statistical analysis, using a two-sided Kruskal-Wallis test with Dunn's correction for multiple comparisons, or a two-tailed Mann-Whitney test, and expressed as individual *P*-values.<br>"Absolute tumor values from each therapy study were then compared with the control group growth. In each individual study, the latter was normalized as "100%" tumor growth, and the values of the various treatment groups were expressed as % of normalized tumor growth."<br>For the meta-analysis performed in **Fig. 2c**: "Values were entered into a systematic review software (Review Manager, Version 5.4.1, Cochrane Collaboration 2022) to perform a meta-analysis using an inverse variance and random effects model. The standardized mean difference in effect between combination therapy and combination nanotherapy is expressed in numbers or depicted graphically as a forest plot. Heterogeneity between the groups was investigated using Chi^2 tests, the I^2 index and was visually presented via a funnel plot, displaying standard mean difference vs standard error."
13a	Synthesis methods – Eligibility for synthesis	Given the complexity and individuality of the study designs, multiple categorizations were attempted to differentiate between (i) different drug categories, (ii) delivery strategies used (passive vs active targeting, co-delivery vs separate delivery, different nanomaterials), (iii) tumor models used (sensitive vs resistant, xenograft vs allograft, tumor growth kinetics), (iv) general execution of experiments (treatment duration, administration times).<br>"The single and combination nanotherapy categories were further sub-divided depending on the focus of different analysis (e.g., passive vs. active targeting, nanomaterial comparison, etc.).

13b	Synthesis methods – Preparing for synthesis	For the synthesis of tumor growth inhibition, extracted absolute tumor sizes were compared to control groups. The latter was then normalized as “100%” tumor growth, and the values of the various treatment groups were expressed as “% normalized tumor growth”.
13c	Synthesis methods – Tabulation and graphical methods	“Numbers were entered as replicate values in GraphPad Prism 9 and graphed as violin plots for depicting the mean, range, and 25%, 50% and 75% percentiles. Statistical significance was determined by a Kruskal-Wallis test with Dunn’s correction for multiple comparisons, or a Mann-Whitney test (*** P < .0001, *** P < .001, ** P < .01, * P < .05, ns: not significant). Descriptive statistics are displayed in **Table S5-55**.”<br>For assessing the effect of combination nanotherapy, nanotherapy, combination therapy and therapy, on overall survival, Kaplan-Meier survival curves were normalized for each study internally, taking into consideration the individual study duration, and they were rearranged to fit in a defined quadratic area. The analysis was done using Inkscape Project (2020).
13d	Synthesis methods – statistical synthesis methods	“For the forest plot analysis, non-normalized tumor sizes, standard deviations, and animal group size from mice treated with combination therapy (free-drug combination) vs. combination nanotherapy were extracted from tumor growth curves. Values were entered into a systematic review software (Review Manager, Version 5.4.1, Cochrane Collaboration 2022) to perform a meta-analysis using an inverse variance and random effects model. The standardized mean difference in effect between combination therapy and combination nanotherapy is expressed in numbers or depicted graphically as a forest plot. Heterogeneity between the groups was investigated using Chi^2 tests and the I^2 index.”<br>Inverse Variance Weighting ensures that studies are weighted according to their precision, leading to more reliable and efficient estimates. The Random Effects Model provides a framework to incorporate and account for heterogeneity among studies, resulting in more generalizable and realistic effect size estimates. Together, they form a robust approach for synthesizing evidence in meta-analyses, balancing precision and variability to derive meaningful conclusions from diverse study results.<br>For the remaining figures, “Statistical significance was determined by a two-sided Kruskal-Wallis test with Dunn’s correction for multiple comparisons, or a two-tailed Mann-Whitney test (**** P < .0001, *** P < .001, ** P < .01, * P < .05, ns: not significant).”
13e	Synthesis methods – methods to explore heterogeneity	For the meta-analysis conducted and displayed in **Fig. 2c**, “heterogeneity between the groups was investigated using Chi^2 tests and the I^2 index”, which expresses the percentage variability of the results related to heterogeneity rather than to the sampling error.
13f	Synthesis methods – sensitivity analyses	Not applicable for our analysis.
14	Reporting bias assessment	A funnel plot was generated to assess bias and heterogeneity.
15	Certainty assessment	“Statistical significance was determined by a two-sided Kruskal-Wallis test with Dunn’s correction for multiple comparisons or a two-tailed Mann-Whitney test (**** P < .0001, *** P < .001, ** P < .01, * P < .05, ns: not significant).”
	**Results**	
16a	Study selection – Flow of studies	“All available research papers on combination nanotherapy were collected via a thorough literature search in the scopus.com database. Via this search, three groups of relevant keywords were selected (**Fig. 1a**), resulting in 882 results addressing multidrug cancer nanomedicine combination therapy. Upon applying relevant exclusion criteria, a total of 273 suitable manuscripts (which are provided as supplementary material, **Table S1**) were selected for in-depth analysis (**Fig. 1b**)”.
16b	Study selection – Excluded studies	Studies were carefully considered for inclusion according to the criteria listed above. All final 273 met those criteria and were therefore eligible for data synthesis.

17	Study characteristics	See **Table S1**.
18	Risk of bias in studies	n/a
19	Results of individual studies	**Fig. 2c** presents a summary of the effect estimate of all included comparisons, **Table S2** presents effect estimates of each individual study. For **Fig. 2-5** as well as **Fig. S2-4, Fig. S6-9** and **Fig. S12**, numbers are entered as replicate values, and their distribution is displayed in violin plots. Descriptive statistics are summarized in **Tables S5-55**.
20	Results of syntheses	Summary of meta-analysis results are found in **Fig. 2-5** and in **Table S2,** and **Tables S5-55**.
21	Risk of reporting biases in syntheses	n/a
22	Certainty of evidence	n/a
**Discussion**		
23a	Interpretation	"This meta-analysis demonstrates a statistically significant benefit of combination anticancer nanotherapy over free drug combination therapy in preclinical mouse models (**Fig. 2**). One clear reason for this beneficial outcome is undoubtfully the ability of nanocarriers to improve the circulation time and target site accumulation of drug molecules (**Fig. S2**). This benefit was consistently observed across different tumor types and experimental settings (**Fig. 3-5, Fig. S3-9; Fig. S12**). In addition, arguably most importantly, our meta-analysis showcases that co-loading two different drugs within the same nanomedicine formulation is statistically superior over the co-administration of two single-drug-loaded nanomedicines (**Fig. 3a,b**). These findings corroborate ongoing translational efforts towards expanding the use of combinatorial nanomedicines for multidrug delivery."
23b	Limitations of evidence	Any publication bias is transferred equally to both single-drug nanotherapy and multi-drug nanotherapy groups. Studies that showed a worse efficacy of multi-drug nanomedicines in comparison to other regimes were also included in the analysis. From our analysis in 4 cases monotherapy performed better than combination therapy, and 17 cases were combination nanotherapy was less effective than single-nanotherapy.<br>"The outcome of our meta-analysis should be interpreted with care. Publication bias is a factor that should not be overlooked, as many researchers opt out from publishing negative results. In most cases, we found that combination therapy is better than monotherapy, for both free drug and for nanodrug treatments. However, we also identified four cases where monotherapy performed better than combination therapy, and 17 cases where combination nanotherapy was less effective than single-drug nanotherapy. Nonetheless, this issue is evident even in studies focusing on single-drug nanotherapies, where researchers may only publish positive results regarding the anticancer efficacy of a single-drug nanomedicine intervention. Consequently, publication bias seems to affect both the single-drug and the multi-drug nanotherapy groups equally in our comparative analysis."
23c	Limitations of review processes	Use of one database, authors were not contacted for clarifying missing information.
23d	Implications	"When translating results from mice to humans, it is important to appreciate the significant interspecies differences, especially in terms of nanoparticle circulation half-life in blood. Mice often have faster metabolism rates and shorter circulation times as compared to humans. Thus, nanoparticles may be cleared faster from the blood in mice, potentially leading to discrepancies in their efficacy benefits when translating findings to human applications. Advanced studies involving non-human primates may better inform pharmacokinetic assumptions about human responses.<br>To move the field forward and ensure translational impact, it is important to carefully consider in which cases multidrug delivery is helpful and needed, versus situations in which it conceptually does not make much sense. For cancer chemotherapy, several scenarios can be envisaged in which multidrug delivery adds value. Co-delivering two different cytotoxic

drugs to and into the same target cell at a synergistic ratio is viable and valuable, as evidenced by the successful product development and clinical approval of the frontrunner double-drug nanoformulation Vyxeos®. This non-pegylated liposome, which contains daunorubicin and cytarabine in a 5:1 ratio, efficiently targets and kills leukemia cells upon i.v. administration, and it creates significant added value for patients, with an improvement in median overall survival from 6 months for daunorubicin and cytarabine in free form to 10 months for Vyxeos® (HR=0.69; P=0.003). When co-administered in free form, because of the different physicochemical and pharmacokinetic parameters of daunorubicin and cytarabine, it seems chanceless to achieve synergistic ratios in target cells, even if i.v. administered at a 5:1 dose. If two single drug-loaded liposomes would be administered at a 5:1 dose, the question is how many of those would eventually end up in that ideal ratio in the same leukemia target cell in the blood stream or in the bone marrow. For three-drug chemotherapy combinations, mathematical modelling has shown that chances are >10 higher to achieve synergistic drug ratios in target cells when co-formulating drugs in nanomedicines vs. co-administering single-drug nanomedicines. For double-drug formulations, the mathematical chances will be somewhat lower, but certainly still high enough to produce a significant and clinically meaningful increase in therapeutic outcome.

When transiting from hematological to solid cancers, the multidrug delivery situation becomes very different. In the case of hematological malignancies, like leukemia, liposomes have reasonably good access to with leukemia cells in the blood stream and bone marrow, particularly if they are not pegylated (as in the case of Vyxeos®). To optimally reach non-hematological solid tumors and metastases, nanomedicines are typically pegylated, as this increases circulation times and tumor concentrations. However, pegylation will also skew the uptake of nanoparticles away from cancer cells, towards tumor-associated macrophages. For multidrug delivery, this means that synergistic ratios are potentially delivered to the tumor compartment as a whole, but not to individual tumor cells, where pharmacological synergy is needed the most. Nonetheless, the outcomes of our meta-analysis show that even in the case of solid tumor targeting, nanomedicine co-delivery is superior over co-administration of two single drug-loaded nanomedicines (**Fig. 3a,b**). In line with the above reasoning on the importance of cancer cell uptake vs. tumor-associated macrophage uptake of multidrug-loaded nanomedicines, we found no statistically significant added value of pegylation on therapeutic performance (**Fig. 3c**). Also, in agreement with this, we did find a statistically significant added value of active over passive targeting in case of nanomedicine combination therapy, which was not observed in case of active vs. passive targeting with single-drug-loaded nanomedicines (**Fig. 3d**). It is important to keep in mind in this regard that active targeting typically only improves the balance between cancer cell vs. macrophage uptake, and not the overall levels of nanomedicine tumor accumulation. Accordingly, in the case of multidrug nanomedicine, where transportation of synergistic amounts of chemotherapeutic drugs to and into cancer cells is crucial, active targeting is identified here as an important enabler.

The added value of multidrug cancer nanomedicine crucially depends on the mechanism of action of the agents that are being co-delivered. When combining a classical chemotherapy drug with a tumor microenvironment-modulating or immune-activating agent, it seems unnecessary to co-formulate both agents in the same nanoparticle. There may be intellectual property-related reasons or other translationally relevant arguments for doing so (e.g., having to perform toxicology studies and phase I trials for just one double-drug nanoformulation vs. for two single-drug nanomedicines), but from a pure pharmacological point of view, there does not seem to be much added value in co-loading agents that do not target the same cell. Scenarios can be envisaged in which there can be benefit, e.g., when co-delivering a vascular disrupting agent together with a chemotherapy drug in a nanoparticle that enables temporally controlled release kinetics, with the former agent being released first, to cause tumor vascular shutdown, and the latter agent being released afterwards, to restrict cytotoxicity effects to the tumor compartment and attenuate systemic side effects.

Conceptually and scientifically, multidrug nanomedicine therapies are elegant and appealing. The possibility of being able to co-deliver more than one active pharmaceutical ingredient to the same site or cell in the body at the same point in time opens up many therapeutic opportunities. These go way beyond cancer, encompassing e.g., also the co-delivery of 3-4 antiviral drugs for HIV or tuberculosis therapy, or the co-delivery of siRNAs, miRNAs, mRNAs and/or sgRNAs for optimized protein replacement therapy, gene silencing or gene editing.

		To create clinical impact, it will be crucial to align scientific and conceptual nanotechnology engineering advances with rational pharmacological mechanisms and realistic clinical scenarios. The multidrug nanomedicine field is still in its infancy, with only Vyxeos® on the market, and only a few double-drug formulations in clinical development. It is expected that in the years to come, multidrug nanomedicines will gain more and more traction. From a pharmaceutical technology and industrial development point of view, they are very appealing, as there still is a lot of intellectual property and clinical application space to claim. Based on the outcome of our meta-analysis, and on the above-discussed insights and opportunities, we conclude that multidrug nanomedicine holds significant promise for applications in oncology and beyond.”
**Other information**		
24	Registration and protocol	n/a
25	Support	“The authors gratefully acknowledge financial support by the German Research Foundation (DFG: Collaborative Research Center SFB1066; Excellence Initiative RWTH JPI 2021; Clinical Research Unit CRU344; Research Training Group RTG2375), by the European Research Council (ERC: Consolidator Grant Meta-Targeting (864121)), and by the German Cancer Aid (SDK MSSO postgraduate program).”
26	Competing interests	The authors have no competing interests to report.
27	Availability of data, code materials	5 main figures, 12 supplementary figures, 55 supplementary tables.

Supplementary Table 5 | **Descriptive statistics of studies with proven synergy drug combinations (Fig. S3a).**

Group	Therapy	Combination therapy	Nanotherapy	Combination nanotherapy
# data points	110	49	114	142
minimum	10.2	0.0	5.8	0.0
maximum	101.9	84.4	105.0	76.9
range	91.7	84.4	99.2	76.9
mean	66.7	54.0	51.2	26.2
SD	21.0	17.5	19.6	18.0
SEM	2.0	2.5	1.8	1.5
lower 95% CI of mean	62.6	49.0	47.6	23.2
upper 95% CI of mean	70.6	59.0	54.9	29.2

Supplementary Table 6 | **Descriptive statistics of studies with unknown synergy drug combinations (Fig. S3b).**

Group	Therapy	Combination therapy	Nanotherapy	Combination nanotherapy
# data points	203	62	261	296
minimum	6.8	3.9	6.4	0.0
maximum	110.3	86.4	121.2	87.4
range	103.5	82.5	114.8	87.4
mean	66.7	53.0	55.6	23.4
SD	20.6	18.4	22.2	17.8
SEM	1.5	2.3	1.4	1.0
lower 95% CI of mean	64.2	48.3	52.9	21.4
upper 95% CI of mean	69.9	57.6	58.3	25.5

Supplementary Table 7 | **Descriptive statistics of studies with two-drug combinations (Fig. S4a).**

Group	Therapy (1 drug)	Combination therapy (2 drugs)	Nanotherapy (1 drug)	Combination nanotherapy (2 drugs)
# data points	290	101	356	375
minimum	9.6	0.0	6.4	0.0
maximum	109.4	86.4	121.2	87.4
range	99.8	86.4	114.8	87.4
mean	66.7	53.3	54.3	25.3
SD	20.4	18.5	21.1	17.7
SEM	1.2	1.8	1.1	0.9
lower 95% CI of mean	64.4	49.6	52.1	23.5
upper 95% CI of mean	69.1	56.9	56.5	27.1

Supplementary Table 8 | **Descriptive statistics of studies with three-drug combinations (Fig. S4b).**

Group	Therapy (1 drug)	Combination therapy (3 drugs)	Nanotherapy (1 drug)	Combination nanotherapy (3 drugs)
# data points	48	10	44	63
minimum	6.8	40.4	5.8	0.0
maximum	110.3	75.7	103.8	76.9
range	103.5	35.3	98.0	76.9
mean	65.6	55.0	57.4	18.8
SD	22.7	10.7	23.2	18.2
SEM	3.3	3.4	3.5	2.3
lower 95% CI of mean	59.0	47.3	50.3	14.2
upper 95% CI of mean	72.2	62.7	64.4	23.3

Supplementary Table 9 | **Descriptive statistics of studies subdivided by therapy duration (≤2 days) (Fig. S6).**

Group	Therapy	Combination therapy	Nanotherapy	Combination nanotherapy
# data points	51	13	63	72
minimum	6.8	0.0	7.1	0.0
maximum	109.4	76.3	103.8	87.4
range	102.6	76.3	96.7	87.4
mean	61.0	44.2	49.7	18.9
SD	24.3	26.7	24.4	21.6
SEM	3.4	7.4	3.1	2.6
lower 95% CI of mean	54.2	28.0	43.6	13.8
upper 95% CI of mean	67.9	60.3	55.9	23.9

Supplementary Table 10 | **Descriptive statistics of studies subdivided by therapy duration (3-7 days) (Fig. S6).**

Group	Therapy	Combination therapy	Nanotherapy	Combination nanotherapy
# data points	48	16	68	76
minimum	9.6	36.7	5.8	0.0
maximum	110.3	77.8	121.2	75.0
range	100.7	41.1	115.4	75.0
mean	64.0	59.2	56.7	24.8
SD	21.4	12.2	25.7	18.2
SEM	3.1	3.1	3.1	2.1
lower 95% CI of mean	57.8	52.7	50.5	20.6
upper 95% CI of mean	70.2	65.7	63.0	28.9

Supplementary Table 11 | **Descriptive statistics of studies subdivided by therapy duration (8-14 days) (Fig. S6).**

Group	Therapy	Combination therapy	Nanotherapy	Combination nanotherapy
# data points	126	39	128	150
minimum	18.2	19.8	14.0	0.0
maximum	105.3	81.4	100.0	76.9
range	87.1	61.6	86.0	76.9
mean	67.9	53.0	54.0	26.8
SD	18.4	15.6	17.6	15.9
SEM	1.6	2.5	1.6	1.3
lower 95% CI of mean	64.7	48.0	51.0	24.2
upper 95% CI of mean	71.2	58.1	57.1	29.3

Supplementary Table 12 | **Descriptive statistics of studies subdivided by therapy duration (>14 days) (Fig. S6).**

Group	Therapy	Combination therapy	Nanotherapy	Combination nanotherapy
# data points	74	35	80	106
minimum	12.2	11.4	17.5	0.0
maximum	102.7	86.4	97.4	82.8
range	90.5	75.0	79.9	82.8
mean	71.2	54.7	55.2	24.4
SD	20.6	17.1	20.0	17.0
SEM	2.4	2.9	2.2	1.7
lower 95% CI of mean	66.4	48.8	50.8	21.1
upper 95% CI of mean	75.9	60.6	59.7	27.7

Supplementary Table 13 | **Descriptive statistics of studies applying a single treatment administration (Fig. S7).**

Group	Therapy	Combination therapy	Nanotherapy	Combination nanotherapy
# data points	45	11	59	68
minimum	6.8	0.0	7.1	0.0
maximum	96.4	76.3	103.8	87.4
range	89.6	76.3	96.7	87.4
mean	61.0	49.4	50.7	19.7
SD	23.9	25.2	24.2	21.8
SEM	3.6	7.6	3.1	2.6
lower 95% CI of mean	53.8	32.5	44.4	14.4
upper 95% CI of mean	68.2	66.3	57.0	25.0

Supplementary Table 14 | **Descriptive statistics of studies applying 2-4 treatment administrations (Fig. S7).**

Group	Therapy	Combination therapy	Nanotherapy	Combination nanotherapy
# data points	111	32	149	147
minimum	9.6	26.7	5.8	0.0
maximum	110.3	79.6	100.0	76.9
range	100.7	52.9	94.2	76.9
mean	66.2	54.6	53.5	25.0
SD	18.9	14.5	20.9	17.3
SEM	1.8	2.6	1.7	1.4
lower 95% CI of mean	62.7	49.4	50.1	22.2
upper 95% CI of mean	69.8	59.8	56.9	27.8

Supplementary Table 15 | **Descriptive statistics of studies applying 5-7 treatment administrations (Fig. S7).**

Group	Therapy	Combination therapy	Nanotherapy	Combination nanotherapy
# data points	88	36	88	123
minimum	18.2	11.4	14.0	0.0
maximum	102.1	80.0	105.0	75.4
range	83.9	68.6	91.0	75.4
mean	67.8	50.1	54.5	26.6
SD	18.9	16.9	18.6	16.0
SEM	2.0	2.8	2.0	1.4
lower 95% CI of mean	63.7	44.3	50.5	23.7
upper 95% CI of mean	71.8	55.8	58.4	29.4

Supplementary Table 16 | **Descriptive statistics of studies applying >7 treatment administrations (Fig. S7).**

Group	Therapy	Combination therapy	Nanotherapy	Combination nanotherapy
# data points	48	22	51	66
minimum	12.2	25.1	17.5	0.0
maximum	102.7	86.4	100.0	82.8
range	90.5	61.3	82.5	82.8
mean	72.3	59.1	59.3	25.7
SD	21.8	16.6	21.1	17.0
SEM	3.2	3.5	2.9	2.1
lower 95% CI of mean	65.9	51.8	53.4	21.5
upper 95% CI of mean	78.6	66.4	65.2	29.9

Supplementary Table 17 | **Descriptive statistics of studies subdivided by the time after last drug administration (0-3 days) in which the tumor growth was reported (Fig. S8).**

Group	Therapy	Combination therapy	Nanotherapy	Combination nanotherapy
# data points	115	48	133	173
minimum	12.2	11.4	14.0	0.0
maximum	105.3	86.4	100.0	82.8
range	93.1	75.0	86.0	82.8
mean	67.6	54.0	54.7	25.9
SD	19.8	16.3	19.1	17.2
SEM	1.9	2.4	1.7	1.3
lower 95% CI of mean	63.9	49.2	51.4	23.3
upper 95% CI of mean	71.2	58.7	58.0	28.5

Supplementary Table 18 | **Descriptive statistics of studies subdivided by the time after last drug administration (4-9 days) in which the tumor growth was reported (Fig. S8).**

Group	Therapy	Combination therapy	Nanotherapy	Combination nanotherapy
# data points	76	23	91	83
minimum	9.6	0.0	5.8	0.0
maximum	110.3	81.4	100.0	70.0
range	100.7	81.4	94.2	70.0
mean	65.7	54.9	52.1	24.2
SD	20.5	18.4	22.1	15.8
SEM	2.4	3.8	2.3	1.7
lower 95% CI of mean	61.0	46.9	47.5	20.7
upper 95% CI of mean	70.4	62.9	56.7	27.6

Supplementary Table 19 | **Descriptive statistics of studies subdivided by the time after last drug administration (>9 days) in which the tumor growth was reported (Fig. S8).**

Group	Therapy	Combination therapy	Nanotherapy	Combination nanotherapy
# data points	106	31	112	146
minimum	6.8	3.9	9.5	0.0
maximum	109.4	79.6	121.2	87.4
range	102.6	75.7	111.7	87.4
mean	66.3	51.8	54.7	23.1
SD	22.5	19.5	23.3	20.1
SEM	2.2	3.5	2.2	1.7
lower 95% CI of mean	61.9	44.6	50.4	19.8
upper 95% CI of mean	70.6	58.9	59.1	26.4

Supplementary Table 20 | **Descriptive statistics of studies subdivided by the duration of the used tumor model (< 21 days) (Fig. S9).**

Group	Therapy	Combination therapy	Nanotherapy	Combination nanotherapy
# data points	18	6	33	32
minimum	33.7	40.4	11.5	0.0
maximum	110.3	70.5	100.0	75.0
range	76.6	30.1	88.5	75.0
mean	69.1	54.6	56.7	31.2
SD	19.4	12.8	21.5	19.8
SEM	4.6	5.2	3.8	3.5
lower 95% CI of mean	59.4	41.2	49.0	24.1
upper 95% CI of mean	78.7	68.0	64.3	38.3

Supplementary Table 21 | **Descriptive statistics of studies subdivided by the duration of the used tumor model (21-29 days) (Fig. S9).**

Group	Therapy	Combination therapy	Nanotherapy	Combination nanotherapy
# data points	39	11	39	45
minimum	25.8	19.8	14.0	0.0
maximum	105.3	80.0	95.8	82.8
range	79.5	60.2	81.8	82.8
mean	73.3	49.1	58.6	30.3
SD	20.6	18.5	20.0	19.9
SEM	3.3	5.6	3.2	3.0
lower 95% CI of mean	66.6	36.7	52.2	24.3
upper 95% CI of mean	79.9	61.5	65.1	36.3

Supplementary Table 22 | **Descriptive statistics of studies subdivided by the duration of the used tumor model (>29 days) (Fig. S9).**

Group	Therapy	Combination therapy	Nanotherapy	Combination nanotherapy
# data points	21	15	23	34
minimum	19.0	19.0	23.4	0.0
maximum	100.0	86.1	105.0	62.5
range	81.0	67.1	81.6	62.5
mean	65.0	56.4	58.0	23.3
SD	21.0	19.1	23.5	15.0
SEM	4.6	4.9	4.9	2.6
lower 95% CI of mean	55.4	45.8	47.8	18.1
upper 95% CI of mean	74.5	67.0	68.1	28.5

Supplementary Table 23 | **Descriptive statistics of co-delivery vs. separate delivery (Fig. 3a,b).**

Group	2 drugs in 2NP	2 drugs in 1NP	2 drugs in 2NP (internal)	2 drugs in 1NP (internal)
# data points	43	368	18	18
minimum	0.4	0.0	25.0	0.0
maximum	70.0	87.4	70.0	51.8
range	69.6	87.4	45.0	51.8
mean	32.1	23.1	46.6	27.2
SD	18.5	17.2	13.5	14.1
SEM	2.8	0.9	3.2	3.3
lower 95% CI of mean	26.4	21.3	39.8	20.3
upper 95% CI of mean	37.8	24.8	53.3	34.2

Supplementary Table 24 | **Descriptive statistics of (non-)PEGylated LP for combination nanotherapy (Fig. 3c).**

Group	PEGylated LP	non-PEGylated LP
# data points	91	20
minimum	0.0	11.5
maximum	76.9	82.8
range	76.9	71.3
mean	27.2	32.0
SD	19.5	18.0
SEM	2.0	4.0
lower 95% CI of mean	23.1	23.6
upper 95% CI of mean	31.2	40.4

Supplementary Table 25 | **Descriptive statistics of passive and active targeting strategies (Fig. 3d).**

Group	Therapy (single)	Therapy (combination)	Passive targeting (single)	Passive targeting (combination)	Active targeting (single)	Active targeting (combination)
# data points	313	111	301	344	74	94
minimum	6.8	0.0	5.8	0.0	11.1	0.0
maximum	110.3	86.4	121.2	87.4	97.4	62.5
range	103.5	86.4	115.4	87.4	86.3	62.5
mean	66.9	53.4	55.5	26.3	49.5	17.1
SD	20.7	17.9	21.9	18.5	19.6	13.2
SEM	1.2	1.7	1.3	1.0	2.3	1.4
lower 95% CI of mean	64.6	50.1	53.0	24.3	45.0	14.4
upper 95% CI of mean	69.2	56.80	57.9	28.3	54.1	19.8

Supplementary Table 26 | **Descriptive statistics of nanocarrier type for single-drug nanotherapy (Fig. 3e).**

Group	Therapy	Lipid nanotherapy	Polymeric nanotherapy	Inorganic nanotherapy	Lipid-polymer nanotherapy	Other nanotherapy
# data points	313	95	138	52	27	63
minimum	6.8	5.8	7.1	13.3	19.2	9.5
maximum	110.3	100.0	121.2	100.0	84.4	103.8
range	103.5	94.2	114.1	86.7	65.2	94.3
mean	66.9	57.1	54.4	49.7	54.6	53.3
SD	20.7	22.5	21.2	19.9	17.9	23.4
SEM	1.2	2.3	1.8	2.8	3.5	3.0
lower 95% CI of mean	64.6	52.5	50.9	44.1	47.5	47.6
upper 95% CI of mean	69.2	61.7	58.0	55.2	61.7	59.2

Supplementary Table 27 | **Descriptive statistics of nanocarrier type in combination nanotherapy (Fig. 3e).**

Group	Combination therapy	Lipid combination nanotherapy	Polymeric combination nanotherapy	Inorganic combination nanotherapy	Lipid-polymer combination nanotherapy	Other combination nanotherapy
# data points	111	111	154	60	28	85
minimum	0.0	0.0	0.0	0.0	0.0	0.0
maximum	86.4	82.8	87.4	75.8	75.4	77.8
range	86.4	82.8	87.4	75.8	75.4	77.8
mean	53.4	28.0	24.7	19.1	23.0	23.1
SD	17.9	19.2	17.4	15.9	17.3	17.9
SEM	1.7	1.8	1.4	2.1	3.3	1.9
lower 95% CI of mean	50.1	24.4	21.9	14.9	16.3	19.3
upper 95% CI of mean	56.8	31.6	27.4	23.2	29.7	27.0

Supplementary Table 28 | **Descriptive statistics of multidrug nanotherapies containing anthracyclines (primary drug) as major drug, in combination with other therapeutic compounds (secondary drug) (Fig. S12).**

Group	Therapy Primary drug	Therapy Secondary drug	Combination therapy	Nanotherapy Primary drug	Nanotherapy Secondary drug	Combination nanotherapy
# data points	83	48	38	104	73	187
minimum	19.0	6.8	3.9	9.5	5.8	0.0
maximum	97.1	102.7	77.8	105.0	100.0	87.4
range	78.1	95.9	73.9	95.5	94.2	87.4
mean	62.0	68.8	52.6	52.4	54.5	23.7
SD	19.8	22.3	18.4	20.3	21.9	18.5
SEM	2.2	3.2	3.0	2.0	2.6	1.4
lower 95% CI of mean	57.6	62.3	46.5	48.4	49.4	21.0
upper 95% CI of mean	66.3	75.3	58.6	56.3	59.6	26.3

Supplementary Table 29 | **Descriptive statistics of multidrug nanotherapies containing taxanes (primary drug) as major drug, in combination with other therapeutic compounds (secondary) (Fig. S12).**

Group	Therapy Primary drug	Therapy Secondary drug	Combination therapy	Nanotherapy Primary drug	Nanotherapy Secondary drug	Combination nanotherapy
# data points	44	40	32	47	39	113
minimum	9.6	19.0	3.9	6.4	18.4	0.0
maximum	100.0	105.3	86.4	97.4	105.0	76.9
range	90.4	86.3	82.5	91.0	86.6	76.9
mean	64.8	67.7	51.5	53.7	56.2	26.1
SD	20.9	20.7	18.2	23.0	22.7	19.0
SEM	3.2	3.3	3.2	3.4	3.6	1.8
lower 95% CI of mean	58.5	61.0	45.0	47.0	48.9	22.5
upper 95% CI of mean	71.2	74.3	58.1	60.5	63.6	29.6

Supplementary Table 30 | **Descriptive statistics of multidrug nanotherapies containing platinum-based drugs (primary drug) as major drug, in combination with other therapeutic compounds (secondary drug) (Fig. S12).**

Group	Therapy Primary drug	Therapy Secondary drug	Combination therapy	Nanotherapy Primary drug	Nanotherapy Secondary drug	Combination nanotherapy
# data points	18	17	20	25	16	51
minimum	17.2	33.3	28.3	5.8	27.8	0.0
maximum	100.0	102.1	86.1	86.9	91.0	75.4
range	82.8	68.8	57.8	81.1	63.2	75.4
mean	70.8	72.2	61.0	53.1	59.1	23.6
SD	20.7	20.4	16.6	20.7	20.5	17.7
SEM	4.9	4.9	3.7	4.1	5.1	2.5
lower 95% CI of mean	60.6	61.8	53.3	44.6	48.2	18.6
upper 95% CI of mean	81.1	82.7	68.8	61.6	70.1	28.6

Supplementary Table 31 | **Descriptive statistics of multidrug nanotherapies containing camptothecin-based drugs (primary drug) as major drug, in combination with other therapeutic compounds (secondary drug) (Fig. S12).**

Group	Therapy Primary drug	Therapy Secondary drug	Combination therapy	Nanotherapy Primary drug	Nanotherapy Secondary drug	Combination nanotherapy
# data points	21	17	13	12	12	42
minimum	34.7	27.2	29.1	11.5	16.5	0.0
maximum	82.2	94.3	86.1	78.6	103.8	87.4
range	47.5	67.1	57.0	67.1	87.3	87.4
mean	59.5	61.9	55.8	41.6	51.3	22.5
SD	14.0	20.6	18.3	21.6	26.3	20.2
SEM	3.1	5.0	5.1	6.2	7.6	3.1
lower 95% CI of mean	53.1	51.3	44.7	27.8	34.6	16.2
upper 95% CI of mean	65.8	72.5	66.8	55.3	68.0	28.8

Supplementary Table 32 | **Descriptive statistics of multidrug nanotherapies containing antimetabolites (primary drug) as major drug, in combination with other therapeutic compounds (secondary) (Fig. S12).**

Group	Therapy Primary drug	Therapy Secondary drug	Combination therapy	Nanotherapy Primary drug	Nanotherapy Secondary drug	Combination nanotherapy
# data points	12	12	16	18	19	52
minimum	12.2	18.2	19.8	22.2	23.4	0.0
maximum	90.0	93.2	76.1	93.2	100.0	60.0
range	77.8	75.0	56.3	71.0	76.6	60.0
mean	63.2	66.7	52.4	52.9	58.8	26.6
SD	19.9	19.5	13.9	19.8	22.1	15.0
SEM	5.8	5.6	3.5	4.7	5.1	2.1
lower 95% CI of mean	50.5	54.3	45.0	43.1	48.1	22.4
upper 95% CI of mean	75.9	79.1	59.7	62.7	69.4	30.8

Supplementary Table 33 | **Descriptive statistics of multidrug nanotherapies containing tyrosine-kinase inhibitors (primary drug) as major drug, in combination with other therapeutic compounds (secondary drug) (Fig. S12).**

Group	Therapy Primary drug	Therapy Secondary drug	Combination therapy	Nanotherapy Primary drug	Nanotherapy Secondary drug	Combination nanotherapy
# data points	13	11	10	8	14	37
minimum	47.6	20.0	34.6	22.6	14.6	1.1
maximum	100.0	88.3	75.8	98.5	121.2	76.9
range	52.4	68.3	41.2	75.9	106.6	75.8
mean	77.3	62.7	55.8	48.7	52.3	25.5
SD	13.9	22.6	11.2	23.1	28.9	17.2
SEM	3.9	6.8	3.5	8.2	7.7	2.8
lower 95% CI of mean	68.8	47.6	47.8	29.4	35.6	19.8
upper 95% CI of mean	85.7	77.9	63.8	68.1	69.0	31.2

Supplementary Table 34 | **Descriptive statistics of free-drug therapy efficacy in different cancers (Fig. 4a).**

Group	Brain	Breast	Cervix	Colon	Liver	Lung	Pancreatic	Prostate	Skin	Other
# data points	12	147	17	25	24	29	12	7	15	25
minimum	40.7	19.0	9.6	39.0	27.5	23.3	51.9	60.0	10.2	6.8
maximum	82.0	109.4	100.0	100.0	88.6	100.0	101.9	96.1	110.3	92.5
range	41.3	90.4	90.4	61.0	61.1	76.7	50.0	36.1	100.1	85.7
mean	62.1	67.1	59.3	69.8	67.5	74.5	69.1	81.2	62.3	58.9
SD	14.9	20.8	27.0	18.6	14.6	19.3	14.8	13.5	22.4	25.3
SEM	4.3	1.7	6.6	3.7	3.0	3.6	4.3	5.1	5.8	5.1
lower 95% CI of mean	52.7	63.7	45.4	62.1	61.3	67.2	59.7	68.8	49.9	48.4
upper 95% CI of mean	71.6	70.5	73.2	77.5	73.6	81.9	78.4	93.7	74.7	69.3

Supplementary Table 35 | **Descriptive statistics of free-drug comb. therapy efficacy in different cancers (Fig. 4b).**

Group	Brain	Breast	Cervix	Colon	Liver	Lung	Pancreatic	Prostate	Skin	Other
# data points	7	37	4	14	8	16	7	2	7	9
minimum	36.7	9.1	52.1	29.7	28.3	27.5	26.3	50.0	0.0	3.9
maximum	84.4	86.4	66.0	86.1	65.9	81.4	71.0	69.5	80.0	76.3
range	47.7	77.3	13.9	56.4	37.6	53.9	44.7	19.5	80.0	72.4
mean	58.4	49.5	56.3	59.8	55.4	58.6	49.0	59.8	49.5	48.7
SD	17.7	18.4	6.5	16.6	12.12	15.5	14.7	13.8	29.1	22.5
SEM	6.7	3.0	3.3	4.4	4.3	3.9	5.6	9.8	11.0	7.5
lower 95% CI of mean	42.1	43.4	46.0	50.3	45.3	50.3	35.3	-64.14	22.6	31.4
upper 95% CI of mean	74.8	55.6	66.7	69.4	65.5	66.8	62.6	183.6	76.4	66.0

Supplementary Table 36 | **Descriptive statistics of singe-drug nanotherapy efficacy in different cancers (Fig. 4c).**

Group	Brain	Breast	Cervix	Colon	Liver	Lung	Pancreatic	Prostate	Skin	Other
# data points	12	157	23	37	33	33	24	9	20	27
minimum	24.0	5.8	6.4	12.4	18.4	17.5	23.4	42.1	7.1	22.0
maximum	103.8	105.0	93.2	100.0	84.4	121.2	100.0	97.4	100.0	100.0
range	79.8	99.2	86.8	87.6	66.0	103.7	76.6	55.3	92.9	78.0
mean	52.4	54.7	48.1	56.4	48.3	58.6	56.2	64.5	51.7	53.9
SD	21.0	21.5	21.25	21.1	17.1	25.1	20.0	16.7	27.5	20.8
SEM	6.1	1.7	4.4	3.5	3.0	4.4	4.1	5.6	6.1	4.0
lower 95% CI of mean	39.0	51.3	38.9	49.4	42.2	49.7	47.8	51.7	38.8	45.7
upper 95% CI of mean	65.7	58.1	57.3	63.4	54.3	67.5	64.7	77.3	64.5	62.1

Supplementary Table 37 | **Descriptive statistics of combination nanotherapy efficacy in different cancers (Fig. 4d).**

Group	Brain	Breast	Cervix	Colon	Liver	Lung	Pancreatic	Prostate	Skin	Other
# data points	22	192	15	36	31	50	28	11	21	32
minimum	1.5	0	0.0	0.0	0.0	0.0	2.6	0.0	0.0	0.0
maximum	75.4	87.4	40.9	51.1	52.4	70.0	72.2	67.8	54.2	82.8
range	73.9	87.4	40.9	51.1	52.4	70.0	69.6	67.8	54.2	82.8
mean	27.6	25.6	20.8	19.0	18.2	28.4	29.4	23.3	17.3	22.4
SD	22.3	18.5	15.7	13.1	14.2	17.6	15.0	20.0	17.7	19.4
SEM	4.8	1.3	4.1	2.2	2.6	2.5	2.8	6.0	3.9	3.4
lower 95% CI of mean	17.7	22.9	12.1	14.5	13.0	23.4	23.6	9.9	9.3	15.4
upper 95% CI of mean	37.5	28.2	29.5	23.4	23.4	33.4	35.2	36.7	25.4	29.4

Supplementary Table 38 | **Descriptive statistics of therapy efficacy in sensitive cancers (Fig. 5a).**

Group	Therapy	Combination therapy	Nanotherapy	Combination nanotherapy
# data points	271	97	322	382
minimum	9.6	0.0	5.8	0.0
maximum	110.3	86.4	105.0	87.4
range	100.7	86.4	99.2	87.4
mean	66.8	53.6	53.5	24.5
SD	20.7	18.3	21.1	18.0
SEM	1.3	1.9	1.2	0.9
lower 95% CI of mean	64.4	50.0	51.2	22.7
upper 95% CI of mean	69.3	57.3	55.9	26.3

Supplementary Table 39 | **Descriptive statistics of therapy efficacy in resistant cancers (Fig. 5a).**

Group	Therapy	Combination therapy	Nanotherapy	Combination nanotherapy
# data points	42	14	53	56
minimum	6.8	28.3	17.8	0.0
maximum	100.0	76.3	121.2	75.0
range	93.2	48.0	103.4	75.0
mean	67.4	52.2	58.8	23.5
SD	21.0	15.4	24.0	17.2
SEM	3.2	4.1	3.3	2.3
lower 95% CI of mean	60.8	43.3	52.1	18.9
upper 95% CI of mean	73.9	61.2	65.4	28.1

Supplementary Table 40 | **Descriptive statistics of therapy efficacy in xenograft tumor models (Fig. 5b).**

Group	Therapy	Combination therapy	Nanotherapy	Combination nanotherapy
# data points	191	68	230	261
minimum	6.8	3.9	6.4	0.0
maximum	109.4	86.4	121.2	82.8
range	102.6	82.5	114.8	82.8
mean	66.8	53.3	55.4	23.5
SD	21.0	17.1	21.4	17.3
SEM	1.5	2.1	1.4	1.1
lower 95% CI of mean	63.8	49.1	52.6	21.4
upper 95% CI of mean	69.7	57.4	58.2	25.6

Supplementary Table 41 | **Descriptive statistics of therapy efficacy in allograft tumor models (Fig. 5b).**

Group	Therapy	Combination therapy	Nanotherapy	Combination nanotherapy
# data points	122	43	145	177
minimum	10.2	0.0	5.8	0.0
maximum	110.3	80.0	100.0	87.4
range	100.1	80.0	94.2	87.4
mean	67.1	53.7	52.5	25.5
SD	20.4	19.4	21.8	18.8
SEM	1.8	3.0	1.8	1.4
lower 95% CI of mean	63.5	47.8	48.9	22.7
upper 95% CI of mean	70.8	59.7	56.1	28.3

Supplementary Table 42 | **P-values for comparative statistical analysis displayed in Fig. 2a.**

Single comparisons	P-value
Therapy vs. Combination therapy	<0.0001
Therapy vs. Nanotherapy	<0.0001
Therapy vs. Combination nanotherapy	<0.0001
Combination therapy vs. Nanotherapy	>0.9999
Combination therapy vs. Combination nanotherapy	<0.0001

Supplementary Table 43 | **P-values for comparative statistical analysis displayed in Fig. 3.**

Single comparisons	P-value
**3a,b**	
2 drugs in 2 NP vs. 2 drugs 1 NP	0.0016
2 drugs in 2 NP vs. 2 drugs 1 NP internal	0.0002
**3c**	
non-PEGylated LP vs. PEGylated LP	0.2808
**3d Passive vs. active targeting**	
Therapy (S) vs. Therapy (C)	0.0002
Therapy (S) vs. Passive targeting (S)	<0.0001
Therapy (S) vs. Passive targeting (C)	<0.0001
Therapy (S) vs. Active targeting (S)	<0.0001
Therapy (S) vs. Active targeting (C)	<0.0001
Therapy (C) vs. Passive targeting (S)	>0.9999
Therapy (C) vs. Passive targeting (C)	<0.0001
Therapy (C) vs. Active targeting (S)	>0.9999
Passive targeting (S) vs. Passive targeting (C)	<0.0001
Passive targeting (S) vs. Active targeting (S)	>0.9999
Passive targeting (S) vs. Active targeting (C)	<0.0001
Passive targeting (C) vs. Active targeting (S)	<0.0001
Passive targeting (C) vs. Active targeting (C)	0.0401
Active targeting (S) vs. Active targeting (C)	<0.0001

Supplementary Table 44 | **P-values for comparative statistical analysis displayed in Fig. 3e.**

Single comparisons	P-value
Therapy (S) vs. Therapy (C)	0.0009
Therapy (S) vs. Lipid (S)	0.0801
Therapy (S) vs. Polymeric (S)	0.0003
Therapy (S) vs. Inorganic (S)	0.0006
Therapy (S) vs. Lipid-polymer (S)	>0.9999
Therapy (S) vs. Other (S)	0.0124
Therapy (S) vs. Lipid (C)	<0.0001
Therapy (S) vs. Polymeric (C)	<0.0001
Therapy (S) vs. Lipid-polymer (C)	<0.0001
Therapy (S) vs. Inorganic (C)	<0.0001
Therapy (S) vs. Other (C)	<0.0001
Therapy (C) vs. Lipid (S)	>0.9999
Therapy (C) vs. Polymeric (S)	>0.9999
Therapy (C) vs. Lipid-polymer (S)	>0.9999
Therapy (C) vs. Inorganic (S)	>0.9999
Therapy (C) vs. Other (S)	>0.9999
Therapy (C) vs. Lipid (C)	<0.0001
Therapy (C) vs. Polymeric (C)	<0.0001
Therapy (C) vs. Lipid-polymer (C)	<0.0001
Therapy (C) vs. Inorganic (C)	<0.0001
Therapy (C) vs. Other (C)	<0.0001
Lipid (S) vs. Polymeric (S)	>0.9999
Lipid (S) vs. Lipid-polymer (S)	>0.9999
Lipid (S) vs. Inorganic (S)	>0.9999
Lipid (S) vs. Other (S)	>0.9999
Lipid (S) vs. Lipid (C)	<0.0001
Lipid (S) vs. Polymeric (C)	<0.0001
Lipid (S) vs. Lipid-polymer (C)	<0.0001
Lipid (S) vs. Inorganic (C)	<0.0001
Lipid (S) vs. Other (C)	<0.0001
Polymeric (S) vs. Lipid-polymer (S)	>0.9999
Polymeric (S) vs. Inorganic (S)	>0.9999
Polymeric (S) vs. Other (S)	>0.9999
Polymeric (S) vs. Lipid (C)	<0.0001
Polymeric (S) vs. Polymeric (C)	<0.0001
Polymeric (S) vs. Lipid-polymer (C)	<0.0001
Polymeric (S) vs. Inorganic (C)	<0.0001
Polymeric (S) vs. Other (C)	<0.0001
Lipid-polymer (S) vs. Inorganic (S)	>0.9999
Lipid-polymer (S) vs. Other (S)	>0.9999
Lipid-polymer (S) vs. Lipid (C)	<0.0001
Lipid-polymer (S) vs. Polymeric (C)	<0.0001
Lipid-polymer (S) vs. Lipid-polymer (C)	0.0003
Lipid-polymer (S) vs. Inorganic (C)	<0.0001
Lipid-polymer (S) vs. Other (C)	<0.0001
Inorganic (S) vs. Other (S)	>0.9999

Supplementary Table 45 | **P-values for comparative statistical analysis displayed in Fig. 5a.**

**Single comparisons**	**P-value**
Therapy (S) vs. Combination therapy (S)	0.0022
Therapy (S) vs. Nanotherapy (S)	<0.0001
Therapy (S) vs. Combination nanotherapy (S)	<0.0001
Therapy (S) vs. Therapy (R)	>0.9999
Therapy (S) vs. Combination therapy (R)	>0.9999
Therapy (S) vs. Nanotherapy (R)	0.6910
Therapy (S) vs. Combination nanotherapy (R)	<0.0001
Combination therapy (S) vs. Nanotherapy (S)	>0.9999
Combination therapy (S) vs. Combination nanotherapy (S)	<0.0001
Combination therapy (S) vs. Therapy (R)	0.2101
Combination therapy (S) vs. Combination therapy (R)	>0.9999
Combination therapy (S) vs. Nanotherapy (R)	>0.9999
Combination therapy (S) vs. Combination nanotherapy (R)	<0.0001
Nanotherapy (S) vs. Combination nanotherapy (S)	<0.0001
Nanotherapy (S) vs. Therapy (R)	0.0379
Nanotherapy (S) vs. Combination therapy (R)	>0.9999
Nanotherapy (S) vs. Nanotherapy (R)	>0.9999
Nanotherapy (S) vs. Combination nanotherapy (R)	<0.0001
Combination nanotherapy (S) vs. Therapy (R)	<0.0001
Combination nanotherapy (S) vs. Combination therapy (R)	0.0018
Combination nanotherapy (S) vs. Nanotherapy (R)	<0.0001
Combination nanotherapy (S) vs. Combination nanotherapy (R)	>0.9999
Therapy (R) vs. Combination therapy (R)	>0.9999
Therapy (R) vs. Nanotherapy (R)	>0.9999
Therapy (R) vs. Combination nanotherapy (R)	<0.0001
Combination therapy (R) vs. Nanotherapy (R)	>0.9999
Combination therapy (R) vs. Combination nanotherapy (R)	0.0044

Supplementary Table 46 | **P-values for comparative statistical analysis displayed in Fig. 5b.**

**Single comparisons**	**P-value**
Therapy (X) vs. Combination therapy (X)	0.0160
Therapy (X) vs. Nanotherapy (X)	0.0002
Therapy (X) vs. Combination nanotherapy (X)	<0.0001
Therapy (X) vs. Therapy (A)	>0.9999
Therapy (X) vs. Combination therapy (A)	01997
Therapy (X) vs. Nanotherapy (A)	<0.0001
Therapy (X) vs. Combination nanotherapy (A)	<0.0001
Combination therapy (X) vs. Nanotherapy (X)	>0.9999
Combination therapy (X) vs. Combination nanotherapy (X)	<0.0001
Combination therapy (X) vs. Therapy (A)	0.0266
Combination therapy (X) vs. Combination therapy (A)	>0.9999
Combination therapy (X) vs. Nanotherapy (A)	>0.9999
Combination therapy (X) vs. Combination nanotherapy (A)	<0.0001
Nanotherapy (X) vs. Combination nanotherapy (X)	<0.0001
Nanotherapy (X) vs. Therapy (A)	0.0015
Nanotherapy (X) vs. Combination therapy (A)	>0.9999
Nanotherapy (X) vs. Nanotherapy (A)	>0.9999
Nanotherapy (X) vs. Combination nanotherapy (A)	<0.0001
Combination nanotherapy (X) vs. Therapy (A)	<0.0001
Combination nanotherapy (X) vs. Combination therapy (A)	<0.0001
Combination nanotherapy (X) vs. Nanotherapy (A)	<0.0001
Combination nanotherapy (X) vs. Combination nanotherapy (A)	>0.9999
Therapy (A) vs. Combination therapy (A)	0.2341
Therapy (A) vs. Nanotherapy (A)	0,0002
Therapy (A) vs. Combination nanotherapy (A)	<0.0001
Combination therapy (A) vs. Nanotherapy (A)	>0.9999
Combination therapy (A) vs. Combination nanotherapy (A)	<0.0001

Supplementary Table 47 | **P-values for comparative statistical analysis displayed in Fig. S3.**

**Single comparisons**	**P-value**
**S3a Combination therapy with proven synergy**	
Therapy vs. Combination therapy	0.0376
Therapy vs. Nanotherapy	<0.0001
Therapy vs. Combination nanotherapy	<0.0001
Combination therapy vs. Nanotherapy	>0.9999
Combination therapy vs. Combination nanotherapy	<0.0001
Nanotherapy vs. Combination nanotherapy	<0.0001
**S3b Combination therapy with unknown synergy**	
Therapy vs. Combination therapy	0.0041
Therapy vs. Nanotherapy	<0.0001
Therapy vs. Combination nanotherapy	<0.0001
Combination therapy vs. Nanotherapy	>0.9999
Combination therapy vs. Combination nanotherapy	<0.0001

Supplementary Table 48 | **P-values for comparative statistical analysis displayed in Fig. S4.**

**Single comparisons**	**P-value**
**S4a Two-drug combination therapies**	<0.0001
Therapy (1 drug) vs. Combination therapy (2 drugs)	0.0001
Therapy (1 drug) vs. Nanotherapy (1 drug)	<0.0001
Therapy (1 drug) vs. Combination nanotherapy (2 drugs)	<0.0001
Combination therapy (2 drugs) vs. Nanotherapy (1 drug)	>0.9999
Combination therapy (2 drugs) vs. Combination nanotherapy (2 drugs)	<0.0001
Nanotherapy (1 drug) vs. Combination nanotherapy (2 drugs)	<0.0001
**S4b Three-drug combination therapies**	<0.0001
Therapy (1 drug) vs. Combination therapy (3 drugs)	>0.9999
Therapy (1 drug) vs. Nanotherapy (1 drug)	>0.9999
Therapy (1 drug) vs. Combination nanotherapy (3 drugs)	<0.0001
Combination therapy (3 drugs) vs. Nanotherapy (1 drug)	>0.9999
Combination therapy (3 drugs) vs. Combination nanotherapy (3 drugs)	0.0016
Nanotherapy (1 drug) vs. Combination nanotherapy (3 drugs)	<0.0001

Supplementary Table 49 | **P-values for comparative statistical analysis displayed in Fig. S6.**

**Single comparisons**	**P-value**
**y ≤ 2**	
Therapy vs. Combination therapy	0.4061
Therapy vs. Nanotherapy	0.3310
Therapy vs. Combination nanotherapy	<0.0001
Combination therapy vs. Nanotherapy	>0.9999
Combination therapy vs. Combination nanotherapy	0.0183
Therapy vs. Combination therapy	<0.0001
**y ≤ 3 - 7**	
Therapy vs. Combination therapy	>0.9999
Therapy vs. Nanotherapy	0.6192
Therapy vs. Combination nanotherapy	<0.0001
Combination therapy vs. Nanotherapy	>0.9999
Combination therapy vs. Combination nanotherapy	<0.0001
Therapy vs. Combination therapy	<0.0001
**y ≤ 8 - 14**	
Therapy vs. Combination therapy	0.0059
Therapy vs. Nanotherapy	<0.0001
Therapy vs. Combination nanotherapy	<0.0001
Combination therapy vs. Nanotherapy	>0.9999
Combination therapy vs. Combination nanotherapy	<0.0001
Therapy vs. Combination therapy	<0.0001
**y > 14**	
Therapy vs. Combination therapy	0.0278
Therapy vs. Nanotherapy	0.0022
Therapy vs. Combination nanotherapy	<0.0001
Combination therapy vs. Nanotherapy	>0.9999
Combination therapy vs. Combination nanotherapy	<0.0001
Therapy vs. Combination therapy	<0.0001

Supplementary Table 50 | **P-values for comparative statistical analysis displayed in Fig. S7.**

**Single comparisons**	**P-value**
**z = 1**	
Therapy vs. Combination therapy	>0.9999
Therapy vs. Nanotherapy	0.4736
Therapy vs. Combination nanotherapy	<0.0001
Combination therapy vs. Nanotherapy	>0.9999
Combination therapy vs. Combination nanotherapy	0.0080
Therapy vs. Combination therapy	<0.0001
**z = 2 - 4**	
Therapy vs. Combination therapy	0.1807
Therapy vs. Nanotherapy	0.0003
Therapy vs. Combination nanotherapy	<0.0001
Combination therapy vs. Nanotherapy	>0.9999
Combination therapy vs. Combination nanotherapy	<0.0001
Therapy vs. Combination therapy	<0.0001
**z = 5 - 7**	
Therapy vs. Combination therapy	0.0023
Therapy vs. Nanotherapy	0.0020
Therapy vs. Combination nanotherapy	<0.0001
Combination therapy vs. Nanotherapy	>0.9999
Combination therapy vs. Combination nanotherapy	<0.0001
Therapy vs. Combination therapy	<0.0001
**z > 7**	
Therapy vs. Combination therapy	0.4612
Therapy vs. Nanotherapy	0.1433
Therapy vs. Combination nanotherapy	<0.0001
Combination therapy vs. Nanotherapy	>0.9999
Combination therapy vs. Combination nanotherapy	<0.0001
Therapy vs. Combination therapy	<0.0001

Supplementary Table 51 | **P-values for comparative statistical analysis displayed in Fig. S8.**

Single comparisons	P-value
**a = 0 - 3**	
Therapy vs. Combination therapy	0.0177
Therapy vs. Nanotherapy	0.0004
Therapy vs. Combination nanotherapy	<0.0001
Combination therapy vs. Nanotherapy	>0.9999
Combination therapy vs. Combination nanotherapy	<0.0001
Therapy vs. Combination therapy	<0.0001
**a = 4 - 9**	
Therapy vs. Combination therapy	0.5909
Therapy vs. Nanotherapy	0.0043
Therapy vs. Combination nanotherapy	<0.0001
Combination therapy vs. Nanotherapy	>0.9999
Combination therapy vs. Combination nanotherapy	<0.0001
Therapy vs. Combination therapy	<0.0001
**a > 9**	
Therapy vs. Combination therapy	0.1092
Therapy vs. Nanotherapy	0.0168
Therapy vs. Combination nanotherapy	<0.0001
Combination therapy vs. Nanotherapy	>0.9999
Combination therapy vs. Combination nanotherapy	<0.0001
Therapy vs. Combination therapy	<0.0001

Supplementary Table 52 | **P-values for comparative statistical analysis displayed in Fig. S9.**

Single comparisons	p-value
**x < 21**	
Therapy vs. Combination therapy	>0.9999
Therapy vs. Nanotherapy	0.6203
Therapy vs. Combination nanotherapy	<0.0001
Combination therapy vs. Nanotherapy	>0.9999
Combination therapy vs. Combination nanotherapy	0.1862
Therapy vs. Combination therapy	0.0002
**x = 21 - 29**	
Therapy vs. Combination therapy	0.0405
Therapy vs. Nanotherapy	0.1086
Therapy vs. Combination nanotherapy	<0.0001
Combination therapy vs. Nanotherapy	>0.9999
Combination therapy vs. Combination nanotherapy	0.2137
Therapy vs. Combination therapy	<0.0001
**x > 29**	
Therapy vs. Combination therapy	>0.9999
Therapy vs. Nanotherapy	>0.9999
Therapy vs. Combination nanotherapy	<0.0001
Combination therapy vs. Nanotherapy	>0.9999
Combination therapy vs. Combination nanotherapy	0.0001
Therapy vs. Combination therapy	<0.0001

Supplementary Table 53 | **P-values for comparative statistical analysis displayed in Fig. S12.**

Single comparisons	P-value
**Anthracyclines**	
Therapy (Primary drug) vs. Therapy (Secondary drug)	>0.9999
Therapy (Primary drug) vs. Combination therapy	>0.9999
Therapy (Primary drug) vs. Nanotherapy (Primary drug)	0.1618
Therapy (Primary drug) vs. Nanotherapy (Secondary drug)	>0.9999
Therapy (Primary drug) vs. Combination nanotherapy	<0.0001
Therapy (Secondary drug) vs. Combination therapy	0.1658
Therapy (Secondary drug) vs. Nanotherapy (Primary drug)	0.0073
Therapy (Secondary drug) vs. Nanotherapy (Secondary drug)	0.0803
Therapy (Secondary drug) vs. Combination nanotherapy	<0.0001
Combination therapy vs. Nanotherapy (Primary drug)	>0.9999
Combination therapy vs. Nanotherapy (Secondary drug)	>0.9999
Combination therapy vs. Combination nanotherapy	<0.0001
Nanotherapy (Primary drug) vs. Nanotherapy (Secondary drug)	>0.9999
Nanotherapy (Primary drug) vs. Combination nanotherapy	<0.0001
Nanotherapy (Secondary drug) vs. Combination nanotherapy	<0.0001
**Taxanes**	
Therapy (Primary drug) vs. Therapy (Secondary drug)	>0.9999
Therapy (Primary drug) vs. Combination therapy	0.4751
Therapy (Primary drug) vs. Nanotherapy (Primary drug)	0.5700
Therapy (Primary drug) vs. Nanotherapy (Secondary drug)	>0.9999
Therapy (Primary drug) vs. Combination nanotherapy	<0.0001
Therapy (Secondary drug) vs. Combination therapy	0.1618
Therapy (Secondary drug) vs. Nanotherapy (Primary drug)	0.1792
Therapy (Secondary drug) vs. Nanotherapy (Secondary drug)	0.6441
Therapy (Secondary drug) vs. Combination nanotherapy	<0.0001
Combination therapy vs. Nanotherapy (Primary drug)	>0.9999
Combination therapy vs. Nanotherapy (Secondary drug)	>0.9999
Combination therapy vs. Combination nanotherapy	<0.0001
Nanotherapy (Primary drug) vs. Nanotherapy (Secondary drug)	>0.9999
Nanotherapy (Primary drug) vs. Combination nanotherapy	<0.0001
Nanotherapy (Secondary drug) vs. Combination nanotherapy	<0.0001

Supplementary Table 54 | **P-values for comparative statistical analysis displayed in Fig. S12.**

**Single comparisons**	**P-value**
**Platinum-based drugs**	
Therapy (Primary drug) vs. Therapy (Secondary drug)	>0.9999
Therapy (Primary drug) vs. Combination therapy	>0.9999
Therapy (Primary drug) vs. Nanotherapy (Primary drug)	0.4704
Therapy (Primary drug) vs. Nanotherapy (Secondary drug)	>0.9999
Therapy (Primary drug) vs. Combination nanotherapy	<0.0001
Therapy (Secondary drug) vs. Combination therapy	>0.9999
Therapy (Secondary drug) vs. Nanotherapy (Primary drug)	0.3107
Therapy (Secondary drug) vs. Nanotherapy (Secondary drug)	>0.9999
Therapy (Secondary drug) vs. Combination nanotherapy	<0.0001
Combination therapy vs. Nanotherapy (Primary drug)	>0.9999
Combination therapy vs. Nanotherapy (Secondary drug)	>0.9999
Combination therapy vs. Combination nanotherapy	<0.0001
Nanotherapy (Primary drug) vs. Nanotherapy (Secondary drug)	>0.9999
Nanotherapy (Primary drug) vs. Combination nanotherapy	0.0002
Nanotherapy (Secondary drug) vs. Combination nanotherapy	<0.0001
**Camptothecin (and derivatives)**	
Therapy (Primary drug) vs. Therapy (Secondary drug)	>0.9999
Therapy (Primary drug) vs. Combination therapy	>0.9999
Therapy (Primary drug) vs. Nanotherapy (Primary drug)	0.5682
Therapy (Primary drug) vs. Nanotherapy (Secondary drug)	>0.9999
Therapy (Primary drug) vs. Combination nanotherapy	<0.0001
Therapy (Secondary drug) vs. Combination therapy	>0.9999
Therapy (Secondary drug) vs. Nanotherapy (Primary drug)	0.4859
Therapy (Secondary drug) vs. Nanotherapy (Secondary drug)	>0.9999
Therapy (Secondary drug) vs. Combination nanotherapy	<0.0001
Combination therapy vs. Nanotherapy (Primary drug)	>0.9999
Combination therapy vs. Nanotherapy (Secondary drug)	>0.9999
Combination therapy vs. Combination nanotherapy	0.0004
Nanotherapy (Primary drug) vs. Nanotherapy (Secondary drug)	>0.9999
Nanotherapy (Primary drug) vs. Combination nanotherapy	0.3203
Nanotherapy (Secondary drug) vs. Combination nanotherapy	0.0175

Supplementary Table 55 | **P-values for comparative statistical analysis displayed in Fig. S12.**

Single comparisons	P-value
**Antimetabolites**	
Therapy (Primary drug) vs. Therapy (Secondary drug)	>0.9999
Therapy (Primary drug) vs. Combination therapy	>0.9999
Therapy (Primary drug) vs. Nanotherapy (Primary drug)	>0.9999
Therapy (Primary drug) vs. Nanotherapy (Secondary drug)	>0.9999
Therapy (Primary drug) vs. Combination nanotherapy	<0.0001
Therapy (Secondary drug) vs. Combination therapy	>0.9999
Therapy (Secondary drug) vs. Nanotherapy (Primary drug)	>0.9999
Therapy (Secondary drug) vs. Nanotherapy (Secondary drug)	>0.9999
Therapy (Secondary drug) vs. Combination nanotherapy	<0.0001
Combination therapy vs. Nanotherapy (Primary drug)	>0.9999
Combination therapy vs. Nanotherapy (Secondary drug)	>0.9999
Combination therapy vs. Combination nanotherapy	0.0006
Nanotherapy (Primary drug) vs. Nanotherapy (Secondary drug)	>0.9999
Nanotherapy (Primary drug) vs. Combination nanotherapy	0.0004
Nanotherapy (Secondary drug) vs. Combination nanotherapy	<0.0001
**Tyrosine kinase inhibitors**	
Therapy (Primary drug) vs. Therapy (Secondary drug)	>0.9999
Therapy (Primary drug) vs. Combination therapy	>0.9999
Therapy (Primary drug) vs. Nanotherapy (Primary drug)	0.2687
Therapy (Primary drug) vs. Nanotherapy (Secondary drug)	0.1841
Therapy (Primary drug) vs. Combination nanotherapy	<0.0001
Therapy (Secondary drug) vs. Combination therapy	>0.9999
Therapy (Secondary drug) vs. Nanotherapy (Primary drug)	>0.9999
Therapy (Secondary drug) vs. Nanotherapy (Secondary drug)	>0.9999
Therapy (Secondary drug) vs. Combination nanotherapy	0.0005
Combination therapy vs. Nanotherapy (Primary drug)	>0.9999
Combination therapy vs. Nanotherapy (Secondary drug)	>0.9999
Combination therapy vs. Combination nanotherapy	0.0077
Nanotherapy (Primary drug) vs. Nanotherapy (Secondary drug)	>0.9999
Nanotherapy (Primary drug) vs. Combination nanotherapy	0.3627
Nanotherapy (Secondary drug) vs. Combination nanotherapy	0.0274

Manuscripts included in the analysis

1. Gaber, M. *et al.* Co-Administration of Tretinoin Enhances the Anti-Cancer Efficacy of Etoposide via Tumor-Targeted Green Nano-Micelles. *Colloids Surfaces B Biointerfaces* **192**, 110997 (2020).
2. Xiong, Q. *et al.* Cuprous oxide nanoparticles trigger reactive oxygen species-induced apoptosis through activation of erk-dependent autophagy in bladder cancer. *Cell Death Dis.* **11**, 366 (2020).
3. Bao, Y. *et al.* Synergistic Chemotherapy for Breast Cancer and Breast Cancer Brain Metastases via Paclitaxel-Loaded Oleanolic Acid Nanoparticles. *Mol. Pharm.* **17**, 1343–1351 (2020).
4. Sun, J. *et al.* Triple drugs co-delivered by a small gemcitabine-based carrier for pancreatic cancer immunochemotherapy. *Acta Biomater.* **106**, 289–300 (2020).
5. Luo, L. *et al.* Stimuli-responsive polymeric prodrug-based nanomedicine delivering nifuroxazide and doxorubicin against primary breast cancer and pulmonary metastasis. *J. Control. Release* **318**, 124–135 (2020).
6. Hong, Y. *et al.* Combination therapy of lung cancer using layer-by-layer cisplatin prodrug and curcumin co-encapsulated nanomedicine. *Drug Des. Devel. Ther.* **14**, 2263–2274 (2020).
7. Li, Y., Hou, H., Zhang, P. & Zhang, Z. Co-delivery of doxorubicin and paclitaxel by reduction/pH dual responsive nanocarriers for osteosarcoma therapy. *Drug Deliv.* **27**, 1044–1053 (2020).
8. Zhang, X. *et al.* Hyaluronic acid reduction-sensitive polymeric micelles achieving co-delivery of tumor-targeting paclitaxel/apatinib effectively reverse cancer multidrug resistance. *Drug Deliv.* **27**, 825–835 (2020).
9. Mei, D. *et al.* Actively priming autophagic cell death with novel transferrin receptor-targeted nanomedicine for synergistic chemotherapy against breast cancer. *Acta Pharm. Sin. B* **9**, 1061–1077 (2019).
10. Shan, L. *et al.* Self-assembled green tea polyphenol-based coordination nanomaterials to improve chemotherapy efficacy by inhibition of carbonyl reductase 1. *Biomaterials* **210**, 62–69 (2019).
11. Chen, Y. *et al.* Small Molecular Theranostic Assemblies Functionalized by Doxorubicin-Hyaluronic Acid-Methotrexate Prodrug for Multiple Tumor Targeting and Imaging-Guided Combined Chemo-Photothermal Therapy. *Mol. Pharm.* **16**, 2470–2480 (2019).
12. Zhang, H., Zhang, Q., Liu, C. & Han, B. Preparation of a one-dimensional nanorod/metal organic framework Janus nanoplatform: Via side-specific growth for synergistic cancer therapy. *Biomater. Sci.* **7**, 1696–1704 (2019).
13. Lim, C. *et al.* A nano-complex system to overcome antagonistic photo-chemo combination cancer therapy. *J. Control. Release* **295**, 164–173 (2019).
14. Wang, J. *et al.* Polyphenol-based nanoplatform for MRI/PET dual-modality imaging guided effective combination chemotherapy. *J. Mater. Chem. B* **7**, 5688–5694 (2019).
15. Soni, K. S. *et al.* A polymeric nanogel-based treatment regimen for enhanced efficacy and sequential administration of synergistic drug combination in pancreatic cancer. *J. Pharmacol. Exp. Ther.* **370**, 894–901 (2019).
16. Liu, T., Deng, Y., Yao, J., Xiong, H. & Yao, J. Assembly simulation and synergistic chemotherapy of TPGS derivative functionalized polymersomes in hepatocellular carcinoma. *Nanomedicine* **14**, 1707–1727 (2019).
17. Lei, M. *et al.* Dual-functionalized liposome by co-delivery of paclitaxel with sorafenib for synergistic antitumor efficacy and reversion of multidrug resistance. *Drug Deliv.* **26**, 262–272 (2019).
18. Thirunavukkarasu, G. K. *et al.* Magnetic field-inducible drug-eluting nanoparticles for image-guided thermo-chemotherapy. *Biomaterials* **180**, 240–252 (2018).
19. Hou, M. *et al.* Methotrexate-based amphiphilic prodrug nanoaggregates for co-administration of multiple therapeutics and synergistic cancer therapy. *Acta Biomater.* **77**, 228–239 (2018).
20. Yang, H. *et al.* Charge-reversal-functionalized PLGA nanobubbles as theranostic agents for ultrasonic-imaging-guided combination therapy. *Biomaterials Science* **6**, 2426–2439 (2018).
21. González-Fernández, Y., Brown, H. K., Patiño-García, A., Heymann, D. & Blanco-Prieto, M. J. Oral administration of edelfosine encapsulated lipid nanoparticles causes regression of lung metastases in pre-clinical models of osteosarcoma. *Cancer Lett.* **430**, 193–200 (2018).
22. Panagi, M. *et al.* TGF-β inhibition combined with cytotoxic nanomedicine normalizes triple negative breast cancer microenvironment towards anti-tumor immunity. *Theranostics* **10**, 1910–1922 (2020).
23. Fernandes, R. S. *et al.* Nanostructured Lipid Carrier Co-loaded with Doxorubicin and Docosahexaenoic Acid as a Theranostic Agent: Evaluation of Biodistribution and Antitumor Activity in Experimental Model. *Mol. Imaging Biol.* **20**, 437–447 (2018).
24. Kang, X. *et al.* Liposomal Codelivery of Doxorubicin and Andrographolide Inhibits Breast Cancer Growth and Metastasis. *Mol. Pharm.* **15**, 1618–1626 (2018).
25. Thomas A. et al. Selective mTORC2 inhibitor therapeutically blocks breast cancer cell growth and survival. *Cancer Res.* **176**, 139–148 (2018).
26. Mao, C., Li, F., Zhao, Y., Debinski, W. & Ming, X. P-glycoprotein-targeted photodynamic therapy boosts cancer nanomedicine by priming tumor microenvironment. *Theranostics* **8**, 6274–6290 (2018).
27. Yang, F., Li, A., Liu, H. & Zhang, H. Gastric cancer combination therapy: Synthesis of a hyaluronic acid and cisplatin containing lipid prodrug coloaded with sorafenib in a nanoparticulate system to exhibit enhanced anticancer efficacy and reduced toxicity. *Drug Des. Devel. Ther.* **12**, 3321–3333 (2018).
28. Yu, H. *et al.* The effects of novel chitosan-targeted gemcitabine nanomedicine mediating cisplatin on epithelial

mesenchymal transition, invasion and metastasis of pancreatic cancer cells. *Biomed. Pharmacother.* **96**, 650–658 (2017).
29. Chen, Q. *et al.* Drug-induced co-assembly of albumin/catalase as smart nano-theranostics for deep intra-tumoral penetration, hypoxia relieve, and synergistic combination therapy. *J. Control. Release* **263**, 79–89 (2017).
30. Mpekris, F. *et al.* Sonic-hedgehog pathway inhibition normalizes desmoplastic tumor microenvironment to improve chemo- and nanotherapy. *J. Control. Release* **261**, 105–112 (2017).
31. Tsai, M. H., Pan, C. H., Peng, C. L. & Shieh, M. J. Panitumumab-Conjugated Pt-Drug Nanomedicine for Enhanced Efficacy of Combination Targeted Chemotherapy against Colorectal Cancer. *Adv. Healthc. Mater.* **6**, 1–10 (2017).
32. Fan, Y. P. *et al.* MiR-375 and Doxorubicin Co-delivered by Liposomes for Combination Therapy of Hepatocellular Carcinoma. *Mol. Ther. - Nucleic Acids* **7**, 181–189 (2017).
33. Zhou, F. *et al.* Programmed Multiresponsive Vesicles for Enhanced Tumor Penetration and Combination Therapy of Triple-Negative Breast Cancer. *Adv. Funct. Mater.* **27**, 1606530 (2017).
34. Fu, X., Wang, X., Zhou, S. & Zhang, Y. IONP-doped nanoparticles for highly effective nir-controlled drug release and combination tumor therapy. *Int. J. Nanomedicine* **12**, 3751–3766 (2017).
35. Zhang, B. *et al.* Efficient co-delivery of immiscible hydrophilic/hydrophobic chemotherapeutics by lipid emulsions for improved treatment of cancer. *Int. J. Nanomedicine* **12**, 2871–2886 (2017).
36. Chen, F. yan *et al.* Development of a hybrid paclitaxel-loaded arsenite nanoparticle (HPAN) delivery system for synergistic combined therapy of paclitaxel-resistant cancer. *J. Nanoparticle Res.* **19**, 155 (2017).
37. Zhai, S., Hu, X., Hu, Y., Wu, B. & Xing, D. Visible light-induced crosslinking and physiological stabilization of diselenide-rich nanoparticles for redox-responsive drug release and combination chemotherapy. *Biomaterials* **121**, 41–54 (2017).
38. Cai, Y. *et al.* Supramolecular 'Trojan Horse' for Nuclear Delivery of Dual Anticancer Drugs. *J. Am. Chem. Soc.* **139**, 2876–2879 (2017).
39. Ni, S., Qiu, L., Zhang, G., Zhou, H. & Han, Y. Lymph cancer chemotherapy: Delivery of doxorubicin-gemcitabine prodrug and vincristine by nanostructured lipid carriers. *Int. J. Nanomedicine* **12**, 1565–1576 (2017).
40. Shin, D. H. & Kwon, G. S. Epothilone B-based 3-in-1 polymeric micelle for anticancer drug therapy. *Int. J. Pharm.* **518**, 307–311 (2017).
41. Ruttala, H. B. *et al.* Molecularly targeted co-delivery of a histone deacetylase inhibitor and paclitaxel by lipid-protein hybrid nanoparticles for synergistic combinational chemotherapy. *Oncotarget* **8**, 14925–14940 (2017).
42. Di, H. *et al.* Doxorubicin- and cisplatin-loaded nanostructured lipid carriers for breast cancer combination chemotherapy. *Drug Dev. Ind. Pharm.* **42**, 2038–2043 (2016).
43. Zhang, X., Li, J. & Yan, M. Targeted hepatocellular carcinoma therapy: transferrin modified, self-assembled polymeric nanomedicine for co-delivery of cisplatin and doxorubicin. *Drug Dev. Ind. Pharm.* **42**, 1590–1599 (2016).
44. Goldman, A. *et al.* Rationally Designed 2-in-1 Nanoparticles Can Overcome Adaptive Resistance in Cancer. *ACS Nano* **10**, 5823–5834 (2016).
45. Liu, Q. *et al.* Co-delivery of baicalein and doxorubicin by hyaluronic acid decorated nanostructured lipid carriers for breast cancer therapy. *Drug Deliv.* **23**, 1364–1368 (2016).
46. Wang, X. *et al.* A nanomedicine based combination therapy based on QLPVM peptide functionalized liposomal tamoxifen and doxorubicin against Luminal A breast cancer. *Nanomedicine Nanotechnology, Biol. Med.* **12**, 387–397 (2016).
47. Xu, B. *et al.* Polymeric Nanomedicine for Combined Gene/Chemotherapy Elicits Enhanced Tumor Suppression. *Mol. Pharm.* **13**, 663–676 (2016).
48. Hung, C. C. *et al.* Active tumor permeation and uptake of surface charge-switchable theranostic nanoparticles for imaging-guided photothermal/chemo combinatorial therapy. *Theranostics* **6**, 302–317 (2016).
49. Qu, C. Y. *et al.* Engineering of lipid prodrug-based, hyaluronic acid-decorated nanostructured lipid carriers platform for 5-fluorouracil and cisplatin combination gastric cancer therapy. *Int. J. Nanomedicine* **10**, 3911–3920 (2015).
50. Wang, W., Xi, M., Duan, X., Wang, Y. & Kong, F. E. Delivery of baicalein and paclitaxel using self-assembled nanoparticles: Synergistic antitumor effect in vitro and in vivo. *Int. J. Nanomedicine* **10**, 3737–3750 (2015).
51. Sun, R. *et al.* Co-delivery of all-trans-retinoic acid and doxorubicin for cancer therapy with synergistic inhibition of cancer stem cells. *Biomaterials* **37**, 405–414 (2015).
52. Lukianova-Hleb, E. Y. *et al.* Safety and efficacy of quadrapeutics versus chemoradiation in head and neck carcinoma xenograft model. *Am. J. Cancer Res.* **5**, 3534–3547 (2015).
53. Park, S. *et al.* Dual pH-sensitive oxidative stress generating micellar nanoparticles as a novel anticancer therapeutic agent. *J. Control. Release* **196**, 19–27 (2014).
54. Drinberg, V., Bitcover, R., Rajchenbach, W. & Peer, D. Modulating cancer multidrug resistance by sertraline in combination with a nanomedicine. *Cancer Lett.* **354**, 290–298 (2014).
55. Li, Y. *et al.* A smart and versatile theranostic nanomedicine platform based on nanoporphyrin. *Nat. Commun.* **5**, 4712 (2014).
56. Wang, H. *et al.* Doxorubicin and lapatinib combination nanomedicine for treating resistant breast cancer. *Mol. Pharm.* **11**, 2600–2611 (2014).
57. Liu, Y., Fang, J., Kim, Y. J., Wong, M. K. & Wang, P. Codelivery of doxorubicin and paclitaxel by cross-linked

multilamellar liposome enables synergistic antitumor activity. *Mol. Pharm.* **11**, 1651–1661 (2014).
58. Senanayake, T. H. *et al.* Encapsulation of poorly soluble drugs in polymer-drug conjugates: Effect of dual-drug nanoformulations on cancer therapy. *Pharm. Res.* **31**, 1605–1615 (2014).
59. Talelli, M. *et al.* Intrinsically active nanobody-modified polymeric micelles for tumor-targeted combination therapy. *Biomaterials* **34**, 1255–1260 (2013).
60. Guo, L. *et al.* Combination of TRAIL and Actinomycin D liposomes enhances antitumor effect in non-small cell lung cancer. *Int. J. Nanomedicine* **7**, 1449–1460 (2012).
61. Ren, Y. *et al.* Multifunctional magnetic Fe3O4 nanoparticles combined with chemotherapy and hyperthermia to overcome multidrug resistance. *Int. J. Nanomedicine* **7**, 2261–2269 (2012).
62. Lu, D., Wientjes, M. G., Lu, Z. & Au, J. L. S. Tumor priming enhances delivery and efficacy of nanomedicines. *J. Pharmacol. Exp. Ther.* **322**, 80–88 (2007).
63. Zhang, Y. *et al.* Surfactant-stripped J-aggregates of azaBODIPY derivatives: All-in-one phototheranostics in the second near infrared window. *J. Control. Release* **326**, 256–264 (2020).
64. Jang, Y. *et al.* Development of an ultrasound triggered nanomedicine-microbubble complex for chemo-photodynamic-gene therapy. *Nanomedicine Nanotechnology, Biol. Med.* **27**, 102194 (2020).
65. Liu, M. *et al.* Co-delivery of doxorubicin and DNAzyme using ZnO@polydopamine core-shell nanocomposites for chemo/gene/photothermal therapy. *Acta Biomater.* **110**, 242–253 (2020).
66. Liu, X. *et al.* Mitochondrion- and nucleus-acting polymeric nanoagents for chemo-photothermal combination therapy. *Sci. China Mater.* **63**, 851–863 (2020).
67. Wu, Z. *et al.* Synergistic action of doxorubicin and 7-Ethyl-10-hydroxycamptothecin polyphosphorylcholine polymer prodrug. *Colloids Surfaces B Biointerfaces* **189**, 110741 (2020).
68. Li, Z. *et al.* Near-infrared/pH dual-responsive nanocomplexes for targeted imaging and chemo/gene/photothermal tri-therapies of non-small cell lung cancer. *Acta Biomater.* **107**, 242–259 (2020).
69. Lee, R. *et al.* Hyaluronic Acid-Decorated Glycol Chitosan Nanoparticles for pH-Sensitive Controlled Release of Doxorubicin and Celecoxib in Nonsmall Cell Lung Cancer. *Bioconjug. Chem.* **31**, 923–932 (2020).
70. Cong, Z. *et al.* Size-Transformable Hyaluronan Stacked Self-Assembling Peptide Nanoparticles for Improved Transcellular Tumor Penetration and Photo-Chemo Combination Therapy. *ACS Nano* **14**, 1958–1970 (2020).
71. Baldwin, P. *et al.* Nanoformulation of Talazoparib Increases Maximum Tolerated Doses in Combination With Temozolomide for Treatment of Ewing Sarcoma. *Front. Oncol.* **9**, 1–9 (2019).
72. Lee, H. *et al.* Combination of chemotherapy and photodynamic therapy for cancer treatment with sonoporation effects. *J. Control. Release* **283**, 190–199 (2018).
73. Wei, G., Yang, G., Wei, B., Wang, Y. & Zhou, S. Near-infrared light switching nitric oxide nanoemitter for triple-combination therapy of multidrug resistant cancer. *Acta Biomater.* **100**, 365–377 (2019).
74. Dai, Y. *et al.* Multifunctional Theranostic Liposomes Loaded with a Hypoxia-Activated Prodrug for Cascade-Activated Tumor Selective Combination Therapy. *ACS Appl. Mater. Interfaces* **11**, 39410–39423 (2019).
75. Feng, Y. *et al.* Magnetic Manganese Oxide Sweetgum-Ball Nanospheres with Large Mesopores Regulate Tumor Microenvironments for Enhanced Tumor Nanotheranostics. *ACS Appl. Mater. Interfaces* **11**, 37461–37470 (2019).
76. Ji, Y. *et al.* Tandem activated photodynamic and chemotherapy: Using pH-Sensitive nanosystems to realize different tumour distributions of photosensitizer/prodrug for amplified combination therapy. *Biomaterials* 219, 119393 (2019).
77. Ramesh, A., Natarajan, S. K., Nandi, D. & Kulkarni, A. Dual Inhibitors-Loaded Nanotherapeutics that Target Kinase Signaling Pathways Synergize with Immune Checkpoint Inhibitor. *Cell. Mol. Bioeng.* **12**, 357–373 (2019).
78. Li, Y. *et al.* A Simple Glutathione-Responsive Turn-On Theranostic Nanoparticle for Dual-Modal Imaging and Chemo-Photothermal Combination Therapy. *Nano Lett.* **19**, 5806–5817 (2019).
79. Ding, Y., Du, C., Qian, J. & Dong, C. M. NIR-Responsive Polypeptide Nanocomposite Generates NO Gas, Mild Photothermia, and Chemotherapy to Reverse Multidrug-Resistant Cancer. *Nano Lett.* **19**, 4362–4370 (2019).
80. Zhu, R. *et al.* Cancer-Selective Bioreductive Chemotherapy Mediated by Dual Hypoxia-Responsive Nanomedicine upon Photodynamic Therapy-Induced Hypoxia Aggravation. *Biomacromolecules* **20**, 2649–2656 (2019).
81. Zhang, X. *et al.* Highly stable near-infrared dye conjugated cerasomes for fluorescence imaging-guided synergistic chemo-photothermal therapy of colorectal cancer. *Biomater. Sci.* **7**, 2873–2888 (2019).
82. Dai, Y. *et al.* Toxic Reactive Oxygen Species Enhanced Synergistic Combination Therapy by Self-Assembled Metal-Phenolic Network Nanoparticles. *Adv. Mater.* **30**, 1704877 (2018).
83. Hao, J. *et al.* Temporary suppression the sequestrated function of host macrophages for better nanoparticles tumor delivery. *Drug Deliv.* **25**, 1289–1301 (2018).
84. Lim, W. Q., Yang, G., Phua, S. Z. F., Chen, H. & Zhao, Y. Self-Assembled Oxaliplatin(IV) Prodrug-Porphyrin Conjugate for Combinational Photodynamic Therapy and Chemotherapy. *ACS Appl. Mater. Interfaces* **11**, 16391–16401 (2019).
85. Liu, X. *et al.* Water-Responsive Hybrid Nanoparticles Codelivering ICG and DOX Effectively Treat Breast Cancer via Hyperthermia-aided DOX Functionality and Drug Penetration. *Adv. Healthc. Mater.* **8**, 1–15 (2019).
86. Li, H. *et al.* Prussian blue-modified ferritin nanoparticles for effective tumor chemo-photothermal combination therapy via enhancing reactive oxygen species production. *J. Biomater. Appl.* **33**, 1202–1213 (2019).

87. Xu, Y. *et al.* A switchable NO-releasing nanomedicine for enhanced cancer therapy and inhibition of metastasis. *Nanoscale* **11**, 5474–5488 (2019).
88. Liu, J. *et al.* Effective co-encapsulation of doxorubicin and irinotecan for synergistic therapy using liposomes prepared with triethylammonium sucrose octasulfate as drug trapping agent. *Int. J. Pharm.* **557**, 264–272 (2019).
89. Prasad, C. & Banerjee, R. Ultrasound-triggered spatiotemporal delivery of topotecan and curcumin as combination therapy for cancer. *J. Pharmacol. Exp. Ther.* **370**, 876–893 (2019).
90. Du, X. *et al.* Glucose-responsive mesoporous silica nanoparticles to generation of hydrogen peroxide for synergistic cancer starvation and chemistry therapy. *Int. J. Nanomedicine* **14**, 2233–2251 (2019).
91. Zhang, L. *et al.* Improving Drug Delivery of Micellar Paclitaxel against Non-Small Cell Lung Cancer by Coloading Itraconazole as a Micelle Stabilizer and a Tumor Vascular Manipulator. *Small* **14**, 1–13 (2018).
92. Wang, X. shuai *et al.* A highly integrated precision nanomedicine strategy to target esophageal squamous cell cancer molecularly and physically. *Nanomedicine Nanotechnology, Biol. Med.* **14**, 2103–2114 (2018).
93. Li, Q. *et al.* Hyaluronic Acid-Methotrexate Conjugates Coated Magnetic Polydopamine Nanoparticles for Multimodal Imaging-Guided Multistage Targeted Chemo-Photothermal Therapy. *Mol. Pharm.* **15**, 4049–4062 (2018).
94. Yang, J. *et al.* NIR-controlled morphology transformation and pulsatile drug delivery based on multifunctional phototheranostic nanoparticles for photoacoustic imaging-guided photothermal-chemotherapy. *Biomaterials* **176**, 1–12 (2018).
95. Zhang, L. *et al.* Dual pH/reduction-responsive hybrid polymeric micelles for targeted chemo-photothermal combination therapy. *Acta Biomater.* **75**, 371–385 (2018).
96. Morton, S. W. *et al.* A nanoparticle-based combination chemotherapy delivery system for enhanced tumor killing by dynamic rewiring of signaling pathways. *Sci. Signal.* **7**, 1–11 (2014).
97. Liu, Z. *et al.* A novel GSH responsive poly(alpha-lipoic acid) nanocarrier bonding with the honokiol-DMXAA conjugate for combination therapy. *Sci. China Mater.* **63**, 307–315 (2020).
98. Wang, H. *et al.* Nanoscale covalent organic polymers as a biodegradable nanomedicine for chemotherapy-enhanced photodynamic therapy of cancer. *Nano Res.* **11**, 3244–3257 (2018).
99. Liu, Y. *et al.* Combination drug delivery via multilamellar vesicles enables targeting of tumor cells and tumor vasculature. *Biotechnol. Bioeng.* **115**, 1403–1415 (2018).
100. Ni, Q. *et al.* In Situ shRNA Synthesis on DNA–Polylactide Nanoparticles to Treat Multidrug Resistant Breast Cancer. *Adv. Mater.* **30**, 1–10 (2018).
101. Qi, W., Yan, J., Sun, H. & Wang, H. Nanocomposite plasters for the treatment of superficial tumors by chemo-photothermal combination therapy. *Int. J. Nanomedicine* **13**, 6235–6247 (2018).
102. Guan, Z. *et al.* Thermosensitive micellar hydrogel for enhanced anticancer therapy through redox modulation mediated combinational effects. *RSC Adv.* **7**, 34755–34762 (2017).
103. Yang, X., Shi, X., Ji, J. & Zhai, G. Development of redox-responsive theranostic nanoparticles for near-infrared fluorescence imaging-guided photodynamic/chemotherapy of tumor. *Drug Deliv.* **25**, 780–796 (2018).
104. Hameed, S. *et al.* Self-assembly of porphyrin-grafted lipid into nanoparticles encapsulating doxorubicin for synergistic chemo-photodynamic therapy and fluorescence imaging. *Theranostics* **8**, 5501–5518 (2018).
105. Hu, D. R. *et al.* Oxygen-generating hybrid polymeric nanoparticles with encapsulated doxorubicin and chlorin e6 for trimodal imaging-guided combined chemo-photodynamic therapy. *Theranostics* **8**, 1558–1574 (2018).
106. Zhai, Y. *et al.* Traceable bioinspired nanoparticle for the treatment of metastatic breast cancer via NIR-trigged intracellular delivery of methylene blue and cisplatin. *Adv. Mater.* **30**, 1–10 (2018).
107. Luo, Z. *et al.* Tumor-targeted hybrid protein oxygen carrier to simultaneously enhance hypoxia-dampened chemotherapy and photodynamic therapy at a single dose. *Theranostics* **8**, 3584–3596 (2018).
108. Fan, J. X. *et al.* A Metal-Polyphenol Network Coated Nanotheranostic System for Metastatic Tumor Treatments. *Small* **13**, 1702714 (2017).
109. Yang, G. *et al.* Hollow MnO2 as a tumor-microenvironment-responsive biodegradable nano-platform for combination therapy favoring antitumor immune responses. *Nat. Commun.* **8**, 902 (2017).
110. Sun, W. *et al.* Codelivery of sorafenib and GPC3 siRNA with PEI-modified liposomes for hepatoma therapy. *Biomater. Sci.* **5**, 2468–2479 (2017).
111. Kaittanis, C. *et al.* Targetable Clinical Nanoparticles for Precision Cancer Therapy Based on Disease-Specific Molecular Inflection Points. *Nano Lett.* **17**, 7160–7168 (2017).
112. Zhao, P. *et al.* MiR-375 delivered by lipid-coated doxorubicin-calcium carbonate nanoparticles overcomes chemoresistance in hepatocellular carcinoma. *Nanomedicine Nanotechnology, Biol. Med.* **13**, 2507–2516 (2017).
113. Wen, Y. *et al.* Carrier-free, self-assembled pure drug nanorods composed of 10-hydroxycamptothecin and chlorin e6 for combinatorial chemo-photodynamic antitumor therapy: In vivo. *Nanoscale* **9**, 14347–14356 (2017).
114. Yang, X. *et al.* Sub-100 nm, long tumor retention SN-38-loaded photonic micelles for tri-modal cancer therapy. *J. Control. Release* **261**, 297–306 (2017).
115. Li, Y. *et al.* Chemotherapeutic drug-photothermal agent co-self-assembling nanoparticles for near-infrared fluorescence and photoacoustic dual-modal imaging-guided chemo-photothermal synergistic therapy. *J. Control. Release* **258**, 95–107 (2017).

116. Baabur-Cohen *et al.* In vivo comparative study of distinct polymeric architectures bearing a combination of paclitaxel and doxorubicin at a synergistic ratio. *J. Control. Release* **10**, 118-131 (2016)
117. Jin, W. *et al.* Lanthanide-integrated supramolecular polymeric nanoassembly with multiple regulation characteristics for multidrug-resistant cancer therapy. *Biomaterials* **129**, 83–97 (2017).
118. Yang, H. *et al.* Chemo-photodynamic combined gene therapy and dual-modal cancer imaging achieved by pH-responsive alginate/chitosan multilayer-modified magnetic mesoporous silica nanocomposites. *Biomater. Sci.* **5**, 1001–1013 (2017).
119. Wang, H. *et al.* Redox-Activatable ATP-Depleting Micelles with Dual Modulation Characteristics for Multidrug-Resistant Cancer Therapy. *Adv. Healthc. Mater.* **6**, 1–14 (2017).
120. Wang, H. *et al.* Precise Engineering of Prodrug Cocktails into Single Polymeric Nanoparticles for Combination Cancer Therapy: Extended and Sequentially Controllable Drug Release. *ACS Appl. Mater. Interfaces* **9**, 10567–10576 (2017).
121. Peng, C. L. *et al.* A novel temperature-responsive micelle for enhancing combination therapy. *Int. J. Nanomedicine* **11**, 3357–3369 (2016).
122. Lu, Z., Su, J., Li, Z., Zhan, Y. & Ye, D. Hyaluronic acid-coated, prodrug-based nanostructured lipid carriers for enhanced pancreatic cancer therapy. *Drug Dev. Ind. Pharm.* **43**, 160–170 (2017).
123. Wang, H. *et al.* Cancer nanomedicines stabilized by π-π stacking between heterodimeric prodrugs enable exceptionally high drug loading capacity and safer delivery of drug combinations. *Theranostics* **7**, 3638–3652 (2017).
124. Wang, K. *et al.* Aggregation Induced Emission Fluorogens Based Nanotheranostics for Targeted and Imaging-Guided Chemo-Photothermal Combination Therapy. *Small* **12**, 6568–6575 (2016).
125. Chen, B. *et al.* Comprehensively priming the tumor microenvironment by cancer-associated fibroblast-targeted liposomes for combined therapy with cancer cell-targeted chemotherapeutic drug delivery system. *J. Control. Release* **241**, 68–80 (2016).
126. Li, W. T. *et al.* Mild photothermal therapy/photodynamic therapy/chemotherapy of breast cancer by Lyp-1 modified Docetaxel/IR820 Co-loaded micelles. *Biomaterials*. **106**, 119-133 (2016).
127. Murugan, C. *et al.* Combinatorial nanocarrier based drug delivery approach for amalgamation of anti-tumor agents in bresat cancer cells: An improved nanomedicine strategies. *Sci. Rep.* **6**, 1–17 (2016).
128. Lu, Y. *et al.* Exploiting in situ antigen generation and immune modulation to enhance chemotherapy response in advanced melanoma: A combination nanomedicine approach. *Cancer Lett.* **379**, 32–38 (2016).
129. Yan, J. *et al.* Targeted nanomedicine for prostate cancer therapy: docetaxel and curcumin co-encapsulated lipid–polymer hybrid nanoparticles for the enhanced anti-tumor activity in vitro and in vivo. *Drug Deliv.* **23**, 1757–1762 (2016).
130. Kim, J. *et al.* Multifunctional theranostic nanomedicine for photoacoustic imaging-guided combination tumor treatment. *J. Nanosci. Nanotechnol.* **16**, 11903–11910 (2016).
131. Zhang, J. *et al.* Self-Monitoring and Self-Delivery of Photosensitizer-Doped Nanoparticles for Highly Effective Combination Cancer Therapy in Vitro and in Vivo. *ACS Nano* **9**, 9741–9756 (2015).
132. Kim, Y. J. *et al.* Co-Eradication of Breast Cancer Cells and Cancer Stem Cells by Cross-Linked Multilamellar Liposomes Enhances Tumor Treatment. *Mol. Pharm.* **12**, 2811–2822 (2015).
133. Yao, J. *et al.* On-demand CO release for amplification of chemotherapy by MOF functionalized magnetic carbon nanoparticles with NIR irradiation. *Biomaterials* **195**, 51–62 (2019).
134. Chen, Q. *et al.* Drug-induced self-assembly of modified albumins as nano-theranostics for tumor-targeted combination therapy. *ACS Nano* **9**, 5223–5233 (2015).
135. Li, S. Y. *et al.* Combination therapy with epigenetic-targeted and chemotherapeutic drugs delivered by nanoparticles to enhance the chemotherapy response and overcome resistance by breast cancer stem cells. *J. Control. Release* **205**, 7–14 (2015).
136. Yang, Q. *et al.* Polymeric nanomedicine for tumor-targeted combination therapy to elicit synergistic genotoxicity against prostate cancer. *ACS Appl. Mater. Interfaces* **7**, 6661–6673 (2015).
137. He, C., Liu, D. & Lin, W. Self-assembled core-shell nanoparticles for combined chemotherapy and photodynamic therapy of resistant head and neck cancers. *ACS Nano* **9**, 991–1003 (2015).
138. Markovsky, E., Baabur-Cohen, H. & Satchi-Fainaro, R. Anticancer polymeric nanomedicine bearing synergistic drug combination is superior to a mixture of individually-conjugated drugs. *J. Control. Release* **187**, 145–157 (2014).
139. Ma, Y. *et al.* Combinational delivery of hydrophobic and hydrophilic anticancer drugs in single nanoemulsions to treat MDR in cancer. *Mol. Pharm.* **11**, 2623–2630 (2014).
140. Pandey, A. *et al.* Sequential application of a cytotoxic nanoparticle and a PI3K inhibitor enhances antitumor efficacy. *Cancer Res.* **74**, 675–685 (2014).
141. Tang, J. *et al.* Antitumor effects of MsurvivinT34A–CaPi complex-embedded PLGA nanoparticles in combination with Doxil in mice. *J. Nanoparticle Res.* **16**, 2682 (2014).
142. Tai, W., Mo, R., Lu, Y., Jiang, T. & Gu, Z. Folding graft copolymer with pendant drug segments for co-delivery of anticancer drugs. *Biomaterials* **35**, 7194–7203 (2014).

143. Wang, H., Wu, Y., Zhao, R. & Nie, G. Engineering the assemblies of biomaterial nanocarriers for delivery of multiple theranostic agents with enhanced antitumor efficacy. *Adv. Mater.* **25**, 1616–1622 (2013).
144. Huis In 'T Veld, R., Storm, G., Hennink, W. E., Kiessling, F. & Lammers, T. Macromolecular nanotheranostics for multimodal anticancer therapy. *Nanoscale* **3**, 4022–4034 (2011).
145. He, Y. *et al.* A pH-responsive dissociable mesoporous silica-based nanoplatform enabling efficient dual-drug co-delivery and rapid clearance for cancer therapy. *Biomater. Sci.* **8**, 3418–3429 (2020).
146. Feng, B. *et al.* Enhancing Triple Negative Breast Cancer Immunotherapy by ICG-Templated Self-Assembly of Paclitaxel Nanoparticles. *Adv. Funct. Mater.* **30**, 1–13 (2020).
147. Li, Y. *et al.* Co-delivery of Poria cocos extract and doxorubicin as an 'all-in-one' nanocarrier to combat breast cancer multidrug resistance during chemotherapy. *Nanomedicine Nanotechnology, Biol. Med.* **23**, 102095 (2020).
148. Sun, J. *et al.* High loading of hydrophobic and hydrophilic agents via small immunostimulatory carrier for enhanced tumor penetration and combinational therapy. *Theranostics* **10**, 1136–1150 (2020).
149. Xie, X. *et al.* Polymeric Hybrid Nanomicelles for Cancer Theranostics: An Efficient and Precise Anticancer Strategy for the Codelivery of Doxorubicin/miR-34a and Magnetic Resonance Imaging. *ACS Appl. Mater. Interfaces* **11**, 43865–43878 (2019).
150. Ding, Y., Du, C., Qian, J. & Dong, C. M. Zwitterionic polypeptide nanomedicine with dual NIR/reduction-responsivity for synergistic cancer photothermal-chemotherapy. *Polym. Chem.* **10**, 4825–4836 (2019).
151. Niu, S. *et al.* A novel chitosan-based nanomedicine for multi-drug resistant breast cancer therapy. *Chem. Eng. J.* **369**, 134–149 (2019).
152. Wang, Z. *et al.* Light-activatable dual prodrug polymer nanoparticle for precise synergistic chemotherapy guided by drug-mediated computed tomography imaging. *Acta Biomater.* **94**, 459–468 (2019).
153. Zhang, L. *et al.* Enhancing solid tumor therapy with sequential delivery of dexamethasone and docetaxel engineered in a single carrier to overcome stromal resistance to drug delivery. *J. Control. Release* **294**, 1–16 (2019).
154. Li, Q. *et al.* Co-delivery of chlorin e6 and doxorubicin using PEGylated hollow nanocapsules for â€ all-in-one' tumor theranostics. *Nanomedicine* **14**, 2273–2292 (2019).
155. Deng, J. *et al.* Hypoxia- and singlet oxygen-responsive chemo-photodynamic Micelles featured with glutathione depletion and aldehyde production. *Biomater. Sci.* **7**, 429–441 (2019).
156. Yu, S. *et al.* Co-delivery of paclitaxel and PLK1-targeted siRNA using aptamer-functionalized cationic liposome for synergistic anti-breast cancer effects in vivo. *J. Biomed. Nanotechnol.* **15**, 1135–1148 (2019).
157. Jin, H. *et al.* Targeting lipid metabolism to overcome EMT-associated drug resistance via integrin β3/FAK pathway and tumor-associated macrophage repolarization using legumain-activatable delivery. *Theranostics* **9**, 265–278 (2019).
158. Han, W. *et al.* A nanomedicine approach enables co-delivery of cyclosporin A and gefitinib to potentiate the therapeutic efficacy in drug-resistant lung cancer. *Signal Transduct. Target. Ther.* **3**, 1–10 (2018).
159. Yin, W. *et al.* Remodeling Tumor-Associated Macrophages and Neovascularization Overcomes EGFRT790M-Associated Drug Resistance by PD-L1 Nanobody-Mediated Codelivery. *Small* **14**, 1–13 (2018).
160. Rastegar, R. *et al.* Evaluation of a novel biocompatible magnetic nanomedicine based on beta-cyclodextrin, loaded doxorubicin-curcumin for overcoming chemoresistance in breast cancer. *Artif. Cells, Nanomedicine Biotechnol.* **46**, 207–216 (2018).
161. Zhang, L. *et al.* Dual drug delivery and sequential release by amphiphilic Janus nanoparticles for liver cancer theranostics. *Biomaterials* **181**, 113–125 (2018).
162. Sun, N. *et al.* Cargo-Free Nanomedicine with pH Sensitivity for Codelivery of DOX Conjugated Prodrug with SN38 to Synergistically Eradicate Breast Cancer Stem Cells. *Mol. Pharm.* **15**, 3343–3355 (2018).
163. Liu, J. *et al.* A DNA-Based Nanocarrier for Efficient Gene Delivery and Combined Cancer Therapy. *Nano Lett.* **18**, 3328–3334 (2018).
164. Zhang, Y. *et al.* Self-Stabilized Hyaluronate Nanogel for Intracellular Codelivery of Doxorubicin and Cisplatin to Osteosarcoma. *Adv. Sci.* **5**, 1-12 (2018).
165. Yao, M. *et al.* Enzyme Degradable Hyperbranched Polyphosphoester Micellar Nanomedicines for NIR Imaging-Guided Chemo-Photothermal Therapy of Drug-Resistant Cancers. *Biomacromolecules* **19**, 1130–1141 (2018).
166. Zhao, X. *et al.* Precision design of nanomedicines to restore gemcitabine chemosensitivity for personalized pancreatic ductal adenocarcinoma treatment. *Biomaterials* **158**, 44–55 (2018).
167. Zhou, M., Zhang, X., Xu, X., Chen, X. & Zhang, X. Doxorubicin@Bcl-2 siRNA Core@Shell nanoparticles for synergistic anticancer chemotherapy. *ACS Appl. Bio Mater.* **1**, 289–297 (2018).
168. Sun, C. Y. *et al.* Cascade-amplifying synergistic effects of chemophotodynamic therapy using ROS-responsive polymeric nanocarriers. *Theranostics* **8**, 2939–2953 (2018).
169. Lv, Y. *et al.* Targeting intracellular MMPs efficiently inhibits tumor metastasis and angiogenesis. *Theranostics* **8**, 2830–2845 (2018).
170. Cheng, W. *et al.* A drug-self-gated and tumor microenvironment-responsive mesoporous silica vehicle: 'four-in-one' versatile nanomedicine for targeted multidrug-resistant cancer therapy. *Nanoscale* **9**, 17063–17073 (2017).
171. Wei, Y. *et al.* Thermosensitive Liposomal Codelivery of HSA-Paclitaxel and HSA-Ellagic Acid Complexes for

Enhanced Drug Perfusion and Efficacy Against Pancreatic Cancer. *ACS Appl. Mater. Interfaces* **9**, 25138–25151 (2017).
172. Li, Y. *et al.* Co-delivery of microRNA-21 antisense oligonucleotides and gemcitabine using nanomedicine for pancreatic cancer therapy. *Cancer Sci.* **108**, 1493–1503 (2017).
173. Sun, L. *et al.* Core-shell hierarchical mesostructured silica nanoparticles for gene/chemo-synergetic stepwise therapy of multidrug-resistant cancer. *Biomaterials* **133**, 219–228 (2017).
174. Shen, X. *et al.* Luminescent/magnetic PLGA-based hybrid nanocomposites: A smart nanocarrier system for targeted codelivery and dual-modality imaging in cancer theranostics. *Int. J. Nanomedicine* **12**, 4299–4322 (2017).
175. Zheng, Y., Su, C., Zhao, L. & Shi, Y. Chitosan nanoparticle-mediated co-delivery of shAtg-5 and gefitinib synergistically promoted the efficacy of chemotherapeutics through the modulation of autophagy. *J. Nanobiotechnology* **15**, 1–11 (2017).
176. Li, C., Ge, X. & Wang, L. Construction and comparison of different nanocarriers for co-delivery of cisplatin and curcumin: A synergistic combination nanotherapy for cervical cancer. *Biomed. Pharmacother.* **86**, 628–636 (2017).
177. Kotcherlakota, R. *et al.* Engineered fusion protein-loaded gold nanocarriers for targeted co-delivery of doxorubicin and erbB2-siRNA in human epidermal growth factor receptor-2+ ovarian cancer. *J. Mater. Chem. B* **5**, 7082–7098 (2017).
178. Li, Y. *et al.* Dual-acting, function-responsive, and high drug payload nanospheres for combining simplicity and efficacy in both self-targeted multi-drug co-delivery and synergistic anticancer effect. *Int. J. Pharm.* **512**, 194–203 (2016).
179. Li, J. *et al.* A nanoparticle carrier for co-delivery of gemcitabine and small interfering rna in pancreatic cancer therapy. *J. Biomed. Nanotechnol.* **12**, 1654–1666 (2016).
180. Zhang, R. *et al.* Carrier-Free, Chemophotodynamic Dual Nanodrugs via Self-Assembly for Synergistic Antitumor Therapy. *ACS Appl. Mater. Interfaces* **8**, 13262–13269 (2016).
181. Shu, C., Sabi-Mouka, E. M. B., Yang, W., Li, Z. & Ding, L. Effects of paclitaxel (PTX) prodrug-based self-assembly peptide hydrogels combined with suberoylanilide hydroxamic acid (SAHA) for PTX-resistant cancer and synergistic antitumor therapy. *RSC Adv.* **6**, 100765–100771 (2016).
182. Li, Y. *et al.* Self-Targeted, Shape-Assisted, and Controlled-Release Self-Delivery Nanodrug for Synergistic Targeting/Anticancer Effect of Cytoplasm and Nucleus of Cancer Cells. *ACS Appl. Mater. Interfaces* **7**, 25553–25559 (2015).
183. Chen, D., Wang, G., Song, W. & Zhang, Q. Novel CD44 receptor targeting multifunctional "nano-eggs" based on double pH-sensitive nanoparticles for co-delivery of curcumin and paclitaxel to cancer cells and cancer stem cells. *J. Nanoparticle Res.* **17**, 1487–1508 (2015).
184. Jiang, T., Mo, R., Bellotti, A., Zhou, J. & Gu, Z. Gel-liposome-mediated co-delivery of anticancer membrane-associated proteins and small-molecule drugs for enhanced therapeutic efficacy. *Adv. Funct. Mater.* **24**, 2295–2304 (2014).
185. Zhang, X. *et al.* Enhancing therapeutic effects of docetaxel-loaded dendritic copolymer nanoparticles by co-treatment with autophagy inhibitor on breast cancer. *Theranostics* **4**, 1085–1095 (2014).
186. Dai, W. *et al.* Spatiotemporally controlled co-Delivery of anti-Vasculature agent and cytotoxic drug by octreotide-Modified stealth liposomes. *Pharm. Res.* **29**, 2902–2911 (2012).
187. Zhou, S. *et al.* Rational design of a minimalist nanoplatform to maximize immunotherapeutic efficacy: Four birds with one stone. *J. Control. Release* **328**, 617–630 (2020).
188. Liu, J. *et al.* Efficiency of Different Treatment Regimens Combining Anti-tumor and Anti-inflammatory Liposomes for Metastatic Breast Cancer. *AAPS PharmSciTech* **21**, 1–12 (2020).
189. Zhang, Y. *et al.* Glioblastoma Therapy Using Codelivery of Cisplatin and Glutathione Peroxidase Targeting siRNA from Iron Oxide Nanoparticles. *ACS Appl. Mater. Interfaces* **12**, 43408–43421 (2020).
190. Ma, Z. *et al.* Dual drug-loaded nano-platform for targeted cancer therapy: Toward clinical therapeutic efficacy of multifunctionality. *J. Nanobiotechnology* **18**, 1–24 (2020).
191. Zhou, Y. *et al.* Bone marrow mesenchymal stem cells-derived exosomes for penetrating and targeted chemotherapy of pancreatic cancer. *Acta Pharm. Sin. B* **10**, 1563–1575 (2020).
192. Pang, J., Xing, H., Sun, Y., Feng, S. & Wang, S. Non-small cell lung cancer combination therapy: Hyaluronic acid modified, epidermal growth factor receptor targeted, pH sensitive lipid-polymer hybrid nanoparticles for the delivery of erlotinib plus bevacizumab. *Biomed. Pharmacother.* **125**, 109861 (2020).
193. Yang, M., Zhang, N., Zhang, T., Yin, X. & Shen, J. Fabrication of doxorubicin-gated mesoporous polydopamine nanoplatforms for multimode imaging-guided synergistic chemophotothermal therapy of tumors. *Drug Deliv.* **27**, 367–377 (2020).
194. Chen, J. *et al.* Spatiotemporally Targeted Nanomedicine Overcomes Hypoxia-Induced Drug Resistance of Tumor Cells after Disrupting Neovasculature. *Nano Lett.* **20**, 6191–6198 (2020).
195. Lin, X. *et al.* Multifunctional theranostic nanosystems enabling photothermal-chemo combination therapy of triple-stimuli-responsive drug release with magnetic resonance imaging. *Biomater. Sci.* **8**, 1875–1884 (2020).
196. Lei, F. *et al.* Nanoscale platform for delivery of active IRINOX to combat pancreatic cancer. *J. Control. Release* **330**, 1229–1243 (2021).

197. Deng, L. *et al.* Novel t7-modified ph-responsive targeted nanosystem for co-delivery of docetaxel and curcumin in the treatment of esophageal cancer. *Int. J. Nanomedicine* **15**, 7745–7762 (2020).
198. Zhang, P. *et al.* A Multistage Cooperative Nanoplatform Enables Intracellular Co-Delivery of Proteins and Chemotherapeutics for Cancer Therapy. *Adv. Mater.* **32**, 1–11 (2020).
199. Babaei, M. *et al.* Targeted rod-shaped mesoporous silica nanoparticles for the co-delivery of camptothecin and survivin shRNA in to colon adenocarcinoma in vitro and in vivo. *Eur. J. Pharm. Biopharm.* **156**, 84–96 (2020).
200. Li, M. *et al.* Synergistic Antitumor Effects of Doxorubicin-Loaded Carboxymethyl Cellulose Nanoparticle in Combination with Endostar for Effective Treatment of Non-Small-Cell Lung Cancer. *Adv. Healthc. Mater.* **3**, 1877–1888 (2014).
201. Van Der Meel, R. *et al.* Inhibition of tumor growth by targeted anti-EGFR/IGF-1R nanobullets depends on efficient blocking of cell survival pathways. *Mol. Pharm.* **10**, 3717–3727 (2013).
202. Sun, T. *et al.* Co-delivery of Cu(I) chelator and chemotherapeutics as a new strategy for tumor theranostic. *J. Control. Release* **321**, 483–496 (2020).
203. Wang, H. *et al.* AS1411 Aptamer/Hyaluronic Acid-Bifunctionalized Microemulsion Co-Loading Shikonin and Docetaxel for Enhanced Antiglioma Therapy. *J. Pharm. Sci.* **108**, 3684–3694 (2019).
204. Ji, Y., Li, J., Zhao, J., Shan, S. & Chu, C. C. A light-facilitated drug delivery system from a pseudo-protein/hyaluronic acid nanocomplex with improved anti-tumor effects. *Nanoscale* **11**, 9987–10003 (2019).
205. Li, D. *et al.* Synergistically enhanced anticancer effect of codelivered curcumin and siPlk1 by stimuli-responsive α-lactalbumin nanospheres. *Nanomedicine* **8**, 595–612 (2019).
206. Duan, X. *et al.* Co-delivery of aurora-A inhibitor XY-4 and Bcl-xl siRNA enhances antitumor efficacy for melanoma therapy. *Int. J. Nanomedicine* **13**, 1443–1456 (2018).
207. An, X. *et al.* Rational Design of Multi-Stimuli-Responsive Nanoparticles for Precise Cancer Therapy. *ACS Nano* **10**, 5947–5958 (2016).
208. Tangutoori, S. *et al.* Simultaneous delivery of cytotoxic and biologic therapeutics using nanophotoactivatable liposomes enhances treatment efficacy in a mouse model of pancreatic cancer. *Nanomedicine Nanotechnology, Biol. Med.* **12**, 223–234 (2016).
209. Muthu, M. S., Kutty, R. V., Luo, Z., Xie, J. & Feng, S. S. Theranostic vitamin E TPGS micelles of transferrin conjugation for targeted co-delivery of docetaxel and ultra bright gold nanoclusters. *Biomaterials* **39**, 234–248 (2015).
210. Taratula, O., Kuzmov, A., Shah, M., Garbuzenko, O. B. & Minko, T. Nanostructured lipid carriers as multifunctional nanomedicine platform for pulmonary co-delivery of anticancer drugs and siRNA. *J. Control. Release* **171**, 349–357 (2013).
211. Jose, G., Lu, Y. J., Hung, J. T., Yu, A. L. & Chen, J. P. Co-delivery of cpt-11 and panobinostat with anti-gd2 antibody conjugated immunoliposomes for targeted combination chemotherapy. *Cancers (Basel).* **12**, 1–25 (2020).
212. Elhasany, K. A. *et al.* Combination of magnetic targeting with synergistic inhibition of NF-κB and glutathione via micellar drug nanomedicine enhances its anti-tumor efficacy. *Eur. J. Pharm. Biopharm.* **155**, 162–176 (2020).
213. Ma, Z. *et al.* Pharmacophore hybridisation and nanoscale assembly to discover self-delivering lysosomotropic new-chemical entities for cancer therapy. *Nat. Commun.* **11**,1-12 (2020).
214. Lu, Y. J. *et al.* Injectable thermo-sensitive chitosan hydrogel containing CPT-11-loaded EGFR-targeted graphene oxide and SLP2 shRNA for localized drug/gene delivery in glioblastoma therapy. *Int. J. Mol. Sci.* **21**, 1–29 (2020).
215. Cheng, G. *et al.* Programmed Size-Changeable Nanotheranostic Agents for Enhanced Imaging-Guided Chemo/Photodynamic Combination Therapy and Fast Elimination. *Adv. Mater.* **33**, 1–7 (2021).
216. Cui, H. *et al.* Chemotherapeutic potency stimulated by SNAI1-knockdown based on multifaceted nanomedicine. *J. Control. Release* **337**, 343–355 (2021).
217. Cui, X. *et al.* Multicomponent-assembled nanodiamond hybrids for targeted and imaging guided triple-negative breast cancer therapy: Via a ternary collaborative strategy. *Biomater. Sci.* **9**, 3838–3850 (2021).
218. Ding, Y. *et al.* NO-releasing polypeptide nanocomposites reverse cancer multidrug resistance via triple therapies. *Acta Biomater.* **123**, 335–345 (2021).
219. Gabizon, A. *et al.* Liposome co-encapsulation of anti-cancer agents for pharmacological optimization of nanomedicine-based combination chemotherapy. *Cancer Drug Resist.* **4**, 463–484 (2021).
220. Jeon, I. S. *et al.* Anticancer nanocage platforms for combined immunotherapy designed to harness immune checkpoints and deliver anticancer drugs. *Biomaterials* **270**, 120685 (2021).
221. Jia, F. *et al.* Self-assembled fluorescent hybrid nanoparticles-mediated collaborative lncRNA CCAT1 silencing and curcumin delivery for synchronous colorectal cancer theranostics. *J. Nanobiotechnology* **19**, 1–15 (2021).
222. Jiang, Q. *et al.* NIR-laser-triggered gadolinium-doped carbon dots for magnetic resonance imaging, drug delivery and combined photothermal chemotherapy for triple negative breast cancer. *J. Nanobiotechnology* **19**, 1–15 (2021).
223. Kim, K. R. *et al.* Theranostic potential of biodegradable polymeric nanoparticles with paclitaxel and curcumin against breast carcinoma. *Biomater. Sci.* **9**, 3750–3761 (2021).
224. Li, L. *et al.* Albumin-stabilized layered double hydroxide nanoparticles synergized combination chemotherapy for colorectal cancer treatment. *Nanomedicine Nanotechnology, Biol. Med.* **34**, 102369 (2021).

225. Li, S. *et al.* Precisely engineering a carrier-free hybrid nanoassembly for multimodal DNA damage-augmented photodynamic therapy. *Chem. Eng. J.* **426**, 130838 (2021).
226. Li, Y. *et al.* Multifunctional Size-Expandable Nanomedicines Enhance Tumor Accumulation and Penetration for Synergistic Chemo-Photothermal Therapy. *ACS Appl. Mater. Interfaces* **13**, 46361–46374 (2021).
227. Ma, G. *et al.* Multi-functionalized dendrimers for targeted co-delivery of sorafenib and paclitaxel in liver cancers. *J. Drug Deliv. Sci. Technol.* **63**, (2021).
228. Opoku-Damoah, Y., Zhang, R., Ta, H. T. & Xu, Z. P. Vitamin E-facilitated carbon monoxide pro-drug nanomedicine for efficient light-responsive combination cancer therapy. *Biomater. Sci.* **9**, 6086–6097 (2021).
229. Ren, C. *et al.* Construction of all-in-one peptide nanomedicine with photoacoustic imaging guided mild hyperthermia for enhanced cancer chemotherapy. *Chem. Eng. J.* **405**, 127008 (2021).
230. Singh, D., Singh, P., Pradhan, A., Srivastava, R. & Sahoo, S. K. Reprogramming Cancer Stem-like Cells with Nanoforskolin Enhances the Efficacy of Paclitaxel in Targeting Breast Cancer. *ACS Appl. Bio Mater.* **4**, 3670–3685 (2021).
231. Sun, G., Sun, K. & Sun, J. Combination prostate cancer therapy: Prostate-specific membranes antigen targeted, pH-sensitive nanoparticles loaded with doxorubicin and tanshinone . *Drug Deliv.* **28**, 1132–1140 (2021).
232. Wang, S. *et al.* Photodynamic-Chemodynamic Cascade Reactions for Efficient Drug Delivery and Enhanced Combination Therapy. *Adv. Sci.* **8**, 1–9 (2021).
233. Wang, Y. *et al.* Synergistic Therapy for Cervical Cancer by Codelivery of Cisplatin and JQ1 Inhibiting Plk1-Mutant Trp53 Axis. *Nano Lett.* **21**, 2412–2421 (2021).
234. Xiong, Z. *et al.* A Dual-Responsive Platform Based on Antifouling Dendrimer–CuS Nanohybrids for Enhanced Tumor Delivery and Combination Therapy. *Small Methods* **5**, 1–11 (2021).
235. Xu, P. *et al.* A DM1-doped porous gold nanoshell system for NIR accelerated redox-responsive release and triple modal imaging guided photothermal synergistic chemotherapy. *J. Nanobiotechnology* **19**, 1–19 (2021).
236. Xu, Y., Yao, Y., Wang, L., Chen, H. & Tan, N. Hyaluronic acid coated liposomes Co-delivery of natural cyclic peptide RA-XII and mitochondrial targeted photosensitizer for highly selective precise combined treatment of colon cancer. *Int. J. Nanomedicine* **16**, 4929–4942 (2021).
237. Yang, D. C. *et al.* Singlet Oxygen-Responsive Polymeric Nanomedicine for Light-Controlled Drug Release and Image-Guided Photodynamic-Chemo Combination Therapy. *ACS Appl. Mater. Interfaces* **13**, 33905–33914 (2021).
238. Yang, S. *et al.* Coupling metal organic frameworks with molybdenum disulfide nanoflakes for targeted cancer theranostics. *Biomater. Sci.* **9**, 3306–3318 (2021).
239. Zhang, B. bing *et al.* Lipid/PAA-coated mesoporous silica nanoparticles for dual-pH-responsive codelivery of arsenic trioxide/paclitaxel against breast cancer cells. *Acta Pharmacol. Sin.* **42**, 832–842 (2021).
240. Zhang, H. *et al.* Co-delivery of nanoparticle and molecular drug by hollow mesoporous organosilica for tumor-activated and photothermal-augmented chemotherapy of breast cancer. *J. Nanobiotechnology* **19**, 1–13 (2021).
241. Zhang, M. *et al.* Co-delivery of etoposide and cisplatin in dual-drug loaded nanoparticles synergistically improves chemoradiotherapy in non-small cell lung cancer models. *Acta Biomater.* **124**, 327–335 (2021).
242. Zhang, Y. *et al.* Versatile metal-phenolic network nanoparticles for multitargeted combination therapy and magnetic resonance tracing in glioblastoma. *Biomaterials* **278**, 121163 (2021).
243. Amgoth, C. *et al.* Metal (Au)-Decorated Chitosan-l-Arginine Polymeric Vector for Codelivery of Gefitinib and miR125b for Lung Cancer Therapy. *ACS Appl. Polym. Mater.* **4**, 1675–1687 (2022).
244. Cai, H. *et al.* Stimuli-Sensitive Linear–Dendritic Block Copolymer–Drug Prodrug as a Nanoplatform for Tumor Combination Therapy. *Adv. Mater.* **34**, 1–15 (2022).
245. Chen, B. *et al.* iRGD Tumor-Penetrating Peptide-Modified Nano-Delivery System Based on a Marine Sulfated Polysaccharide for Enhanced Anti-Tumor Efficiency Against Breast Cancer. *Int. J. Nanomedicine* **17**, 617–633 (2022).
246. Cheng, X. *et al.* Enhanced tumor homing of pathogen-mimicking liposomes driven by R848 stimulation: A new platform for synergistic oncology therapy. *Acta Pharm. Sin. B* **12**, 924–938 (2022).
247. Deng, F. A. *et al.* Ce6- and Bez235-Based Nanomedicine for Chemo-/Photodynamic Combination Therapy of Tumors. *ACS Appl. Nano Mater.* **5**, 9277–9285 (2022).
248. Feng, M. *et al.* Multifunctional FeS2@SRF@BSA nanoplatform for chemo-combined photothermal enhanced photodynamic/chemodynamic combination therapy. *Biomater. Sci.* **10**, 258–269 (2022).
249. García-Hevia, L. *et al.* Magnetic lipid nanovehicles synergize the controlled thermal release of chemotherapeutics with magnetic ablation while enabling non-invasive monitoring by MRI for melanoma theranostics. *Bioact. Mater.* **8**, 153–164 (2022).
250. Hasannia, M. *et al.* Synthesis of doxorubicin-loaded peptosomes hybridized with gold nanorod for targeted drug delivery and CT imaging of metastatic breast cancer. *J. Nanobiotechnology* **20**, 1–27 (2022).
251. Hatami, E. *et al.* In Situ Nanoparticle Self-Assembly for Combination Delivery of Therapeutics to Non-Small Cell Lung Cancer. *ACS Appl. Bio Mater.* **5**, 1104–1119 (2022).
252. He, W. *et al.* Brain-Targeted Codelivery of Bcl-2/Bcl-xl and Mcl-1 Inhibitors by Biomimetic Nanoparticles for Orthotopic Glioblastoma Therapy. *ACS Nano* **16**, 6293–6308 (2022).

253. Hu, Q. *et al.* Combinational Chemoimmunotherapy for Breast Cancer by Codelivery of Doxorubicin and PD-L1 siRNA Using a PAMAM-Incorporated Liposomal Nanoplatform. *ACS Appl. Mater. Interfaces* **14**, 8782–8792 (2022).
254. Huang, J. *et al.* Targeted Drug/Gene/Photodynamic Therapy via a Stimuli-Responsive Dendritic-Polymer-Based Nanococktail for Treatment of EGFR-TKI-Resistant Non-Small-Cell Lung Cancer. *Adv. Mater.* **34**, 1–16 (2022).
255. Jiang, X. *et al.* Tumor-Activatable Nanoparticles Target Low-Density Lipoprotein Receptor to Enhance Drug Delivery and Antitumor Efficacy. *Adv. Sci.* **9**, 1–14 (2022).
256. Jin, T. *et al.* Engineering naphthalimide-cyanine integrated near-infrared dye into ROS-responsive nanohybrids for tumor PDT/PTT/chemotherapy. *Bioact. Mater.* **14**, 42–51 (2022).
257. Li, S. *et al.* Concurrent silencing of TBCE and drug delivery to overcome platinum-based resistance in liver cancer. *Acta Pharm. Sin. B* **13**, 967–981 (2023).
258. Li, Y. *et al.* Co-Delivery of Precisely Prescribed Multi-Prodrug Combination by an Engineered Nanocarrier enables Efficient Individualized Cancer Chemotherapy. *Adv. Mater.* **34**, 2110490 (2022).
259. Ma, X. *et al.* Bioresponsive immune-booster-based prodrug nanogel for cancer immunotherapy. *Acta Pharm. Sin. B* **12**, 451–466 (2022).
260. Nosrati, H. *et al.* Complete ablation of tumors using synchronous chemoradiation with bimetallic theranostic nanoparticles. *Bioact. Mater.* **7**, 74–84 (2022).
261. Qian, J. *et al.* Redox-Activatable Theranostic Co-Prodrug for Precise Tumor Diagnosis and Selective Combination Chemotherapy. *J. Med. Chem.* **65**, 10393–10407 (2022).
262. Sun, T. *et al.* A self-amplified ROS-responsive chemodrug–inhibitor conjugate for multi-drug resistance tumor therapy. *Biomater. Sci.* **10**, 997–1007 (2022).
263. Sun, Y. *et al.* Self-assembly nanomicelle-microneedle patches with enhanced tumor penetration for superior chemo-photothermal therapy. *Nano Res.* **15**, 2335–2346 (2022).
264. Tang, H. *et al.* Membrane-camouflaged supramolecular nanoparticles for co-delivery of chemotherapeutic and molecular-targeted drugs with siRNA against patient-derived pancreatic carcinoma. *Acta Pharm. Sin. B* **12**, 3410–3426 (2022).
265. Tao, W. *et al.* Artificial tumor microenvironment regulated by first hemorrhage for enhanced tumor targeting and then occlusion for synergistic bioactivation of hypoxia-sensitive platesomes. *Acta Pharm. Sin. B* **12**, 1487–1499 (2022).
266. Tarannum, M. *et al.* Advanced Nanoengineering Approach for Target-Specific, Spatiotemporal, and Ratiometric Delivery of Gemcitabine-Cisplatin Combination for Improved Therapeutic Outcome in Pancreatic Cancer. *Small* **18**, 2104449 (2022).
267. Wang, Y., Chen, L., Zhang, Z., Liu, W. & Li, L. Ratiometric co-delivery of doxorubicin and paclitaxel prodrug by remote-loading liposomes for the treatment of triple-negative breast cancer. *Drug Deliv. Transl. Res.* **12**, 2537–2549 (2022).
268. Wang, Z. *et al.* Intelligent Nanoparticles With pH-Sensitive Co-Delivery of Temozolomide and siEGFR to Ameliorate Glioma Therapy. *Front. Genet.* **13**, 921051 (2022).
269. Yang, K. *et al.* A Hybrid Supramolecular Polymeric Nanomedicine for Cascade-Amplified Synergetic Cancer Therapy. *Angew. Chemie - Int. Ed.* **61**, e202203786 (2022).
270. Yin, X. *et al.* Image-guided drug delivery of nanotheranostics for targeted lung cancer therapy. *Theranostics* **12**, 4147–4162 (2022).
271. Zeng, X. *et al.* Construction of pH-sensitive targeted micelle system co-delivery with curcumin and dasatinib and evaluation of anti-liver cancer. *Drug Deliv.* **29**, 792–806 (2022).
272. Zhang, N. *et al.* Co-Delivery of Doxorubicin and Anti-PD-L1 Peptide in Lipid/PLGA Nanocomplexes for the Chemo-Immunotherapy of Cancer. *Mol. Pharm.* **19**, 3439–3449 (2022).
273. Zhao, L. P. *et al.* Self-Delivery Nanomedicine for Glutamine-Starvation Enhanced Photodynamic Tumor Therapy. *Adv. Healthc. Mater.* **11**, e2102038 (2022).
